# Identification of Key eRNAs for Spinal Cord Injury by Integrated Multinomial Bioinformatics Analysis

**DOI:** 10.3389/fcell.2021.728242

**Published:** 2021-10-11

**Authors:** Runzhi Huang, Siqiao Wang, Rui Zhu, Shuyuan Xian, Zongqiang Huang, Liming Cheng, Jie Zhang

**Affiliations:** ^1^Key Laboratory of Spine and Spinal Cord Injury Repair and Regeneration (Tongji University), Ministry of Education, Shanghai, China; ^2^Division of Spine Surgery, Department of Orthopedics, Tongji Hospital, Tongji University School of Medicine, Shanghai, China; ^3^Tongji University School of Medicine, Shanghai, China; ^4^Department of Orthopedics, The First Affiliated Hospital of Zhengzhou University, Zhengzhou, China

**Keywords:** spinal cord injury, multi-omics bioinformatics analysis, biomarkers, eRNA regulatory network, therapeutic targets

## Abstract

**Background:** Spinal cord injury (SCI) is a severe neurological deficit affecting both young and older people worldwide. The potential role of key enhancer RNAs (eRNAs) in SCI remains elusive, which is a prominent challenge in the trauma repair process. This study aims to investigate the roles of key eRNAs, transcription factors (TFs), signaling pathways, and small-molecule inhibitors in SCI using multi-omics bioinformatics analysis.

**Methods:** Microarray data of peripheral blood mononuclear cell (PBMC) samples from 27 healthy volunteers and 25 chronic-phase SCI patients were retrieved from the Gene Expression Omnibus database. Differentially expressed transcription factors (DETFs), differentially expressed enhancer RNAs (DEeRNAs), and differentially expressed target genes (DETGs) were identified using the Linear Models for Microarray Data (limma) package. Fraction of immune cells was estimated using CIBERSORT algorithm. Gene Set Variation Analysis (GSVA) was applied to identify the downstream signaling pathways. The eRNA regulatory network was constructed based on the correlation results. Connectivity Map (CMap) database was used to find potential drugs for SCI patients. The cellular communication analysis was performed to explore the molecular regulation mechanism of SCI based on single-cell RNA sequencing (scRNA-seq) data. Chromatin immunoprecipitation sequencing (ChIP-seq) and Assay for Transposase-Accessible Chromatin using sequencing (ATAC-seq) data were used to validate the key regulatory mechanisms. scRNA-seq dataset was used to validate the cell subtype localization of the key eRNAs.

**Results:** In total, 21 DETFs, 24 DEeRNAs, and 829 DETGs were identified. A regulatory network of 13 DETFs, six DEeRNAs, seven DETGs, two hallmark pathways, two immune cells, and six immune pathways was constructed. The link of Splicing factor proline and glutamine rich (SFPQ) (TF) and vesicular overexpressed in cancer prosurvival protein 1 (VOPP1) (eRNA) (*R* = 0.990, *p* < 0.001, positive), VOPP1 (eRNA) and epidermal growth factor receptor (EGFR) (target gene) (*R* = 0.974, *p* < 0.001, positive), VOPP1, and T helper (Th) cells (*R* = −0.987, *p* < 0.001, negative), and VOPP1 and hallmark coagulation (*R* = 0.937, *p* < 0.001, positive) was selected. Trichostatin A was considered the best compound target to SCI-related eRNAs (specificity = 0.471, *p* < 0.001).

**Conclusion:** VOPP1, upregulated by SFPQ, strengthened the transient expression of EGFR. Th cells and coagulation were the potential downstream pathways of VOPP1. This regulatory network and potential inhibitors provide novel diagnostic biomarkers and therapeutic targets for SCI.

## Introduction

Spinal cord injury (SCI) refers to functional or structural injury of the spinal cord causing total or partial loss of motor, sensory, and sphincter function below the injured segment (Nakae et al., 2011; Anderson et al., 2018). Whether the pathogenesis is disease or trauma, SCI exhibits high disability rates. It affects approximately 347,000 individuals in the United States, with about 17,500 new cases diagnosed every year (Badhiwala et al., 2019; GBD 2016 Neurology Collaborators, 2019). According to the illness course, SCI can be divided in three phases: the acute phase (0–15 days), the subacute phase (3–5 months), and the chronic phase (6–12 months) (Pouw et al., 2011). The most common pathological characteristics of SCI are a cascade of molecular and cellular events triggered by inflammation, and excitotoxicity impairs endogenous regeneration, namely, axonal outgrowth and remyelination.

It is arduous to repair injured neurons and restore conducting function of axons, so treatments of SCI have become worldwide problems (Borton et al., 2014). Importantly, there are no efficacious drugs or therapeutic approaches for SCI. Thus, many patients suffer substantial physical and psychological consequences. To date, the molecular mechanisms of SCI remain unclear, so it is difficult to develop novel drugs or treatments. Thus, it is urgent to determine specific molecular mechanisms that underlie the pathogenesis of SCI.

The gene mutation and aberrant expression are implicated in the development of numerous diseases and may involve various types of regulatory molecules. Enhancer refers to a kind of distal regulatory DNA element that is able to enhance the transcription of corresponding target genes *via* coordinating with target gene promoters (Blackwood and Kadonaga, 1998). Widely acknowledged as DNA elements that nucleate transcription factor (TF) binding, enhancers were identified to also transcribe non-coding RNAs recently, which are referred to as enhancer RNAs (eRNAs) (Kim et al., 2010). Numerous eRNAs have been identified in *Homo sapiens* so far, and many of them were suggested to play crucial parts in the transcriptional circuitry (Li et al., 2016).

Inflammation after SCI is a fundamental basis of secondary damage. Inflammatory response is coordinated by various signaling modalities that include the epigenetic modification of promoters and eRNAs (Rudman et al., 2018). Large amounts of cytokines are quickly generated and released from various cells in the damaged structures after SCI (Hayashi et al., 2000; Pineau and Lacroix, 2007). Cytokines activate apoptosis of pathological nerve cells and recruitment of active leukocytes in myelopathic lesions (Beck et al., 2010; Chen et al., 2011). Leukocyte infiltration contributes to cascaded amplification of inflammation cytokine signaling, increasing neurotoxicity, and thus promoting fibrotic scar formation *via* recruiting fibroblasts in damaged regions (Zhu et al., 2015). In addition, damaged areas chronically maintain pro-inflammatory phenotype after injury, hindering regeneration and healing of the injured spinal cord (Beck et al., 2010). Because of the detrimental inflammatory effects after SCI, reducing inflammatory response is a major demand for improving clinical outcomes of SCI patients. Nevertheless, eRNA-targeted anti-inflammation agents that effectively inhibit inflammation and improve the prognosis of SCI patients are still insufficient, and novel anti-inflammation interventions are urgently needed.

In this study, transcription factors (TFs), differentially expressed enhancer RNAs (DEeRNAs), and target genes in patients with SCI were identified. Significant immune cells and immune-related pathways were identified by cell type identification by estimating relative subsets of RNA transcripts (CIBERSORT) and single-sample Gene Set Enrichment Analysis (ssGSEA) algorithms, respectively. In addition, TFs, eRNAs, target genes, immune cells, immune-related gene sets, hallmark gene sets (signaling pathways) acquired by the Gene Set Variation Analysis (GSVA) were merged in correlation analysis. Only remarkable interactions were extracted for construction of eRNA regulatory network. Moreover, we performed Connectivity Map (CMap) analysis to explore the potential inhibitors specific to SCI-related eRNAs. Furthermore, chromatin immunoprecipitation sequencing (ChIP-seq) and Assay for Transposase-Accessible Chromatin using Sequencing (ATAC-seq) data were utilized for validation of the key regulation mechanisms in this network.

## Materials and Methods

### Data Acquisition

The present study was endorsed by the Ethics Committee of the Shanghai Tongji Hospital affiliated to Tongji University. Microarray data and clinical information of 27 peripheral blood mononuclear cell (PBMC) samples from healthy volunteers and 25 PBMC samples from SCI patients were obtained from the Gene Expression Omnibus (GEO) database (accession number: GSE82152)^1^ (Barrett et al., 2011) and E-GEOD-69901 (ArrayExpress)^2^ (Athar et al., 2019), respectively. In addition, these two batches of microarray data were both retrieved from a platform called GPL21975 [PrimeView] Affymetrix Human Gene Expression Array [Brainarray ENTREZG Version 17],^3^ compared with data merging based on multiple different platforms, and resulted in less error. TFs were obtained from the Cistrome Cancer database^4^ (Zheng et al., 2019). Immunologically related genomic expression profiles were downloaded from the ImmPort database^5^ (Bhattacharya et al., 2014). Hallmark signaling pathways were collected from the Molecular Signatures Database (MSigDB, v7.2)^6^ (Liberzon et al., 2015). ChIP-seq data of H3K27ac (accession number: GSE134744) and ATAC-seq data of key eRNAs (accession number: GSE139099) were obtained from the GEO database (see text foot note 1) (Barrett et al., 2011).

### Data Preprocessing and Differential Expression Analysis

Samples and patients with incomplete clinical information were excluded. All original microarray data were read using affy package, followed by robust multi-array average (RMA) background correction, standardization, probe-specific background correction, and summarizing probe set values in one expression measure (Irizarry et al., 2003; Gautier et al., 2004). Then, two batches of microarray data aforementioned were corrected using normalizeBetweenArrays function in Linear Models for Microarray Analysis (limma) package, which were then merged for differential expression analysis. Differential expression analysis of eRNAs (DEeRNAs), target genes (DETGs) and TFs (DETFs) between SCI and normal blood was conducted using the Linear Models for Microarray Data (limma) package (Smyth, 2004). For *p*-value, false discovery rate (FDR) was utilized for multiple testing correction. Absolute log_2_[Fold Change (FC)] ≥ 1.0 and the FDR < 0.05 were cutoff criteria.

### Functional Enrichment Analysis

We conducted Gene Ontology (GO) and Kyoto Encyclopedia of Genes and Genomes (KEGG) enrichment analyses to identify biological processes and pathways that were most related to DEeRNAs with FDR *p*-value < 0.05 as cutoff value (Zhang T. et al., 2013).

### Identification of Potential Immune Cells, Immune Pathways, and Hallmark Pathways

Immune cell proportions in the SCI and normal samples were analyzed utilizing the cell type identification by estimating relative subsets of RNA transcripts (CIBERSORT) algorithm (Newman et al., 2015). To identify the association between eRNA signature and immune cell infiltration in SCI tissues, we uploaded gene expression matrix data to CIBERSORT database^7^ to purify cellular subtype-specific gene expression. Infiltrating immune cells were all extracted for further analysis. Furthermore, non-parametric tests were used to identify correlations between these immune cells/pathways and various clinical phenotypes.

In addition, ssGSEA algorithm was conducted to evaluate and quantify the enrichment level of 10 immune-related pathways in SCI samples (Xiao et al., 2020). Furthermore, Pearson correlation analysis was carried out to determine the correlations between key eRNAs and immune-related signaling pathways or immune genes, in which a *p*-value < 0.05 was considered statistically significant.

### Identification of Potential Downstream Hallmark Pathways

GSVA (Ferreira et al., 2021) and GSEA (Subramanian et al., 2007) were both utilized for exploring potential downstream hallmark pathways of DEeRNAs. Absolute quantification of 50 hallmark signaling pathways was evaluated to extract differentially expressed hallmark pathways between SCI and normal blood using ClusterProfiler package and GSVA package (Yu et al., 2012; Hanzelmann et al., 2013). GSEA was carried out to reveal the significant enrichment of upregulated and downregulated hallmark pathways in SCI and normal blood. Furthermore, correlations of hallmark pathways of GSVA and GSEA were extracted, and the interactional pathways were suggested as key pathways.

### Construction of Regulation Network for Transcription Factors, Enhancer RNAs, Immune Cells, Immune-Related Pathways, Target Genes, and Hallmark Gene Sets

Pearson correlation analysis was conducted based on key TFs, eRNAs, immune genes, immune cells, target genes, and hallmark pathways aforementioned. An eRNA regulatory network was then reconstructed by Cytoscape (3.7.1) (Kohl et al., 2011).

Molecular complex detection (MCODE) plugin of Cytoscape software was utilized to identify significant molecules within this network in a recognition standard MCODE score ≥ 4 to extract the modules of hub genes. Interaction relationships between key eRNAs and other components were controlled based on *p*-value < 0.05 and | correlation coefficient| > 0.40.

### Connectivity Map Analysis

Here, we used CMap (build 02) to find potential inhibitors that may target SCI-related eRNAs. In total, 6,100 gene expression cases covering 1,309 drugs were obtained from the CMap database^8^ (Lamb et al., 2006). That is, a candidate drug might correspond to various gene expression cases. Genes in each case were ranked *via* taking differential expression values between drug-untreated and drug-treated cell lines, and 6,100 gene lists related to drugs were then generated. Based on identified key DEeRNAs involved in SCI and 6,100 drug-related cases, we conducted a non-parametric test to explore the relationship between drugs and SCI.

Information on targeting compounds is available in the mechanism of actions (MoA)^9^ that includes transcriptional responses of various human cell lines to perturbagens, structural formulas, and protein targets. On the basis of MoA, compounds that may target SCI-related eRNAs/enhancers in this study were extracted.

### Single-Cell RNA Sequencing Data Processing

Following the procedure of 10x Genomics Chromium^10^ (Chen et al., 2020), the preprocessing of samples and scRNA-seq data was conducted. After demultiplexing, the sequencing results were divided into two pair-ended reads fastq files that were then trimmed to eliminate template switch oligo (TSO) sequence and polyA tail sequence. Additionally, clean reads were aligned with the GRCh38 (Version: 100) genome assembly, which were quantified using the Cell Ranger Software (Version 1.0.0).^11^

The quantitative gene expression matrices (The row names of matrices were genes and column names were barcodes) acquired from seven libraries that included seven vertical section samples and 14 cross-section samples were analyzed with Seurat pipeline (Version: 3.2.2) for further analysis (Butler et al., 2018). Only cells with less than 10% mitochondrial gene mapped and more than 100,000 transcripts expressing were extracted for subsequent analysis. In addition, genes that expressed in over three single cells were included in follow-up analyses. After the completion of quality control (QC), all samples were merged in one Seurat object with the function of “IntegrateData” that were then scaled and standardized with the function of “ScaleData.” Top 1,500 variable genes were identified using “vst” method. To reduce model dimensionality, principal component (PC) analysis (PCA) was initially carried out, and the top 20 PCs were incorporated as input file for Uniform Manifold Approximation and Projection (UMAP) for dimension reduction analysis. The UMAP plots that illustrated cell subclusters were constructed using the “DimPlot” and “RunUMAP” function.

### Differential Expression Analysis of Single-Cell RNA Sequencing

Genes with remarkable differential expression from the top 1,500 variable genes were defined as differentially expressed genes (DEGs) with “wilcox” method using “FindAllMarkers” function.

### Cell Type Annotation

To identify the cell type of each unsupervised cluster, DEGs of all subclusters were utilized as potential references that were combined with known specific cell surface biomarkers obtained from CellMarker^12^ (Zhang et al., 2019) for a comprehensive annotation of cell type. Given the variable gene expression patterns in SCI, a specific cellular annotation method was utilized in the present study. Firstly, known cell surface biomarkers of neuron (Rph3a, Tubb3, Gnal), neuron precursor cell (NPC) (Sox2, Prom1, Sox9), astrocyte (Gfap, S100b, Vim), fibroblast (Col1a1, Col1a2, Col4a1), oligodendrocyte (Mbp, Olig2, Gjb1), oligodendrocyte precursor cell (OPC) (Olig1, Apoc4, Epn2), microglia (Cx3cr1, Aif1, Itgam), and macrophage (Cd68, Itgam, Fcgr3) were used to annotate the cell type of the eight cells aforementioned. Furthermore, because several biomarkers of macrophage and microglia were overlapping, cells with transcripts per million (TPM) of Cd68 < 2 and Aif1 > 1.38 were identified as microglia, while cells with TPM of Cd68 > 2 were identified as macrophages. In addition, the cellular feature plots, dot plots, violin plots, and heat maps were constructed to show the marker genes of each cell type using SCANPY (Version: 1.7.1) and Seurat R package (Version: 3.2.2) under the environment of Python 3.6 (Butler et al., 2018; Wolf et al., 2018).

### Cellular Communication Analysis

In order to elucidate the significant cellular communication patterns and ligand–receptor pairs among various different cell types in the spinal cord, cellular communication analysis was carried out using iTALK R package (Version: 0.1.0)^13^ (Wang et al., 2019). Firstly, because of the scarcity of musculus resources in present mainstream cellular communication algorithms, the top 1,500 variable genes were transformed to human genes using biomaRt package (Version: 2.46.0) in a certain homologous degree with “getLDS” function (Durinck et al., 2009). Secondly, the normalized expression matrix of these genes was incorporated into the iTALK object with the “rawParse” function. Finally, the top 200 ligand–receptor pairs were shown by ligand–receptor plots and iTALK networks.

### Chromatin Immunoprecipitation Sequencing Validation

Histone H3K27ac was implicated in enhancer-specific modifications, which were essential for enhancers to activate the transcription of relevant target genes (Kang et al., 2021). Role of H3K27ac in eRNA transcription was evaluated by analyzing ChIP-seq data (accession number: GSE134744) in peripheral blood (Grubert et al., 2020).

The ChIP-seq data of Splicing factor proline and glutamine rich (SFPQ) (accession number: GSM2827312, GSM1411215, GSM2825596, GSM1097497, GSM2827311, and GSM1097496) (Dunham et al., 2012; ENCODE Project Consortium, 2012; West et al., 2014) and H3K27 (accession number: GSM732912, GSM575294, and GSM663427) (Lister et al., 2009; Guenther et al., 2010; Wang et al., 2011) were obtained from the Cistrome database for validating binding relationships between key eRNAs and other significant biomarkers in this study. Furthermore, we determined the eRNA binding relationships using the University of California Santa Cruz (UCSC) Genome Browser^14^ based on the original ChIP-seq data (Robinson et al., 2011; Li et al., 2015).

### Assay for Transposase-Accessible Chromatin Using Sequencing Validation

The ATAC-seq (Buenrostro et al., 2013) refers to an impressively flexible, simple, and powerful technique to profile chromatin regions genome-wide compared to traditional methods like functional assays or sequence conservation analyses.

We downloaded peak data of key TFs and eRNAs (accession number: GSE139099) (Grubert et al., 2020) and performed the correlation analysis using ggpubr.^15^ In order to explore the potential super-enhancers, we performed the peak calling with macs14 (Feng et al., 2012).^16^ Furthermore, we ranked enhancers and determined super-enhancers using ROSE (Whyte et al., 2013).^17^ Multiple SCI-enriched ATAC-seq peaks that represented candidate regulation elements were located near established eRNAs and were enriched in distinct sets of TF binding sites.

### Online Single-Cell RNA Sequencing Validation

To identify the expression of the key biomarkers in single-cell level, scRNA-seq data of developing mouse spinal cord (accession number: GO0006836 and GO0098609) were downloaded from Single Cell Expression Atlas (Delile et al., 2019).^18^ Then, we validated the cellular location of these biomarkers’ expression.

### Statistics Analysis

All statistical analyses were put into effect using R version 3.6.1 (Institute for Statistics and Mathematics, Vienna, Austria).^19^ In descriptive statistics, mean ± standard deviation was utilized for continuous variables in normal distribution.

In addition, when encountering continuous variables in abnormal distribution, the median was utilized. Two-sided *p*-value < 0.05 was suggested to be necessary for statistics.

## Results

### Identification of Differentially Expressed Enhancer RNAs

**FIGURE 1:**
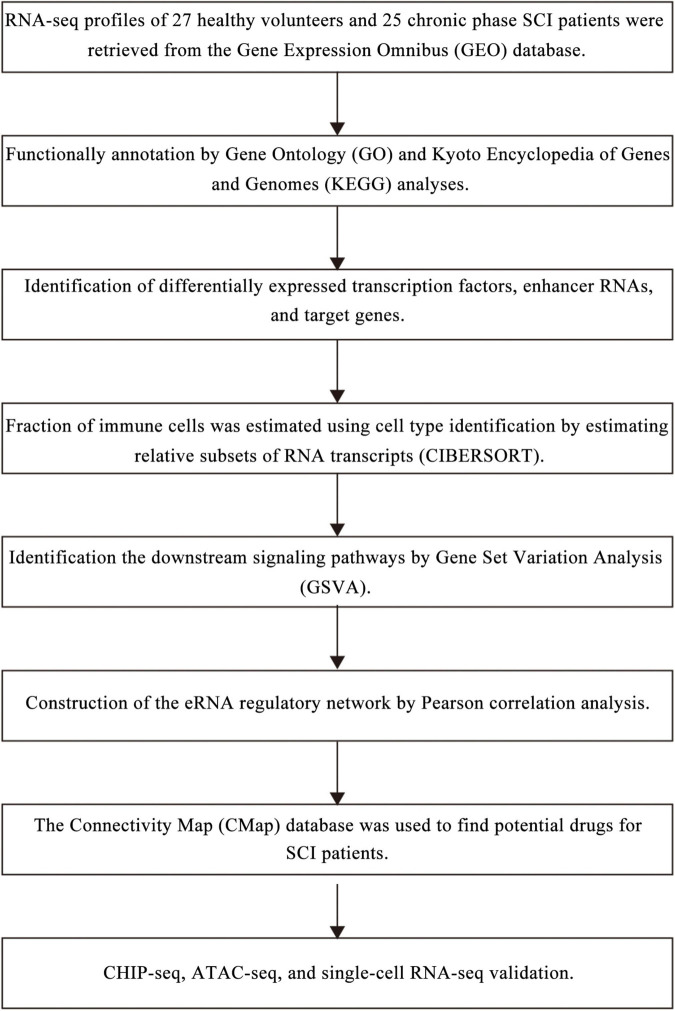
The analysis flowchart of all analysis processes.

**FIGURE 2:**
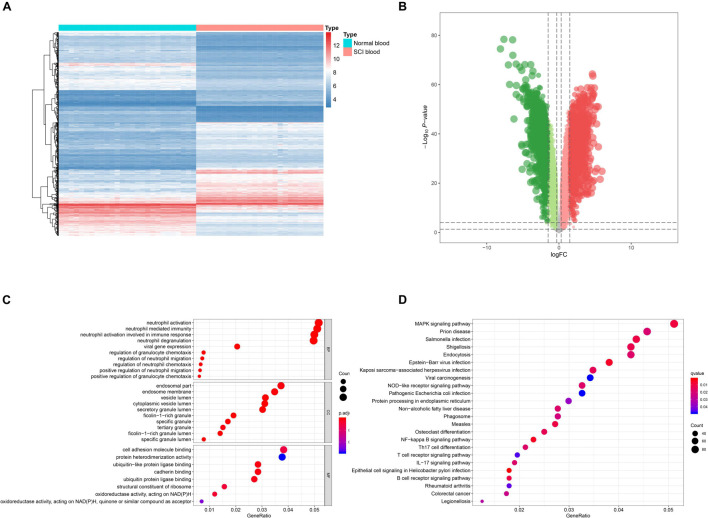
Identification of differentially expressed enhancer RNAs (DEeRNAs) and functional annotation. **(A)** Heat map showing the expression level of DEeRNAs. **(B)** Volcano plot showing the *p*-value and log| FC| value of DEeRNAs by the differential expression analysis above. **(C)** The output of Gene Ontology (GO) analysis. **(D)** The output of Kyoto Encyclopedia of Genes and Genomes (KEGG) pathways enrichment analysis.

Analysis process of the present study was illustrated in Figure 1. Samples and patients with incomplete clinical information were excluded, and conformers were shown in Supplementary Table 1. Based on the threshold, a total of 3,979 eRNAs were identified as DEeRNAs between 27 healthy volunteers and 25 SCI patients from 5,100 eRNAs, which were shown in the Heat map (Figure 2A) and volcano plot (Figure 2B).

### Functional Enrichment Analysis

**FIGURE 3:**
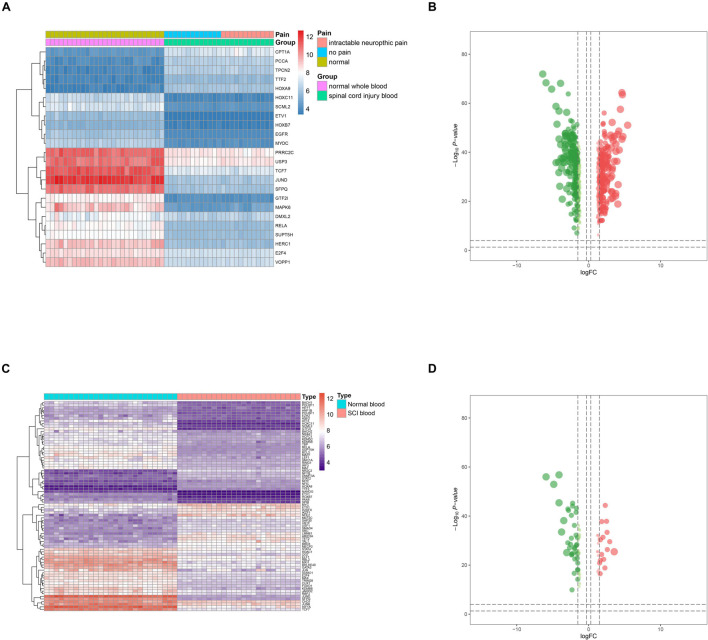
Identification of differentially expressed spinal cord injury (SCI)-related enhancer RNAs (eRNAs) and transcription factors (TFs). **(A)** Heat map showing the expression level of differentially expressed SCI-related eRNAs. **(B)** Volcano plot showing the *p*-value and log| FC| value of differentially expressed SCI-related eRNAs. **(C)** Heat map showing the expression level of differentially expressed TFs. **(D)** Volcano plot showing the *p*-value and log| FC| value of differentially expressed TFs.

GO and KEGG enrichment analyses were both conducted to explore the potential mechanism of identified DEeRNAs. In GO analysis, the most important terms in biological process (BP), cellular component (CC), and molecular function (MF) were neutrophil activation, endosomal part, and cell adhesion molecule binding (Figure 2C). The most significant KEGG pathway was mitogen-activated protein kinase (MAPK) signaling pathway (Figure 2D). In the heat map (Figure 3A) and volcano plot (Figure 3B), 24 differentially expressed SCI-related eRNAs were identified, which were considered to be key eRNAs in patients with SCI.

### Correlation Analysis of Key Differentially Expressed Enhancer RNAs and Differentially Expressed Transcription Factors

Twenty-one DETFs (log2 FC > 1 or < −1 and FDR < 0.05) were identified using limma package that were shown in the heat map (Figure 3C) and volcano plot (Figure 3D). In addition, to explore the relationship between DETFs and DEeRNAs, Pearson correlation analysis was carried out, and 27 regulatory relationships were identified (correlation coefficient < −0.300 or > 0.300, and *p*-value < 0.001). Based on the correlation analysis results for regulation relationships of key DETFs and DEeRNAs, link of SFPQ (TF) and vesicular overexpressed in cancer prosurvival protein 1 (VOPP1, eRNA) was extracted (*R* = 0.990, p < 0.001, positive).

### Correlation Analysis of Key Differentially Expressed Enhancer RNAs and Differentially Expressed Target Genes

**FIGURE 4:**
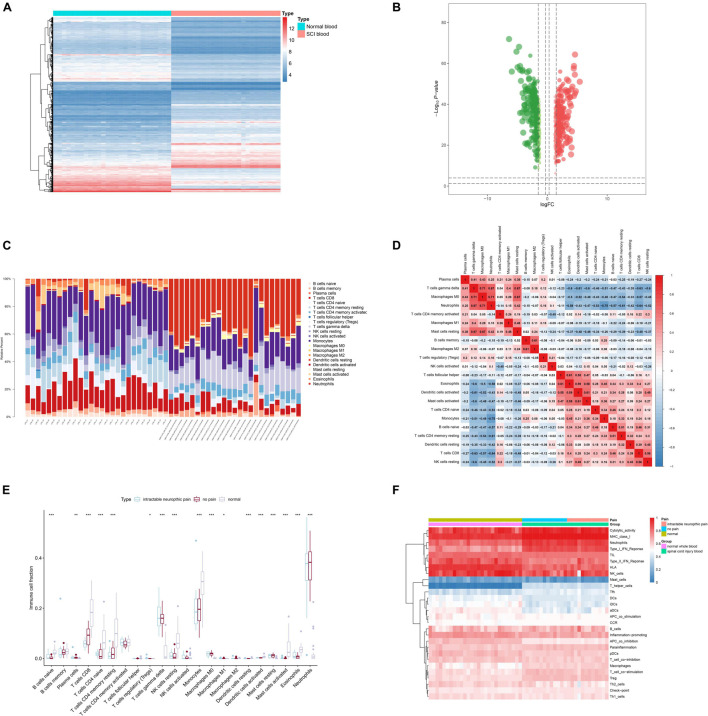
Identification of differentially expressed target genes (DETGs) and evaluation of immune cells/gene sets. **(A)** Heat map showing the expression level of DETGs. **(B)** Volcano plot showing the *p*-value and log| FC| value of DETGs. **(C)** Bar chart showing the percentage of 22 kinds of immune cells from healthy volunteers and spinal cord injury (SCI) samples by cell type identification by estimating relative subsets of RNA transcripts (CIBERSORT). **(D)** Heat map showing the expression level of 22 immune cells by CIBERSORT. **(E)** Box plot showing the fraction of 22 immune cells from healthy volunteers and SCI samples by non-parametric tests. **(F)** Heat map showing the expression level of 28 immune cells/pathways *via* single-sample Gene Set Enrichment Analysis (ssGSEA).

In total, 829 DETGs (log2 FC > 1 or < −1 and FDR < 0.05) were identified using limma package and illustrated in the heat map (Figure 4A) and volcano plot (Figure 4B). Furthermore, to determine the relationships between the identified DETGs and DEeRNAs, eight regulation relationships were identified using Pearson correlation analysis (correlation coefficient < −0.300 or > 0.300 and *p*-value < 0.001). Additionally, based on significant regulation relationships of key DEeRNAs and DETGs acquired by Pearson analysis, the link of VOPP1 (eRNA) and epidermal growth factor receptor (EGFR, target gene) was extracted (R = 0.974, p < 0.001, positive).

### Correlation Analysis of Key Differentially Expressed Enhancer RNAs, Immune Cells/Pathways

Twenty-two types of immune cells/pathways were identified using CIBERSORT. The bar chart showed the percentage of 22 kinds of immune cells in 27 healthy volunteers and 25 SCI patients (Figure 4C). The heat map showed the expression level of 22 immune cells by CIBERSORT (Figure 4D). Results of non-parametric tests demonstrated significant correlation between immune cells/pathways and SCI (Figure 4E). Furthermore, ssGSEA was conducted to identify 28 immune cells/pathways that were significantly correlated with differentially expressed SCI-related eRNAs. Results of ssGSEA were shown in the heat map (Figure 4F).

Then, by co-analyzing VOPP1 and 22 types of immune cells or pathways, the top 3 immune cells or immune-related pathways were extracted for further analysis, which included Antigen-presenting cells (APC) co-inhibition (*R* = 0.936, *p* < 0.001, positive), MHC class I (*R* = −0.973, *p* < 0.001, negative), and T helper (Th) cells (*R* = −0.987, *p* < 0.001, negative). Th cells were finally extracted in further analyses.

### Identification of Potential Downstream Hallmark Pathways of Key Differentially Expressed Enhancer RNAs

**FIGURE 5:**
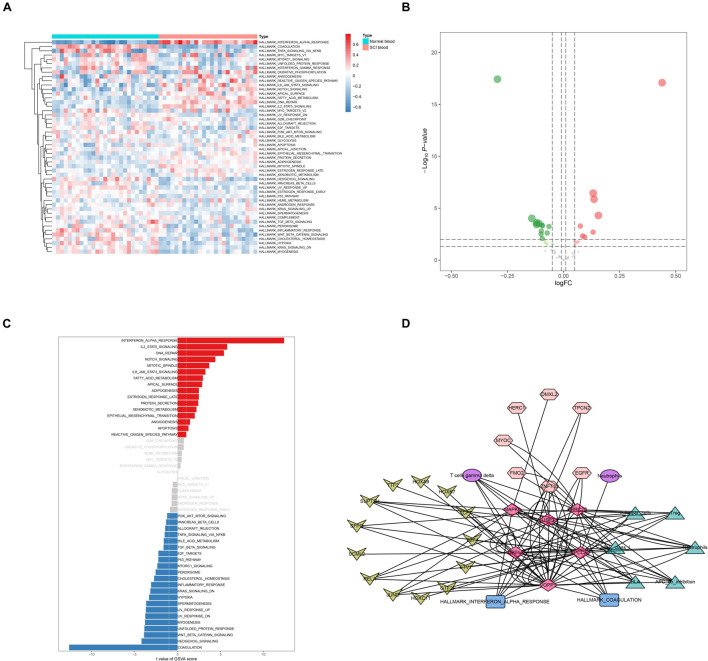
Identification of key hallmark pathways. **(A)** Heat map showing the co-expression level of hallmark sets in spinal cord injury (SCI) samples by Gene Set Variation Analysis (GSVA). **(B)** Volcano plot showing the co-expression level of hallmark sets in SCI samples by GSVA. **(C)** The bar plot revealing the *t*-value of GSVA score. **(D)** Overview of the protein–protein interaction network of OA-related enhancer RNAs (eRNAs), translational factors (TFs), immune cells/pathways, and hallmark gene sets. The diamonds represented key eRNAs. The arrows represented TFs. The ellipses represented immune cells. The triangles represented immune pathways. The hexagons represented target genes. The rectangle represented hallmark gene sets.

Results of GSVA (| log2 FC| > 0.1, p < 0.05) were shown in the heat map (Figure 5A). The volcano plot showed 28 differentially expressed hallmark pathways between 27 healthy volunteers and 25 SCI patients (Figure 5B). The *t*-value of GSVA score of these pathways was shown in the *t*-test bar chart (Figure 5C). Furthermore, 38 hallmark pathways were extracted from the 50 hallmark pathways for further analysis, controlled by cutoff values of | log2 FC| > 0.1 and *p* < 0.05. Importantly, VOPP1 and hallmark coagulation (*R* = 0.937, *p* < 0.001, positive) showed the strongest interaction.

### Construction of the Enhancer RNA Regulation Network

**FIGURE 6:**
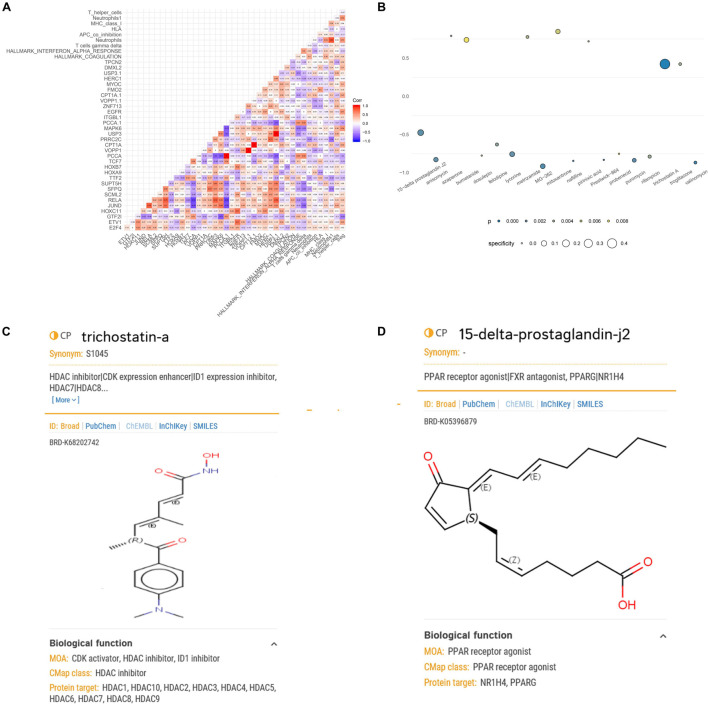
Correlation analysis of the key biomarkers and identification of potential small-molecule drugs for spinal cord injury (SCI). **(A)** Heat map showing the results of correlation analysis (Pearson analysis) of enhancer RNAs (eRNAs), transcription factors (TFs), target genes, immune cells/pathways, and hallmark pathways. **(B)** Heat map showing perturbagens from the Connectivity Map (CMap) that might be capable of targeting SCI-related eRNAs. **(C)** Structural formula of trichostatin A. **(D)** Structural formula of 15-delta prostaglandin J2.

A co-expression regulation network of key DETFs, DEeRNAs, DETGs, immune cells/pathways, and hallmark pathways was constructed, which elaborated on the regulatory relationships among the aforementioned components (Figure 5D). Furthermore, to quantify the interaction coefficients among them, co-expression analysis was performed at the transcriptional level (Figure 6A).

### Connectivity Map Analysis

Because eRNAs were implicated in the pathological processes of SCI and traditional long-term treatment with drugs may result in severe side effects and/or insufficient inflammation and pain relief, it is urgent to find potential compounds that target SCI-related eRNAs. Through exploring potential compounds in CMap database, we identified 19 compounds that have been validated to target SCI-related eRNAs in multiple clinical trials, including 15-delta prostaglandin J2, anisomycin, azaperone, bumetanide, dosulepin, felodipine, lycorine, metrizamide, MG-262, mitoxantrone, naftifine, pirinixic acid, Prestwick-864, probenecid, puromycin, rifampicin, trichostatin A (TSA), troglitazone, and valinomycin (Figure 6B). TSA (specificity = 0.471, *p* < 0.001) and 15-delta prostaglandin J2 (specificity = 0.128, *p* < 0.001) with the highest specificity were considered the best compounds to target SCI-related eRNAs (Figures 6C,D), and TSA was extracted for further analysis.

### Integrated Analysis of Single-Cell RNA Sequencing

**FIGURE 7:**
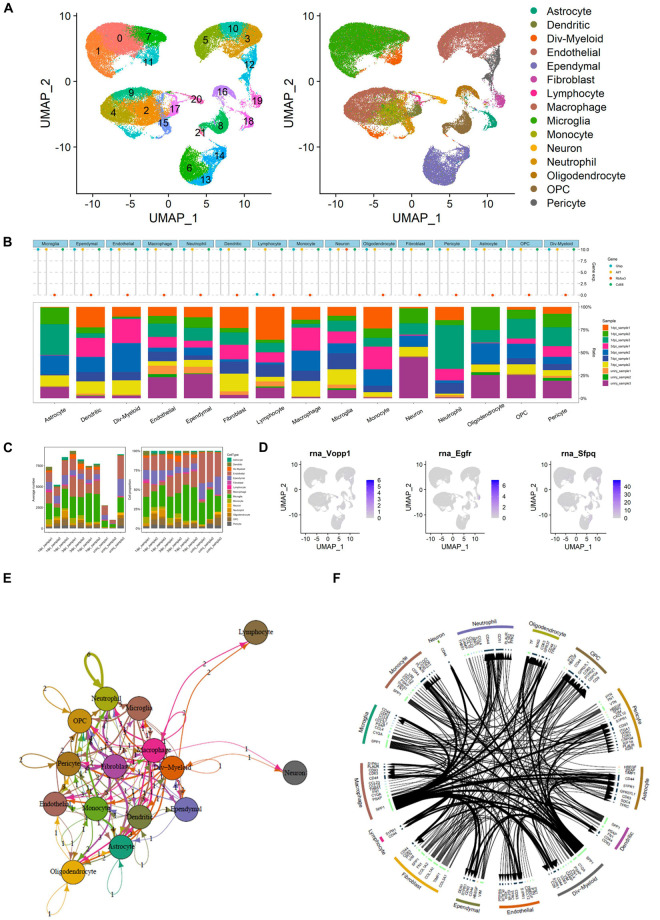
Integrated analysis of single-cell RNA sequencing (scRNA-seq) and cellular communication. **(A)** Uniform Manifold Approximation and Projection (UMAP) scatter plots and cellular feature plots showing the cell types and marker genes of each cell type. **(B)** Dot plot showing gene expression levels of the most significant differentially expressed genes (DEGs) of multiple inflammation cells and neuron. In addition, the proportion of these cells in different spinal cord injury (SCI) samples was also shown in the bar plot. **(C)** Bar plots showing the average number and cell proportion of these cells. **(D)** Feature plots of vesicular overexpressed in cancer prosurvival protein 1 (VOPP1), epidermal growth factor receptor (EGFR), and Splicing factor proline and glutamine rich (SFPQ). **(E)** Network showing the intersected cellular communication genes. **(F)** Circle plot showing the intersected cellular communication genes.

The UMAP scatter plots and cellular feature plots showed the cell types and marker genes of different cell types (Figure 7A). Furthermore, the top 1,500 variable genes (| log2(FC)| > 0.5 and FDR < 0.05) were extracted by DEG analysis. Gene expression levels of the most significant DEGs of multiple inflammation cells (astrocyte, dendritic, Div-myeloid, endothelial, ependymal, fibroblast, lymphocyte, macrophage, microglia, monocyte, neutrophil, oligodendrocyte, OPC, and pericyte) and neuron were illustrated in Figure 7B. In addition, the proportion of these cells in different SCI samples was also shown in the bar plot (Figure 7B). Specifically, average number and cell proportion of these cells were illustrated in Figure 7C. The feature plots of VOPP1, EGFR, and SFPQ were shown in Figure 7D. Importantly, VOPP1 was significantly highly expressed in lymphocytes, which was consistent with the previous analysis. In addition, EGFR and SFPQ were highly expressed in fibroblast and microglia, respectively. Finally, intersected cellular communication network and ligand–receptor plot showed the mechanisms of intercellular signal transduction, and fibroblast was the core cellular component among these intersected ligand–receptor pairs (Figures 7E,F). It showed that lymphocytes received signals from myeloid cells and various neurocytes including microglia and then played a role in the downstream pathological process of SCI.

**FIGURE 8:**
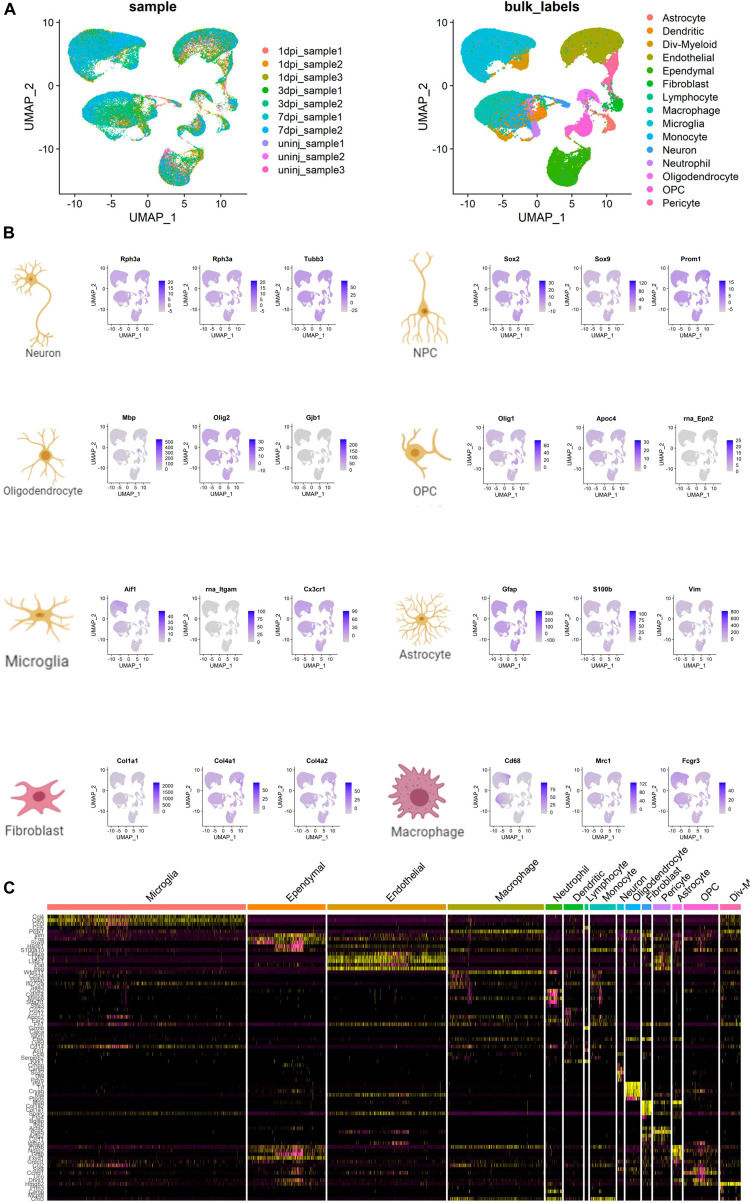
Cell type annotation and cellular communication analysis. **(A)** Uniform Manifold Approximation and Projection (UMAP) scatter plots and cellular feature plots showing the cell types and marker genes for each cell type. **(B)** Cellular feature plots showing marker genes of different cell types. **(C)** Heat map showing the most significant differentially expressed genes (DEGs) of neuron and inflammation cells.

The UMAP scatter plots and cellular feature plots showed various cell types and marker genes for corresponding cell types, respectively (Figure 8A). Cellular feature plots of marker genes in different cell types were shown in Figure 8B. The most significant DEGs of neuron and inflammation cells were annotated on the DEG heat map (Figure 8C).

### Chromatin Immunoprecipitation Sequencing and Assay for Transposase-Accessible Chromatin Using Sequencing Validation

**FIGURE 9:**
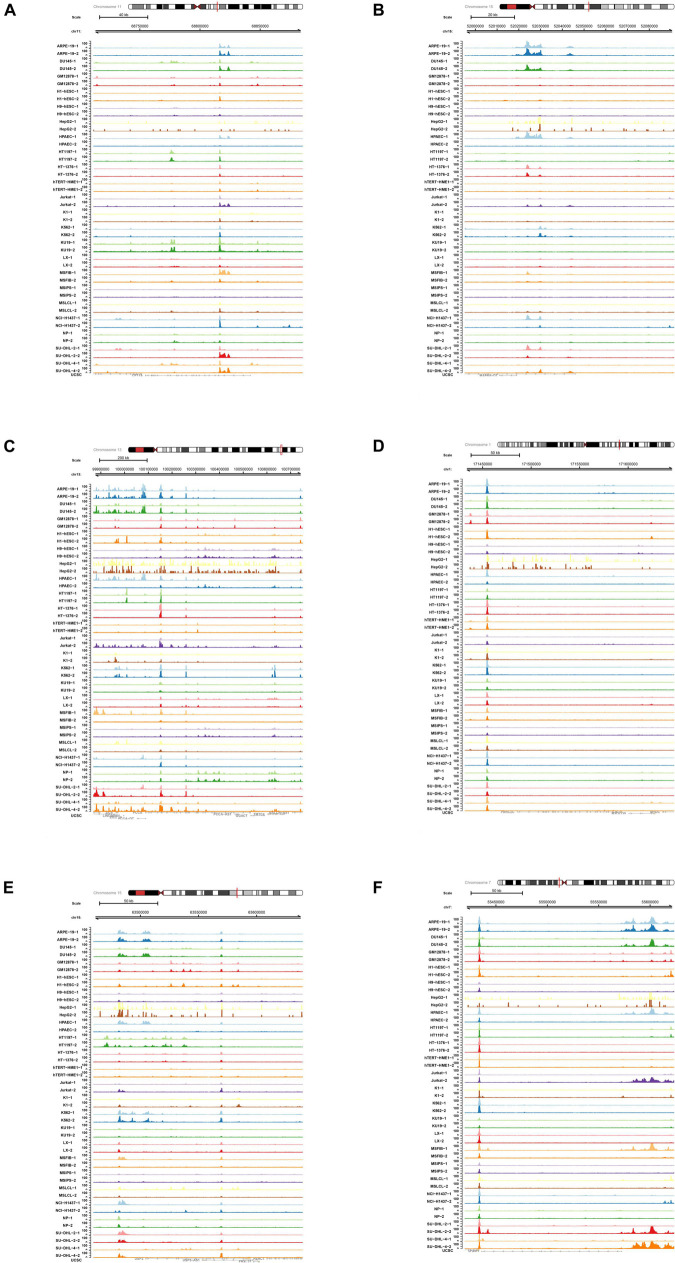
Chromatin immunoprecipitation sequence (ChIP-seq) analysis of key differentially expressed enhancer RNAs (DEeRNAs). **(A)** In ChIP-seq data of H3K27ac, multiple binding peaks were found in CPT1A sequences. **(B)** In ChIP-seq data of H3K27ac, multiple binding peaks were found in MAPK6 sequences. **(C)** In ChIP-seq data of H3K27ac, multiple binding peaks were found in PCCA sequences. **(D)** In ChIP-seq data of H3K27ac, multiple binding peaks were found in PRRC2C sequences. **(E)** In ChIP-seq data of H3K27ac, multiple binding peaks were found in USP3 sequences. **(F)** In ChIP-seq data of H3K27ac, multiple binding peaks were found in vesicular overexpressed in cancer prosurvival protein 1 (VOPP1) sequences.

In order to explore the role of enhancer-specific histone in modifications of eRNA transcription, ChIP-seq data of H3727ac were obtained and analyzed. The UCSC Genome Browser tracks illustrated enrichment of H3K27ac on multiple loci in various key eRNAs identified in this study (CPT1A, MAPK6, PCCA, PRRC2C, USP3, and VOPP1) (Figure 9).

Regulatory relationships between VOPP1 and other key markers (SFPQ and H3K27) were further explored using ChIP-seq and ATAC-seq methods. We retrieved relative data from the Cistrome database to detect DNA fragments binding with SFPQ and H3K27ac in various SCI-related and inflammation-related cases. The results showed that in ChIP-seq data of SFPQ (Supplementary Figure 1) and H3K27 (Supplementary Figure 2), there were strong binding peaks in the position of VOPP1, which basically coincided with the open chromosome region shown in the ATAC-seq results (Supplementary Figure 3). Furthermore, we downloaded the original ChIP-seq data of SFPQ and H3K2 to analyze the results with UCSC Genome Browser. Binding peaks in SFPQ and H3K27 groups were obviously stronger than those in control groups in the chromosomal position of VOPP1, further validating the binding relationship.

### Online Single-Cell RNA Sequencing Validation

To explore the cell subtype localization of the key eRNAs in the speculative regulation mechanisms, transcriptome combined with scRNA-seq data of SCI samples was analyzed. Sixteen clusters were identified using t-distributed stochastic neighbor embedding (t-SNE) (Supplementary Figure 4A). The heat map plot showed the top 5 marker genes of each cluster in SCI samples (Supplementary Figure 4B). The feature plots illustrated the distribution and expression level of SFPQ (Supplementary Figure 4C), VOPP1 (Supplementary Figure 4D), and EGFR (Supplementary Figure 4E) in SCI tissues.

## Discussion

Multiple pathogenic factors contribute to the damage of the spinal cord and lead to SCI, which is a common orthopedic disease (Phillips et al., 2015). SCI is a pathological process with multi-gene mutation and multi-pathway dysfunction that causes serious neurological dysfunction (Forner et al., 2016). Primary injuries of SCI during the early stages are usually accompanied by secondary ischemia, tissue edema, and ischemia–reperfusion injury. Because of the non-renewable features of nerve cells and extremely high deformity rates, SCI has brought economic and psychological burdens (Smith et al., 2015). Research reported that primary damages induced by early stage of SCI caused massive neuronal apoptosis and keratinocyte regeneration. Moreover, formation of glial scars also inhibited nerve fiber growth, as well as exogenous and endogenous factors (Selvarajah et al., 2015). Currently, studies of SCI are mainly concentrated in mice, whereas research on gene expression changes in peripheral whole blood is relatively inadequate (Jin et al., 2015; Ropper et al., 2015).

In this study, on the basis of comprehensive bioinformatics analysis, DETFs, SCI-related eRNAs, and target genes were all identified. Furthermore, according to 27 SCI-related DEeRNAs and significant DETF links, link of SFPQ (TF) and VOPP1 (eRNA) was extracted (*R* = 0.990, *p* < 0.001, positive). Based on eight regulation relationships of key DEeRNAs and DETGs, the link of VOPP1 (eRNA) and EGFR (target gene) was extracted (*R* = 0.974, *p* < 0.001, positive). And by co-analyzing VOPP1 and 22 types of immune cells or immune-related pathways, Th cells (*R* = −0.987, *p* < 0.001, negative) were postulated to be the most significant immune cells. On the basis of comprehensive consideration of GSVA and correlation results of VOPP1 and 50 hallmark gene sets, VOPP1 and hallmark coagulation (*R* = 0.937, *p* < 0.001, positive) showed the strongest interaction. Eventually, positively regulated by SFPQ, VOPP1 strengthened the transient expression of EGFR. Th cells and hallmark coagulation were the downstream immune cells/pathways of VOPP1 in SCI.

Neurocytes with enhanced expression of SFPQ and EGFR signal the lymphocytes overexpressing VOPP1 to infiltrate peripheral blood, which may play important roles in apoptosis and promote the development of inflammation in patients with SCI based on scRNA-seq and cellular communication analysis. In addition, based on ChIP-seq and ATAC-seq analysis, multiple binding peaks in SFPQ and H3K27 groups were identified in the chromosomal position of VOPP1, further validating the binding relationship.

VOPP1, also known as glioblastoma-amplified secreted protein (Dunham et al., 2012) and EGFR-coamplified and overexpressed protein (ECOP) (Fang et al., 2020), is upregulated in multiple human tumors, such as gastric cancer and squamous cell carcinoma (Baras et al., 2011; Gao et al., 2015). Overwhelming evidence demonstrates that VOPP1 acts as a critical regulator of nuclear factor kappa B (NF-κB) signaling pathway, and it could be implicated in apoptosis resistance (Li et al., 2017). The nuclear factor of kappa light polypeptide gene enhancer in B-cells inhibitor alpha (IκBα) is correlated with NF-κB that prevents NF-κB from binding cognate promoters and also leads to steady-state cytoplasm localization. When stimulated by activation signals including tumor necrosis factor alpha (TNFα), IκBα is quickly degraded by phosphorylation-dependent ubiquitination (Karin and Ben-Neriah, 2000). Degradation of IκBα causes NF-κB to translocate to the nucleus and leads to the activation of multiple genes that are significant in various processes, including apoptosis, immune-related and inflammation response, and cellular proliferation (Park and James, 2005). Inhibition of VOPP1 expression in PBMCs alleviated the inflammatory response in advanced sepsis patients (Li et al., 2018). Taken together, increased VOPP1 expression could confer resistance to apoptosis and promote the development of inflammation. Further study of VOPP1 in SCI pathological process may bring insights into refining therapeutic regimens, as well as other disease states that are associated with elevated VOPP1 activity, such as asthma, arthritis, chronic/acute inflammation, and diverse tumors (Karin et al., 2002).

SFPQ refers to a multifunctional protein, which contributes to substantial biological processes, including DNA synthesis, gene expression regulation, DNA repair, RNA splicing, and apoptosis (Shav-Tal and Zipori, 2002). Importantly, SFPQ plays a critical role in neuronal differentiation and survival (Lowery et al., 2007). Furthermore, central nervous system (CNS) injury stimulates SFPQ expression, hindering neurogenesis and nerve regeneration, which may inhibit the repair of injured spinal cord (Elsaeidi et al., 2014). SFPQ is rich in paraspeckles, RNA-protein structures identified in the interchromatin space of nucleus, which increase in response to pro-inflammatory stimuli (Fox and Lamond, 2010). Additionally, SFPQ were significantly upregulated in chronic inflammatory diseases, including Crohn’s disease and ulcerative colitis (Hasler et al., 2011). Hence, this provides novel options for researchers in the field of SCI because SFPQ not only shows SCI-specific effects of inflammation that serves as a diagnostic biomarker but also can be a potential target for therapeutic intervention of SCI.

Based on integrated multinomial bioinformatics analysis and other studies, EGFR was suggested to be a target gene of VOPP1 (Wang et al., 1998; Eley et al., 2002). EGFR belongs to the receptor tyrosine kinase (RTK) superfamily, consisting of three other members, ErbB2/Neu/HER-2, ErbB3/HER-3, and ErbB4/HER-4 (Romano and Bucci, 2020). Being a neurotrophin receptor to initiate cellular signaling and regulate neuronal processes, EGFR has many important roles in the CNS (Hennigan et al., 2007). Moreover, EGFR is important in neural stem cells self-renewal, and loss of EGFR signaling induces these cells to differentiate preferentially into glia, which may promote glia scar formation in SCI and influence functional recovery (Robson et al., 2018). Furthermore, EGFR is crucial in regulating the differentiation of precursors to astrocytes, and increased EGFR expression levels determine their differentiation to astrocytes (Burrows et al., 1997). EGFR also plays an important role in astrocytes’ morphology. Blockade of its expression causes disorganization of astrocytes in CNS development, losing their processes surrounding neurons, which induces degeneration of abundant axons of nerve (Liu and Neufeld, 2007). Modulation of EGFR expression may be propitious to activate regeneration and counteract neurodegeneration (Ceresoli, 2012). Therefore, EGFR is critical for differentiation, growth, and repair of the injured tissue in the spinal cord, which could be a potential target for therapy of patients with SCI.

Immune response in peripheral blood plays an important role in maintaining spinal cord homeostasis. Dysfunction or disorder caused by SCI in vegetative innervation of lymphatic and endocrine systems leads to long-term abnormal inflammation responses (Zhang Y. et al., 2013). Differentiating to functionally distinct Th subsets, Th cells are crucial in normal immune surveillance and proper immune regulation (Murphy and Reiner, 2002). Importantly, SCI is associated with immune depression syndrome, owing to the dysregulated hypothalamic–pituitary–adrenal (HPA) axis and dysfunctioned sympathetic nervous system (Jones, 2014). Based on bioinformatics analysis and other studies, a rapid decrease in Th cells was identified in patients with SCI, contributing to immunosuppression in the acute phase of SCI (Gao et al., 2021). We postulated that overexpression of VOPP1 decreased the counts of Th cells in peripheral blood of SCI patients. It is an important reason that acute SCI patient becoming chronic, revealing novel targets for future SCI immunotherapy.

SCI is correlated with a remarkable thrombophilia and aberrant coagulation. In addition, the relation between deep venous thrombosis (DVT) and SCI has been well established (Yao, 1992). Numerous experimental research with fibrinogen scanning has shown the presence of DVT in nearly 100% of patients with acute SCI (Maynard et al., 1997). Sequelae from SCI affect the three components of Virchow triad, which are responsible for DVT, blood flow deceleration, vascular endothelial damage, release of procoagulants, and coagulation cascade activation, resulting in hypercoagulability state. Furthermore, patients with SCI often show dehydration and secondary increase of blood viscosity, aggravating the stasis and hypoxia in injured spinal cord (Ersoz et al., 1999). On the basis of bioinformatics analysis and other studies, coagulation was postulated to contribute to the pathogenesis and progress of SCI *via* the positive regulation of VOPP1, which is a promising entry point of therapeutic interventions for SCI (Ghlissi et al., 2019).

TSA refers to a highly specific hydroxamic acid that can inhibit histone deacetylase (HDAC) enzymes. Importantly, HDACs repress repressor element 1 silencing transcription factor (REST) that is able to counteract neuronal differentiation traits and Sp1, a TF that can mediate neuronal antioxidant signaling pathways. TSA leads to histone hyperacetylation, accompanied by activation of antioxidant gene expression and neuronal maturation, which indicate TF derepression of REST and Sp1 (Hakimi et al., 2002; Ryu et al., 2003). Moreover, TSA can block immune cell proliferation and suppress pro-Th1 factor IFN-γ, which could cause the transformation of Th1 to Th2 phenotype, indicating neuroimmunoprotective effects (Dangond et al., 2004). Importantly, TSA can also inhibit CNS immune cell infiltration and lipid breakdown products absorbing of microglial cells, which are consistent with anti-microglial activation of TSA recently reported (Konishi et al., 2002). Furthermore, a recent treatment showed TSA was able to decrease nitrosylation of spinal cord tissues, protecting nerve cells from free radical attack after SCI (Dasgupta et al., 2003). TSA increases histone acetylation and E2F-dependent transcripts in injured spinal cords, indicating its significant neuroprotective effect. Taken together, the general effect of TSA is to adjust dysregulated homeostatic processes, promoting histone acetylation and harnessing much more favorable genes than pathogenicity gene such as chemokine and pro-Th1, which may be effective for SCI treatment.

There were still several limitations to this study. First, the data were obtained from public sources statistically imperfect with limited samples. Second, information on other confounding variables in this study, such as smoking, was not available. Third, a prospective study is needed to evaluate the significance of these key biomarkers in terms of long-term clinical outcomes and possible applications of molecular drugs for SCI therapy. Finally, further experiment is an absolute necessity in demonstrating the regulatory mechanisms of key eRNAs implicated in SCI. Therefore, ChIP-seq data of H3K27ac from online databases were obtained and analyzed, which broadened the scope of validation and supplemented the specific regulatory mechanisms of eRNA action involved in the pathogenesis of SCI. ATAC-seq data of VOPP1 (key eRNA) were also utilized to validate the eRNA regulatory mechanisms. Moreover, the cell subtype localizations of the key eRNAs and TFs were identified by scRNA-seq validation fluorescence immunohistochemistry. Additionally, a comprehensive transcriptome bioinformatics analysis of spatial transcriptome and scRNA-seq, fluorescence immunohistochemistry, and eRNA-related direct mechanism experiments would be the further research directions.

## Conclusion

Based on integrated multinomial bioinformatics analysis, we found that SFPQ was the most significant TF and VOPP1 was the most significant key eRNA in the progression of SCI patients. In addition, during this pathological process, VOPP1 upregulated the transient expression of EGFR. Th cells and hallmark coagulation were the potential downstream pathways of VOPP1. Moreover, this study provided candidate small-molecule compounds as potential targets for the treatment of SCI patients.

## Data Availability Statement

Publicly available datasets were analyzed in this study. This data can be found here: Gene Expression Omnibus (GEO, https://www.ncbi.nlm.nih.gov/geo/), ArrayExpress (https://www.ebi.ac.uk/arrayexpress/), and Cistrome Cancer database (http://cistrome.org/).

## Author Contributions

RH, SW, RZ, SX, ZH, LC, and JZ: conception and design, collection and assembly of data, data analysis and interpretation, the manuscript writing, and final approval of the manuscript. All authors contributed to the article and approved the submitted version.

## Conflict of Interest

The authors declare that the research was conducted in the absence of any commercial or financial relationships that could be construed as a potential conflict of interest.

## Publisher’s Note

All claims expressed in this article are solely those of the authors and do not necessarily represent those of their affiliated organizations, or those of the publisher, the editors and the reviewers. Any product that may be evaluated in this article, or claim that may be made by its manufacturer, is not guaranteed or endorsed by the publisher.

## Footnotes

1
https://www.ncbi.nlm.nih.gov/geo/

2
https://www.ebi.ac.uk/arrayexpress/

3
https://www.ncbi.nlm.nih.gov/geo/query/acc.cgi?acc=GPL21975

4
http://cistrome.org/

5
http://www.immport.org/

6
http://software.broadinstitute.org/gsea/msigdb/

7
https://cibersort.stanford.edu/

8
https://portals.broadinstitute.org/cmap/

9
http://clue.io/

10
https://www.10xgenomics.com/instruments/chromium-x-series

11
http://10xgenomics.com/

12
http://biocc.hrbmu.edu.cn/CellMarker/

13
https://github.com/Coolgenome/iTALK

14
http://genome.ucsc.edu

15
https://github.com/kassambara/ggpubr

16
https://liulab-dfci.github.io/

17
http://younglab.wi.mit.edu/super_enhancer_code.html

18
https://www.ebi.ac.uk/gxa/sc/experiments/E-MTAB-7320/results/tsne

19
www.r-project.org

## Supplementary Material

Supplementary Figure 1 In SFPQ Chromatin immunoprecipitation sequence (ChIP-seq) data, multiple binding peaks were found in VOPP1 sequences.

Supplementary Figure 2 In H3K27 Chromatin immunoprecipitation sequence (ChIP-seq) data, multiple binding peaks were identified in VOPP1 sequences.

Supplementary Figure 3 In SFPQ Assay for Transposase-Accessible Chromatin with high-throughput sequencing (ATAC-seq) data, multiple binding peaks were identified in VOPP1 sequences.

Supplementary Figure 4 Single-cell RNA-seq validation. **(A)** A total of 25 numbered clusters were identified by t-NSE. **(B)** Feature plots illustrating the distribution and expression of SFPQ. **(C)** Feature plots illustrating the distribution and expression of VOPP1. **(D)** Feature plots illustrating the distribution and expression of EGFR. **(E)** Heat map showing the expression level of the top 5 marker genes of each cluster.

Supplementary Table 1 Baseline information of 27 normal peripheral blood mononuclear cell (PBMC) samples and 25 spinal cord injury PBMC samples.
