# Supplementary figures and images for: Identification of Key eRNAs for Spinal Cord Injury by Integrated Multinomial Bioinformatics Analysis
Source: Front Cell Dev Biol. 2021 Oct 11;9:728242. doi: 10.3389/fcell.2021.728242 (PMC8542800; doi:10.3389/fcell.2021.728242)

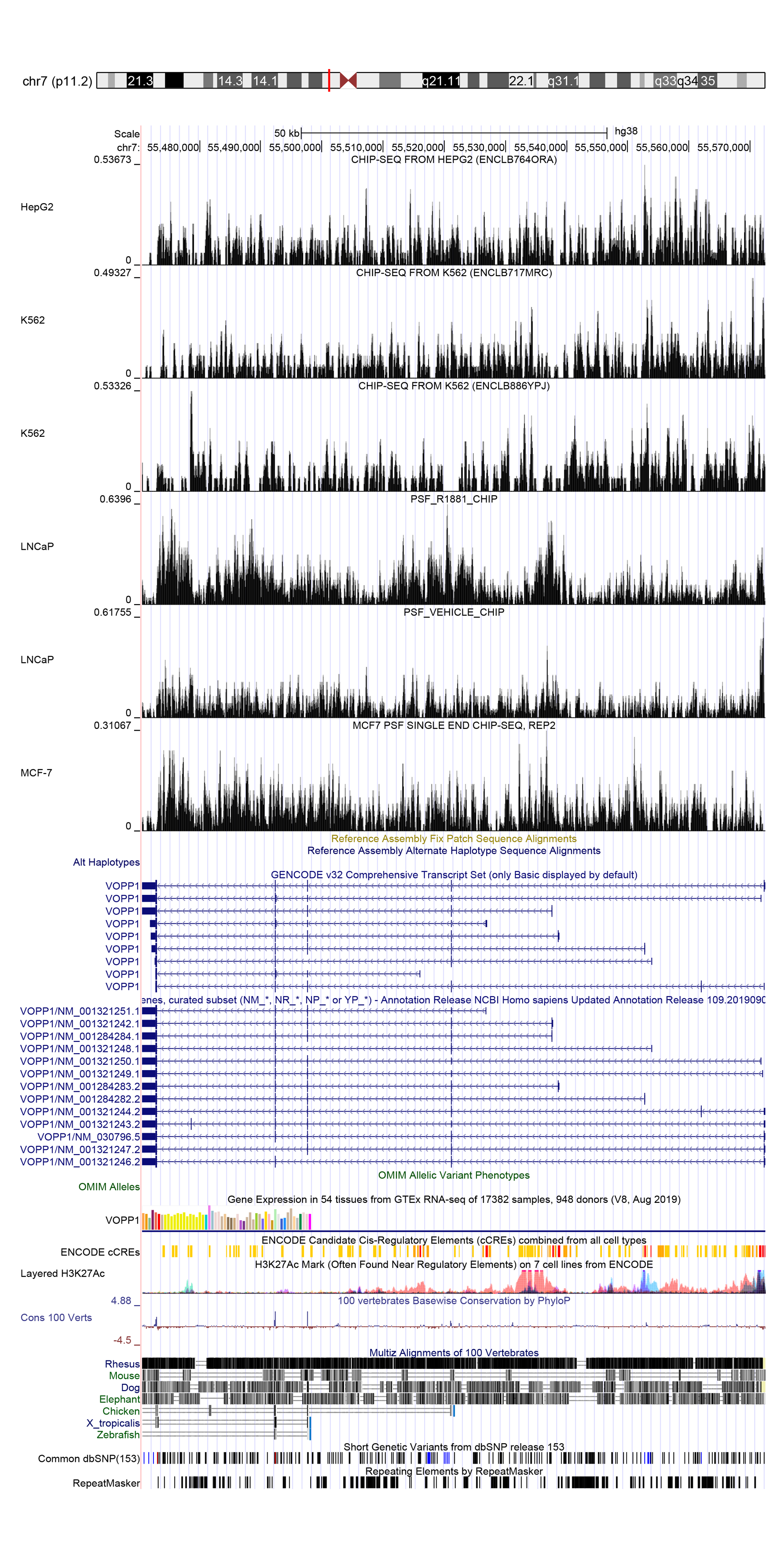

Supplement: Supplementary Figure 1 — In SFPQ Chromatin immunoprecipitation sequence (ChIP-seq) data, multiple binding peaks were found in VOPP1 sequences. [file Image_1.TIF]

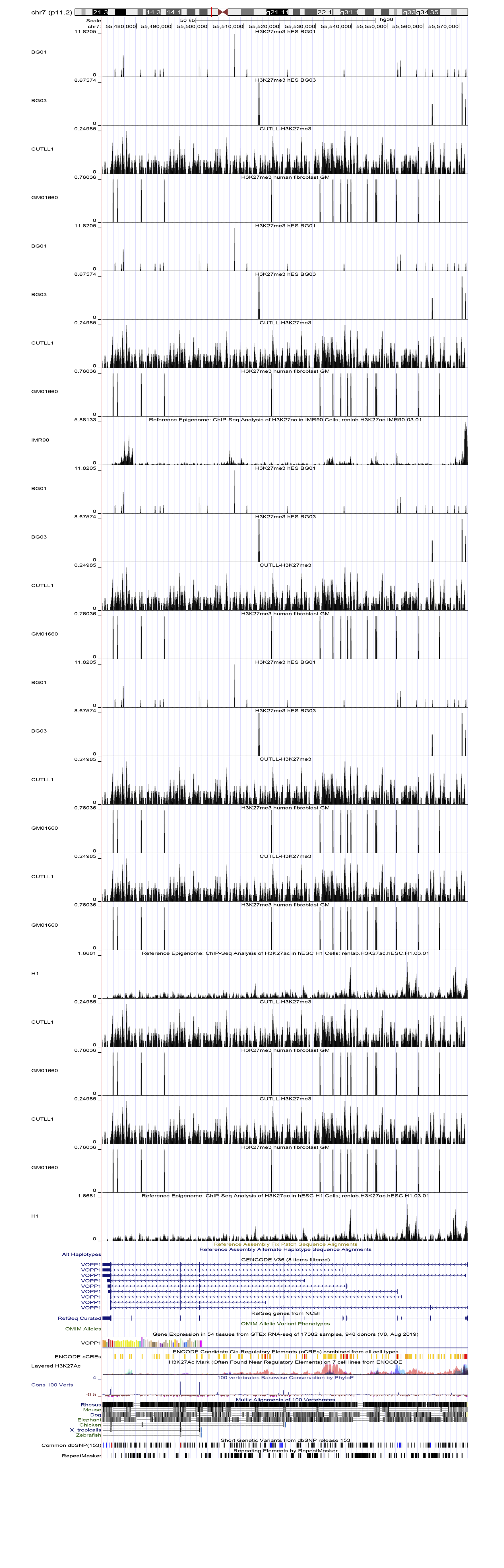

Supplement: Supplementary Figure 2 — In H3K27 Chromatin immunoprecipitation sequence (ChIP-seq) data, multiple binding peaks were identified in VOPP1 sequences. [file Image_2.TIF]

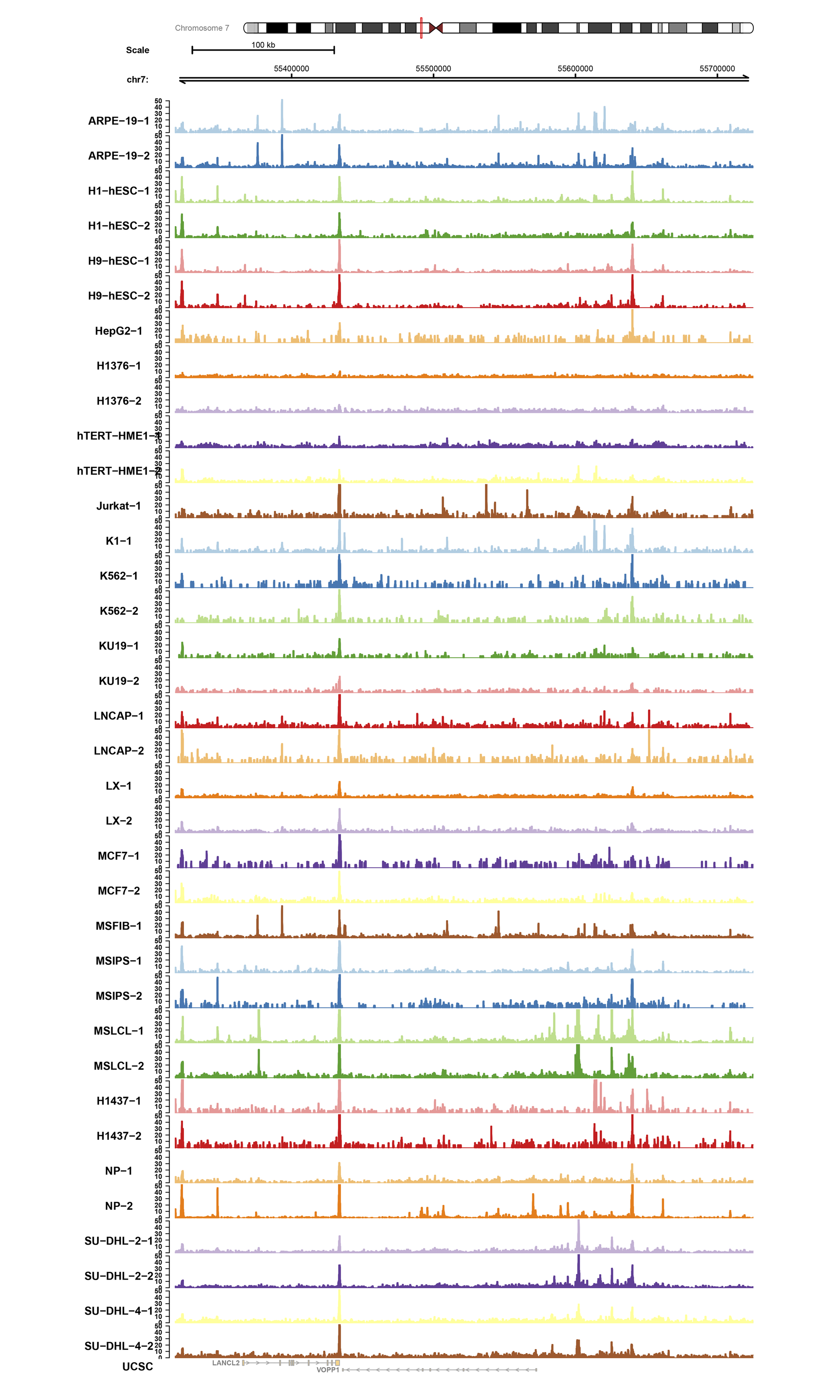

Supplement: Supplementary Figure 3 — In SFPQ Assay for Transposase-Accessible Chromatin with high-throughput sequencing (ATAC-seq) data, multiple binding peaks were identified in VOPP1 sequences. [file Image_3.TIF]

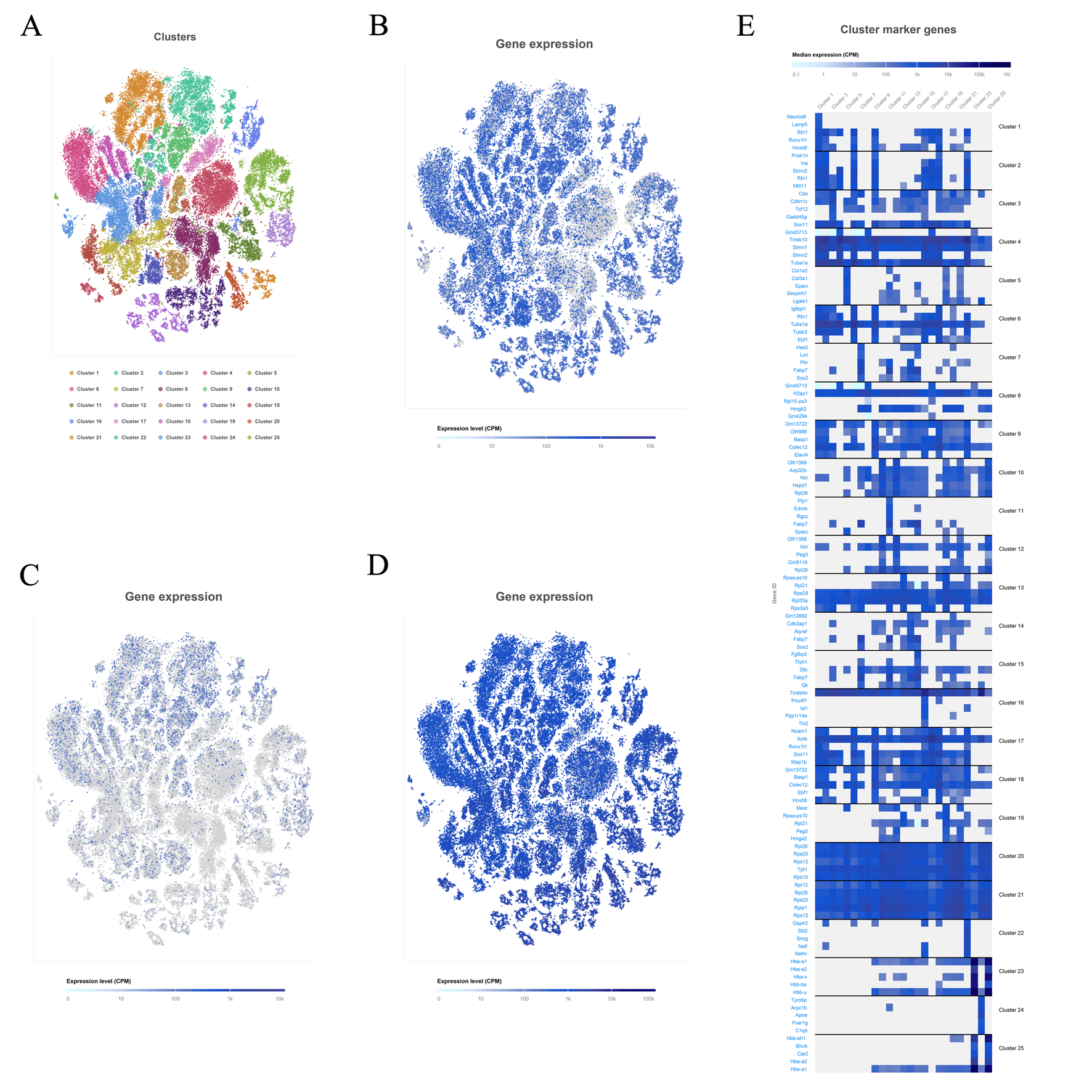

Supplement: Supplementary Figure 4 — Single-cell RNA-seq validation. (A) A total of 25 numbered clusters were identified by t-NSE. (B) Feature plots illustrating the distribution and expression of SFPQ. (C) Feature plots illustrating the distribution and expression of VOPP1. (D) Feature plots illustrating the distribution and expression of EGFR. (E) Heat map showing the expression level of the top 5 marker genes of each cluster. [file Image_4.TIF]
