# Supplementary material for: Identification of Key eRNAs for Spinal Cord Injury by Integrated Multinomial Bioinformatics Analysis
Source: Front Cell Dev Biol. 2021 Oct 11;9:728242. doi: 10.3389/fcell.2021.728242 (PMC8542800; doi:10.3389/fcell.2021.728242)
Supplement: Supplementary Table 1 — Baseline information of 27 normal peripheral blood mononuclear cell (PBMC) samples and 25 spinal cord injury PBMC samples. [file Table_1.docx]

Table S1: Baseline information of 27 normal peripheral blood mononuclear cell (PBMC) samples and 25 spinal cord injury PBMC samples

| ID | Group | Pain | Batch | Platform |
| --- | --- | --- | --- | --- |
| CTRL_01 | normal whole blood | normal | GSE82152 | GPL21975 |
| CTRL_02 | normal whole blood | normal | GSE82152 | GPL21975 |
| CTRL_03 | normal whole blood | normal | GSE82152 | GPL21975 |
| CTRL_04 | normal whole blood | normal | GSE82152 | GPL21975 |
| CTRL_05 | normal whole blood | normal | GSE82152 | GPL21975 |
| CTRL_06 | normal whole blood | normal | GSE82152 | GPL21975 |
| CTRL_07 | normal whole blood | normal | GSE82152 | GPL21975 |
| CTRL_08 | normal whole blood | normal | GSE82152 | GPL21975 |
| CTRL_09 | normal whole blood | normal | GSE82152 | GPL21975 |
| CTRL_10 | normal whole blood | normal | GSE82152 | GPL21975 |
| CTRL_11 | normal whole blood | normal | GSE82152 | GPL21975 |
| CTRL_12 | normal whole blood | normal | GSE82152 | GPL21975 |
| CTRL_13 | normal whole blood | normal | GSE82152 | GPL21975 |
| CTRL_14 | normal whole blood | normal | GSE82152 | GPL21975 |
| CTRL_15 | normal whole blood | normal | GSE82152 | GPL21975 |
| CTRL_16 | normal whole blood | normal | GSE82152 | GPL21975 |
| CTRL_17 | normal whole blood | normal | GSE82152 | GPL21975 |
| CTRL_18 | normal whole blood | normal | GSE82152 | GPL21975 |
| CTRL_19 | normal whole blood | normal | GSE82152 | GPL21975 |
| CTRL_20 | normal whole blood | normal | GSE82152 | GPL21975 |
| CTRL_21 | normal whole blood | normal | GSE82152 | GPL21975 |
| CTRL_22 | normal whole blood | normal | GSE82152 | GPL21975 |
| CTRL_23 | normal whole blood | normal | GSE82152 | GPL21975 |
| CTRL_24 | normal whole blood | normal | GSE82152 | GPL21975 |
| CTRL_25 | normal whole blood | normal | GSE82152 | GPL21975 |
| CTRL_26 | normal whole blood | normal | GSE82152 | GPL21975 |
| CTRL_27 | normal whole blood | normal | GSE82152 | GPL21975 |
| GSM1712184_GATA_BY_C2_PrimeView | spinal cord injury blood | no pain | E-GEOD-69901 | GPL21975 |
| GSM1712185_GATA_BY_C8_PrimeView | spinal cord injury blood | no pain | E-GEOD-69901 | GPL21975 |
| GSM1712186_GATA_BY_C11_PrimeView | spinal cord injury blood | no pain | E-GEOD-69901 | GPL21975 |
| GSM1712187_GATA_BY_C12_PrimeView | spinal cord injury blood | no pain | E-GEOD-69901 | GPL21975 |
| GSM1712188_GATA_BY_C13_PrimeView | spinal cord injury blood | no pain | E-GEOD-69901 | GPL21975 |
| GSM1712189_GATA_BY_C14_PrimeView | spinal cord injury blood | no pain | E-GEOD-69901 | GPL21975 |
| GSM1712190_GATA_BY_C15_PrimeView | spinal cord injury blood | no pain | E-GEOD-69901 | GPL21975 |
| GSM1712191_GATA_BY_C16_PrimeView | spinal cord injury blood | no pain | E-GEOD-69901 | GPL21975 |
| GSM1712192_GATA_BY_C17_PrimeView | spinal cord injury blood | no pain | E-GEOD-69901 | GPL21975 |
| GSM1712193_GATA_BY_C18_PrimeView | spinal cord injury blood | no pain | E-GEOD-69901 | GPL21975 |
| GSM1712194_GATA_BY_C19_PrimeView | spinal cord injury blood | no pain | E-GEOD-69901 | GPL21975 |
| GSM1712195_GATA_BY_C23_PrimeView | spinal cord injury blood | no pain | E-GEOD-69901 | GPL21975 |
| GSM1712196_GATA_BY_C24_PrimeView | spinal cord injury blood | no pain | E-GEOD-69901 | GPL21975 |
| GSM1712172_GATA-1_PrimeView | spinal cord injury blood | intractable neuropathic pain | E-GEOD-69901 | GPL21975 |
| GSM1712173_GATA_BY_A3_PrimeView | spinal cord injury blood | intractable neuropathic pain | E-GEOD-69901 | GPL21975 |
| GSM1712174_GATA_BY_A4_PrimeView | spinal cord injury blood | intractable neuropathic pain | E-GEOD-69901 | GPL21975 |
| GSM1712175_GATA_BY_A9_PrimeView | spinal cord injury blood | intractable neuropathic pain | E-GEOD-69901 | GPL21975 |
| GSM1712176_GATA_BY_A20_PrimeView | spinal cord injury blood | intractable neuropathic pain | E-GEOD-69901 | GPL21975 |
| GSM1712177_GATA_BY_A21_PrimeView | spinal cord injury blood | intractable neuropathic pain | E-GEOD-69901 | GPL21975 |
| GSM1712178_GATA_BY_A22_PrimeView | spinal cord injury blood | intractable neuropathic pain | E-GEOD-69901 | GPL21975 |
| GSM1712179_GATA_BY_A25_PrimeView | spinal cord injury blood | intractable neuropathic pain | E-GEOD-69901 | GPL21975 |
| GSM1712180_GATA_BY_A26_PrimeView | spinal cord injury blood | intractable neuropathic pain | E-GEOD-69901 | GPL21975 |
| GSM1712181_GATA_BY_A27_PrimeView | spinal cord injury blood | intractable neuropathic pain | E-GEOD-69901 | GPL21975 |
| GSM1712182_GATA_BY_A28_PrimeView | spinal cord injury blood | intractable neuropathic pain | E-GEOD-69901 | GPL21975 |
| GSM1712183_GATA_BY_A29_PrimeView | spinal cord injury blood | intractable neuropathic pain | E-GEOD-69901 | GPL21975 |
