# Supplementary material for: Identification of Key eRNAs for Spinal Cord Injury by Integrated Multinomial Bioinformatics Analysis
Source: Front Cell Dev Biol. 2021 Oct 11;9:728242. doi: 10.3389/fcell.2021.728242 (PMC8542800; doi:10.3389/fcell.2021.728242)
Supplement: Supplementary file 7 [file Data_Sheet_2.docx]

library(e1071)

library(parallel)

library(preprocessCore)

library(sva)

library(limma)

library(edgeR)

library(ggplot2)

library(survminer)

library(survival)

library(rms)

library(randomForest)

library(pROC)

library(glmnet)

library(pheatmap)

library(timeROC)

library(vioplot)

library(corrplot)

library(ConsensusClusterPlus)

library(forestplot)

library(survivalROC)

library(beeswarm)

library(edgeR)

library(chromVAR)

library(Biostrings)

library(BSgenome.Hsapiens.UCSC.hg38)

library(ChIPseeker)

library(TxDb.Hsapiens.UCSC.hg38.knownGene)

library(clusterProfiler)

library(org.Hs.eg.db)

library(ggplot2)

library(karyoploteR)

library(limma)

library(pheatmap)

library(GSVA)

library(limma)

library(GSEABase)

library(stringr)

library(GEOquery)

library(dplyr)

library(limma)

library(ComplexHeatmap)

library(RColorBrewer)

library(clusterProfiler)

library(tibble)

library(ggplot2)

library(cowplot)

library(ggcorrplot)

library(xlsx)

library(tidyverse)

library(GEOquery)

library(plyr)

library(circlize)

library(ComplexHeatmap)

library(TCGAbiolinks)

library(SummarizedExperiment)

library(dplyr)

library(tidyverse)

library(fgsea)

library(ggplot2)

library(ImmuLncRNA)

setwd("J:\\eRNA数据\\脊髓损伤外周血")

nonmetastasis = 27

metastasis = 25

logFoldChange=1.2

adjustP=0.05

library(limma)

rt=read.table("symbol_TPM.txt",sep="\t",header=T,check.names=F)

rt=as.matrix(rt)

rownames(rt)=rt[,1]

exp=rt[,2:ncol(rt)]

dimnames=list(rownames(exp),colnames(exp))

rt=matrix(as.numeric(as.matrix(exp)),nrow=nrow(exp),dimnames=dimnames)

rt=avereps(rt)

rt= na.omit(rt)

#differential

type=c(rep("con",nonmetastasis),rep("treat",metastasis))

design <- model.matrix(~0+factor(type))

colnames(design) <- c("con","treat")

fit <- lmFit(rt,design)

cont.matrix<-makeContrasts(treat-con,levels=design)

fit2 <- contrasts.fit(fit, cont.matrix)

fit2 <- eBayes(fit2)

allDiff=topTable(fit2,adjust='fdr',number=200000)

allOut=rbind(id=colnames(allDiff),allDiff)

write.table(allOut,file="limmaTab.xls",sep="\t",quote=F,col.names=F)

#write table

diffSig <- allDiff[with(allDiff, (abs(logFC)>logFoldChange & adj.P.Val < adjustP )), ]

diffOut=rbind(id=colnames(diffSig),diffSig)

write.table(diffOut,file="diff.xls",sep="\t",quote=F,col.names=F)

#write expression level of diff gene

hmExp=rt[which(rownames(rt)%in%rownames(diffSig)),]

diffExp=rbind(id=colnames(hmExp),hmExp)

write.table(diffExp,file="diffExp.txt",sep="\t",quote=F,col.names=F)

colnames(allDiff)[5]= "FDR"

allDiff$FDR[allDiff$FDR<=0]=2.22329540628561e-322

#volcano

#plot_mode <- "classic" #经典版

plot_mode <- "advanced" #酷炫版

logFCcut <- 0.3 #log2-foldchange

pvalCut <- 0.05 #P.value

adjPcut <- 0.05 #adj.P.value

#for advanced mode

logFCcut2 <- 1.5

logFCcut3 <- 3

pvalCut2 <- 0.0001

pvalCut3 <- 0.00001

#置x，y軸的最大最小位置

xmin <- (range(allDiff$logFC)[1]- (range(allDiff$logFC)[1]+ 15))

xmax <- (range(allDiff$logFC)[1]+ (15-range(allDiff$logFC)[1]))

ymin <- 0

ymax <- 85

# 基因名的颜色，需大于等于pathway的数量，这里自定义了足够多的颜色

mycol <- c("darkgreen","chocolate4","blueviolet","#223D6C","#D20A13","#088247","#58CDD9","#7A142C","#5D90BA","#431A3D","#91612D","#6E568C","#E0367A","#D8D155","#64495D","#7CC767")

###########plot

if (plot_mode == "classic"){

# 簡單的setting for color

allDiff$color_transparent <- ifelse((allDiff$FDR < pvalCut & allDiff$logFC > logFCcut), "red", ifelse((allDiff$FDR < pvalCut & allDiff$logFC < -logFCcut), "blue","grey"))

# 簡單的setting for size

size <- ifelse((allDiff$FDR < pvalCut & abs(allDiff$logFC) > logFCcut), 4, 2)

} else if (plot_mode == "advanced") {

# 複雜的的setting for color

n1 <- length(allDiff[, 1])

cols <- rep("grey", n1)

names(cols)<- rownames(allDiff)

#不同阈值的点的颜色

cols[allDiff$FDR < pvalCut & allDiff$logFC >logFCcut]<- "#FB9A99"

cols[allDiff$FDR < pvalCut2 & allDiff$logFC > logFCcut2]<- "#ED4F4F"

cols[allDiff$FDR < pvalCut & allDiff$logFC < -logFCcut]<- "#B2DF8A"

cols[allDiff$FDR < pvalCut2 & allDiff$logFC < -logFCcut2]<- "#329E3F"

color_transparent <- adjustcolor(cols, alpha.f = 0.5)

allDiff$color_transparent <- color_transparent

# 複雜的的setting for size

n1 <- length(allDiff[, 1])

size <- rep(1, n1)

#不同阈值的点的大小

size[allDiff$FDR < pvalCut & allDiff$logFC > logFCcut]<- 2

size[allDiff$FDR < pvalCut2 & allDiff$logFC > logFCcut2]<- 4

size[allDiff$FDR < pvalCut3 & allDiff$logFC > logFCcut3]<- 6

size[allDiff$FDR < pvalCut & allDiff$logFC < -logFCcut]<- 2

size[allDiff$FDR < pvalCut2 & allDiff$logFC < -logFCcut2]<- 4

size[allDiff$FDR < pvalCut3 & allDiff$logFC < -logFCcut3]<- 6

} else {

stop("Unsupport mode")

}

# Construct the plot object

p1 <- ggplot(data=allDiff, aes(logFC, -log10(FDR))) +

geom_point(alpha = 0.6, size = size, colour = allDiff$color_transparent) +

labs(allDiff=bquote(~Log[2]~"(fold change)"), y=bquote(~-Log[10]~italic("P-value")), title="") +

ylim(c(ymin,ymax)) +

scale_x_continuous(

breaks = c(-10, -5, -logFCcut, 0, logFCcut, 5, 10), #刻度线的位置

labels = c(-10, -5, -logFCcut, 0, logFCcut, 5, 10),

limits = c(-11, 11) #x轴范围，两侧对称才好看

) +

#或用下面这行：

xlim(c(xmin, xmax)) +

#画阈值分界线

geom_vline(xintercept = c(-logFCcut, logFCcut), color="grey40",

linetype="longdash", lwd = 0.5) + #虚线的形状和粗细

geom_hline(yintercept = -log10(pvalCut), color="grey40",

linetype="longdash", lwd = 0.5) +

theme_bw(base_size = 12#, base_family = "Times" #修改字体

) +

theme(panel.grid=element_blank())

if (plot_mode == "advanced") {

p1 <- p1 +

geom_vline(xintercept = c(-logFCcut2, logFCcut2), color="grey40",

linetype="longdash", lwd = 0.5) +

geom_hline(yintercept = -log10(pvalCut2), color="grey40",

linetype="longdash", lwd = 0.5)

}

pdf(file="vol.pdf")

p1

dev.off()

#heatmap

#Type=c(rep("primarytumor",nonmetastasis),rep("new_tumor_event",metastasis))

#Type=read.table("clinical_initial.txt",sep="\t",header=T,check.names=F,row.names = 1)

#Type=c(rep("normal",nonmetastasis),rep("tumor",metastasis))

#Type=c(rep("non-extranodal_involvement",nonmetastasis),rep("extranodal_involvement",metastasis))

#Type=c(rep("low_mRNAsi",nonmetastasis),rep("high_mRNAsi",metastasis))

#Type=c(rep("non-metastasis",nonmetastasis),rep("metastasis",metastasis))

#Type=c(rep("normal whole blood",27),rep("spinal cord injury blood",25))

#Type=c(rep("Primary tumor",nonmetastasis),rep("Bone metastatic tumor",metastasis))

#Type=c(rep("Normal_bone",nonmetastasis),rep("Chordoma",metastasis))

#Type=c(rep("Skeletal_muscle",nonmetastasis),rep("Rhabdomyosarcoma",metastasis))

#Type=c(rep("Normal_bone",nonmetastasis),rep("Ewing_sarcoma",metastasis))

Type=c(rep("Normal blood",nonmetastasis),rep("SCI blood",metastasis))

names(Type)=colnames(hmExp)

Type=as.data.frame(Type)

pdf(file="heatmap_initial.pdf",height=6,width=10)

pheatmap(hmExp,

annotation=Type,

color = colorRampPalette(c("#3F007D", "white", "#E34A33"))(50),

cluster_cols =F,

show_colnames = F,

show_rownames = F,

fontsize = 12,

fontsize_row=3,

fontsize_col=10)

dev.off()

###colorRampPalette(c("green", "white", "red"))(50)

######colorRampPalette(c("green", "black", "red"))(50)

######bk <- c(seq(-9,-0.1,by=0.01),seq(0,9,by=0.01))

######c(colorRampPalette(colors = c("blue","white"))(length(bk)/2),colorRampPalette(colors = c("white","red"))(length(bk)/2)),

library(chromVAR)

library(Biostrings)

library(BSgenome.Hsapiens.UCSC.hg38)

library(ChIPseeker)

library(TxDb.Hsapiens.UCSC.hg38.knownGene)

library(clusterProfiler)

library(org.Hs.eg.db)

library(ggplot2)

library(karyoploteR)

library(limma)

library(GOplot)

genes=as.vector(rownames(diffSig))

entrezIDs <- mget(genes, org.Hs.egSYMBOL2EG, ifnotfound=NA)

entrezIDs <- as.character(entrezIDs)

out=cbind(rownames(diffSig),diffSig[,1],entrezID=entrezIDs)

colnames(out)[1]="gene"

colnames(out)[2]="logFC"

write.table(out,file="id_DEG.txt",sep="\t",quote=F,row.names=F)

rt=read.table("id_DEG.txt",sep="\t",header=T,check.names=F)

rt=rt[is.na(rt[,"entrezID"])==F,]

gene=as.vector(rt$entrezID)

#GO

kk <- enrichGO(gene = gene,

OrgDb = org.Hs.eg.db,

pvalueCutoff =0.05,

qvalueCutoff = 0.05,

ont="all",

readable =T)

write.table(kk,file="GO_DEG.txt",sep="\t",quote=F,row.names = F)

pdf(file="GO_barplot_DEG.pdf",width = 12,height = 7)

barplot(kk, drop = TRUE, showCategory =10,split="ONTOLOGY") + facet_grid(ONTOLOGY~., scale='free')

dev.off()

pdf(file="GO_dotplot_DEG.pdf",width = 12,height = 7)

dotplot(kk,showCategory = 10,split="ONTOLOGY") + facet_grid(ONTOLOGY~., scale='free')

dev.off()

geneFC=2^rt$logFC

pdf(file="GO_circos_DEG.pdf",width = 12,height = 7)

cnet=cnetplot(kk, foldChange=geneFC, showCategory = 3, circular = TRUE, colorEdge = TRUE)

print(cnet)

dev.off()

#KEGG

genes=as.vector(rownames(diffSig))

entrezIDs <- mget(genes, org.Hs.egSYMBOL2EG, ifnotfound=NA)

entrezIDs <- as.character(entrezIDs)

out=cbind(rownames(diffSig),diffSig[,1],entrezID=entrezIDs)

colnames(out)[1]="gene"

colnames(out)[2]="logFC"

write.table(out,file="id_DEG.txt",sep="\t",quote=F,row.names=F)

rt=read.table("id_DEG.txt",sep="\t",header=T,check.names=F)

rt=rt[is.na(rt[,"entrezID"])==F,]

gene=rt$entrezID

geneFC=2^rt$logFC

names(geneFC)=gene

pvalueFilter=0.05

qvalueFilter=0.05

colorSel="qvalue"

if(qvalueFilter>0.05){

colorSel="pvalue"

}

kk <- enrichKEGG(gene = gene, organism = "hsa", pvalueCutoff =0.99, qvalueCutoff =0.99)

KEGG=as.data.frame(kk)

KEGG$geneID=as.character(sapply(KEGG$geneID,function(x)paste(rt$gene[match(strsplit(x,"/")[[1]],as.character(rt$entrezID))],collapse="/")))

KEGG=KEGG[(KEGG$pvalue<pvalueFilter & KEGG$qvalue<qvalueFilter),]

write.table(KEGG,file="KEGG_DEG.txt",sep="\t",quote=F,row.names = F)

showNum=30

if(nrow(KEGG)<showNum){

showNum=nrow(KEGG)

}

pdf(file="KEGG_barplot_DEG.pdf",width = 12,height = 7)

barplot(kk, drop = TRUE, showCategory = showNum, color = colorSel)

dev.off()

pdf(file="KEGG_dotplot_DEG.pdf",width = 12,height = 7)

dotplot(kk, showCategory = showNum, orderBy = "GeneRatio",color = colorSel)

dev.off()

pdf(file="KEGG_circos_DEG.pdf",width = 10,height = 7)

kkx=setReadable(kk, 'org.Hs.eg.db', 'ENTREZID')

cnetplot(kkx, foldChange=geneFC,showCategory = 3, circular = TRUE, colorEdge = TRUE)

dev.off()

##########

###########DEG_keygene

nonmetastasis = 27

metastasis = 25

rt=diffSig

diffExp=read.table("symbol_TPM.txt",sep="\t",header=T,check.names=F)

diffExp <- as.matrix(diffExp)

rownames(diffExp)=diffExp[,1]

exp=diffExp[,2:ncol(diffExp)]

dimnames=list(rownames(exp),colnames(exp))

diffExp=matrix(as.numeric(as.matrix(exp)),nrow=nrow(exp),dimnames=dimnames)

gene=read.table("eRNA_list.txt",sep="\t",header=F)

immuneDiffAll=rt[intersect(gene[,1],rownames(rt)),]

immuneDiffGene=intersect(gene[,1],rownames(diffSig))

hmExp=diffExp[immuneDiffGene,]

immuneDiffResult=immuneDiffAll[immuneDiffGene,]

immuneDiffResult=rbind(ID=colnames(immuneDiffResult),immuneDiffResult)

write.table(immuneDiffResult,file="Key_Diff.xls",sep="\t",col.names=F,quote=F)

immuneGeneExp=rbind(ID=colnames(hmExp),hmExp)

write.table(immuneGeneExp,file="Key_GeneExp.txt",sep="\t",quote=F,col.names=F)

#volcano

colnames(immuneDiffAll)[5]= "FDR"

allDiff= immuneDiffAll

allDiff$FDR[allDiff$FDR<=0]=2.22329540628561e-322

#volcano

#plot_mode <- "classic" #经典版

plot_mode <- "advanced" #酷炫版

logFCcut <- 0.3 #log2-foldchange

pvalCut <- 0.05 #P.value

adjPcut <- 0.05 #adj.P.value

#for advanced mode

logFCcut2 <- 1.5

logFCcut3 <- 3

pvalCut2 <- 0.0001

pvalCut3 <- 0.00001

#置x，y軸的最大最小位置

xmin <- (range(allDiff$logFC)[1]- (range(allDiff$logFC)[1]+ 15))

xmax <- (range(allDiff$logFC)[1]+ (15-range(allDiff$logFC)[1]))

ymin <- 0

ymax <- 85

# 基因名的颜色，需大于等于pathway的数量，这里自定义了足够多的颜色

mycol <- c("darkgreen","chocolate4","blueviolet","#223D6C","#D20A13","#088247","#58CDD9","#7A142C","#5D90BA","#431A3D","#91612D","#6E568C","#E0367A","#D8D155","#64495D","#7CC767")

###########plot

if (plot_mode == "classic"){

# 簡單的setting for color

allDiff$color_transparent <- ifelse((allDiff$FDR < pvalCut & allDiff$logFC > logFCcut), "red", ifelse((allDiff$FDR < pvalCut & allDiff$logFC < -logFCcut), "blue","grey"))

# 簡單的setting for size

size <- ifelse((allDiff$FDR < pvalCut & abs(allDiff$logFC) > logFCcut), 4, 2)

} else if (plot_mode == "advanced") {

# 複雜的的setting for color

n1 <- length(allDiff[, 1])

cols <- rep("grey", n1)

names(cols)<- rownames(allDiff)

#不同阈值的点的颜色

cols[allDiff$FDR < pvalCut & allDiff$logFC >logFCcut]<- "#FB9A99"

cols[allDiff$FDR < pvalCut2 & allDiff$logFC > logFCcut2]<- "#ED4F4F"

cols[allDiff$FDR < pvalCut & allDiff$logFC < -logFCcut]<- "#B2DF8A"

cols[allDiff$FDR < pvalCut2 & allDiff$logFC < -logFCcut2]<- "#329E3F"

color_transparent <- adjustcolor(cols, alpha.f = 0.5)

allDiff$color_transparent <- color_transparent

# 複雜的的setting for size

n1 <- length(allDiff[, 1])

size <- rep(1, n1)

#不同阈值的点的大小

size[allDiff$FDR < pvalCut & allDiff$logFC > logFCcut]<- 2

size[allDiff$FDR < pvalCut2 & allDiff$logFC > logFCcut2]<- 4

size[allDiff$FDR < pvalCut3 & allDiff$logFC > logFCcut3]<- 6

size[allDiff$FDR < pvalCut & allDiff$logFC < -logFCcut]<- 2

size[allDiff$FDR < pvalCut2 & allDiff$logFC < -logFCcut2]<- 4

size[allDiff$FDR < pvalCut3 & allDiff$logFC < -logFCcut3]<- 6

} else {

stop("Unsupport mode")

}

# Construct the plot object

p1 <- ggplot(data=allDiff, aes(logFC, -log10(FDR))) +

geom_point(alpha = 0.6, size = size, colour = allDiff$color_transparent) +

labs(allDiff=bquote(~Log[2]~"(fold change)"), y=bquote(~-Log[10]~italic("P-value")), title="") +

ylim(c(ymin,ymax)) +

scale_x_continuous(

breaks = c(-10, -5, -logFCcut, 0, logFCcut, 5, 10), #刻度线的位置

labels = c(-10, -5, -logFCcut, 0, logFCcut, 5, 10),

limits = c(-11, 11) #x轴范围，两侧对称才好看

) +

#或用下面这行：

xlim(c(xmin, xmax)) +

#画阈值分界线

geom_vline(xintercept = c(-logFCcut, logFCcut), color="grey40",

linetype="longdash", lwd = 0.5) + #虚线的形状和粗细

geom_hline(yintercept = -log10(pvalCut), color="grey40",

linetype="longdash", lwd = 0.5) +

theme_bw(base_size = 12#, base_family = "Times" #修改字体

) +

theme(panel.grid=element_blank())

if (plot_mode == "advanced") {

p1 <- p1 +

geom_vline(xintercept = c(-logFCcut2, logFCcut2), color="grey40",

linetype="longdash", lwd = 0.5) +

geom_hline(yintercept = -log10(pvalCut2), color="grey40",

linetype="longdash", lwd = 0.5)

}

pdf(file="vol_keygene.pdf")

p1

dev.off()

#####heatmap

#Type=c(rep("Primary tumor",nonmetastasis),rep("Metastatic tumor",metastasis))

#Type=c(rep("Primary tumor",nonmetastasis),rep("Bone metastatic tumor",metastasis))

#Type=c(rep("Primary tumor",nonmetastasis),rep("Metastatic tumor",metastasis))

#Type=c(rep("Normal_bone",nonmetastasis),rep("Chordoma",metastasis))

#Type=c(rep("Skeletal_muscle",nonmetastasis),rep("Rhabdomyosarcoma",metastasis))

#Type=c(rep("Normal",nonmetastasis),rep("OA",metastasis))

Type=c(rep("Normal blood",nonmetastasis),rep("SCI blood",metastasis))

names(Type)=colnames(hmExp)

Type=as.data.frame(Type)

pdf(file="heatmap_keygene.pdf",height=6,width=10)

pheatmap(hmExp,

annotation=Type,

color =colorRampPalette(c("#377EB8", "white", "#E41A1C"))(50),

cluster_cols =F,

show_colnames = F,

show_rownames = F,

fontsize = 12,

fontsize_row=8,

fontsize_col=10)

dev.off()

#######perl AS.mergeExpTime.pl

################survival

###########KM ANA

setwd("F:\\eRNA数据\\MET500")

library(survival)

library("survminer")

picDir="clinic_OS_KM_picture"

dir.create(picDir)

pFilter=0.05

rt=read.table("expTime.txt",header=T,sep="\t",check.names=F)

rt$futime=rt$futime/12

outTab=data.frame()

setwd(picDir)

for(gene in colnames(rt[,4:ncol(rt)])){

a=rt[,gene]

a=as.vector(ifelse(a>median(a),"high","low"))

diff=survdiff(Surv(futime, fustat) ~a,data = rt)

pValue=1-pchisq(diff$chisq,df=1)

outTab=rbind(outTab,cbind(gene=gene,pvalue=pValue))

fit <- survfit(Surv(futime, fustat) ~ a, data = rt)

summary(fit)

if(pValue<pFilter){

if(pValue<0.001){

pValue=signif(pValue,4)

pValue=format(pValue, scientific = TRUE)

}else{

pValue=round(pValue,3)

}

pdf(file=paste(gene,".survival.pdf",sep=""),onefile = FALSE,

width = 6,

height =5)

p <- ggsurvplot(fit,

data=rt,

conf.int=TRUE,

pval=paste0("P=",pValue),

pval.size=4,

risk.table=TRUE,

legend.labs=levels(a),

legend.title="Risk",

xlab="PFI(months)",

break.time.by = 36,

risk.table.title="",

palette=c("#58CDD9", "#7A142C"),

risk.table.height=.25)

print(p)

dev.off()

}

}

write.table(outTab,file="KMsurvival.xls",sep="\t",row.names=F,quote=F)

###############

setwd("F:\\eRNA数据\\MET500")

library(survival)

pFilter=0.05

rt=read.table("expTime.txt",header=T,sep="\t",check.names=F,row.names=1)

outTab=data.frame()

sigGenes=c("futime","fustat")

for(i in colnames(rt[,3:ncol(rt)])){

cox <- coxph(Surv(futime, fustat) ~ rt[,i], data = rt)

coxSummary = summary(cox)

coxP=coxSummary$coefficients[,"Pr(>|z|)"]

if(coxP<pFilter){

sigGenes=c(sigGenes,i)

outTab=rbind(outTab,

cbind(id=i,

HR=coxSummary$conf.int[,"exp(coef)"],

HR.95L=coxSummary$conf.int[,"lower .95"],

HR.95H=coxSummary$conf.int[,"upper .95"],

pvalue=coxSummary$coefficients[,"Pr(>|z|)"])

)

}

}

write.table(outTab,file="uniCox.txt",sep="\t",row.names=F,quote=F)

uniSigExp=rt[,sigGenes]

uniSigExp=cbind(id=row.names(uniSigExp),uniSigExp)

write.table(uniSigExp,file="uniSigExp.txt",sep="\t",row.names=F,quote=F)

rt <- read.table("uniCox.txt",header=T,sep="\t",row.names=1,check.names=F)

gene <- rownames(rt)

hr <- sprintf("%.5f",rt$"HR")

hrLow <- sprintf("%.5f",rt$"HR.95L")

hrHigh <- sprintf("%.5f",rt$"HR.95H")

Hazard.ratio <- paste0(hr,"(",hrLow,"-",hrHigh,")")

pVal <- ifelse(rt$pvalue<0.00001, "<0.00001", sprintf("%.5f", rt$pvalue))

pdf(file="forest.pdf", width = 10,height =6)

n <- nrow(rt)

nRow <- n+1

ylim <- c(1,nRow)

layout(matrix(c(1,2),nc=2),width=c(3,2.5))

xlim = c(0,3)

par(mar=c(4,2.5,2,1))

plot(1,xlim=xlim,ylim=ylim,type="n",axes=F,xlab="",ylab="")

text.cex=0.8

text(0,n:1,gene,adj=0,cex=text.cex)

text(1.5-0.5*0.2,n:1,pVal,adj=1,cex=text.cex);text(1.5-0.5*0.2,n+1,'pvalue',cex=text.cex,font=2,adj=1)

text(3,n:1,Hazard.ratio,adj=1,cex=text.cex);text(3,n+1,'Hazard ratio',cex=text.cex,font=2,adj=1,)

par(mar=c(4,1,2,1),mgp=c(2,0.5,0))

xlim = c(0,max(as.numeric(hrLow),as.numeric(hrHigh)))

plot(1,xlim=xlim,ylim=ylim,type="n",axes=F,ylab="",xaxs="i",xlab="Hazard ratio")

arrows(as.numeric(hrLow),n:1,as.numeric(hrHigh),n:1,angle=90,code=3,length=0.05,col="darkblue",lwd=2.5)

abline(v=1,col="black",lty=2,lwd=2)

boxcolor = ifelse(as.numeric(hr) > 1,'#E34A33', '#6BAED6')

points(as.numeric(hr), n:1, pch = 15, col = boxcolor, cex=1.3)

axis(1)

dev.off()

########处理uniSigExp.txt

#######lasso

rt=read.table("uniSigExp.txt",header=T,sep="\t",row.names=1,check.names=F)

rt$futime[rt$futime<=0]=1

x=as.matrix(rt[,c(3:ncol(rt))])

y=data.matrix(Surv(rt$futime,rt$fustat))

fit <- glmnet(x, y, family = "cox", maxit = 1000)

pdf("lambda.pdf")

plot(fit, xvar = "lambda", label = TRUE)

dev.off()

cvfit <- cv.glmnet(x, y, family="cox", maxit = 1000)

pdf("cvfit.pdf")

plot(cvfit)

abline(v=log(c(cvfit$lambda.min,cvfit$lambda.1se)),lty="dashed")

dev.off()

coef <- coef(fit, s = cvfit$lambda.min)

index <- which(as.vector(coef != 0))

actCoef <- coef[index]

lassoGene=row.names(coef)[index]

lassoGene=c("futime","fustat",lassoGene)

lassoSigExp=rt[,lassoGene]

lassoSigExp=cbind(id=row.names(lassoSigExp),lassoSigExp)

write.table(lassoSigExp,file="lassoSigExp.txt",sep="\t",row.names=F,quote=F)

########muticox##uniSigExp.txt

rt=read.table("uniSigExp.txt",header=T,sep="\t",check.names=F,row.names=1)

#rt=read.table("lassoSigExp.txt",header=T,sep="\t",check.names=F,row.names=1)

rt$futime=rt$futime

multiCox=coxph(Surv(futime, fustat) ~ ., data = rt)

#multiCox=step(multiCox,direction = "both")

multiCoxSum=summary(multiCox)

outTab=data.frame()

outTab=cbind(

coef=multiCoxSum$coefficients[,"coef"],

HR=multiCoxSum$conf.int[,"exp(coef)"],

HR.95L=multiCoxSum$conf.int[,"lower .95"],

HR.95H=multiCoxSum$conf.int[,"upper .95"],

pvalue=multiCoxSum$coefficients[,"Pr(>|z|)"])

outTab=cbind(id=row.names(outTab),outTab)

outTab=gsub("`","",outTab)

write.table(outTab,file="multiCox.xls",sep="\t",row.names=F,quote=F)

riskScore=predict(multiCox,type="risk",newdata=rt)

coxGene=rownames(multiCoxSum$coefficients)

coxGene=gsub("`","",coxGene)

outCol=c("futime","fustat",coxGene)

risk=as.vector(ifelse(riskScore>median(riskScore),"high","low"))

write.table(cbind(id=rownames(cbind(rt[,outCol],riskScore,risk)),cbind(rt[,outCol],riskScore,risk)),

file="risk.txt",

sep="\t",

quote=F,

row.names=F)

######RISK

Sys.setenv(LANGUAGE = "en") #显示英文报错信息

options(stringsAsFactors = FALSE) #禁止chr转成factor

data <- read.table("risk.txt",sep="\t",header=T,check.names = 1)

data[1:2, 1:8]

# risk score，用于画顶部散点图

rs <- data$riskScore

names(rs) <- rownames(data)

rs_data <- data.frame(x=1:length(rs),rs=as.numeric(sort(rs)))

# 用中值分组

rs_data$Risk <- ifelse(rs_data$rs>=median(rs_data$rs), "High-risk", "Low-risk")

head(rs_data)

# follow-up，用于画中间B图

surv_data <- data.frame(x=1:length(rs),

t=data[names(sort(rs)),'futime'],

s=data[names(sort(rs)),'fustat'])

surv_data$Status <- as.factor(ifelse(surv_data$s==0,'Alive','Death'))

head(surv_data)

######plot.A

pdf("risksline.pdf", 10, 6)

ggplot(rs_data, aes(x=x,y=rs))+

geom_point(aes(col=Risk),size=0.5)+

scale_color_manual(labels=c("High-risk","Low-risk"),

#guide_legend(guide = NULL), #如果不想画图例就删掉#

name="Risk score", values =c("#DC0000FF", "#00A087FF")) +

# 画竖向虚线

geom_segment(aes(x = sum(rs_data$Risk=="Low-risk"),

y = 0,

xend = sum(rs_data$Risk=="Low-risk"),

yend = max(rs_data$rs)), linetype="dashed", size = 0.6)+

# 画横线

#geom_segment(aes(x=0,y=median(rs_data$rs),

# xend=nrow(rs_data),

# yend=median(rs_data$rs)),linetype="dashed", size = 0.3)+

# 写文字Cutoff:

#geom_text(aes(x=sum(rs_data$Risk=="Low-risk")/2,

# y=median(rs_data$rs)+8,

# label=paste0("Cutoff: ",round(median(rs_data$rs),3))),

# col ="black",size = 4,alpha=0.8)+

theme(axis.title.x=element_blank()) +

scale_x_continuous(limits = c(0,NA),expand = c(0,0)) +

labs(y="Risk score",x="",fill="Risk") +

#scale_colour_discrete(name="Risk scores") +

theme_classic() +

theme(axis.ticks.x=element_blank(),

axis.line = element_blank(), #如果想像example2那样画坐标轴，就删掉这行

axis.text.x=element_blank())

dev.off()

#######plot.B

pdf("riskscater.pdf", 10, 6)

ggplot(surv_data,aes(x=x,y=t))+

geom_point(aes(col=Status),size=0.5)+

geom_vline(aes(xintercept=sum(rs_data$Risk=="Low-risk")),size=0.6,linetype="dashed")+

scale_x_continuous(limits = c(0,NA),expand = c(0,0))+

scale_color_manual(labels=c("Alive","Dead"),

values =c("#00A087FF","#DC0000FF"))+

labs(y="OS(months)",x="")+

theme_classic()+

theme(axis.ticks.x=element_blank(),

axis.line = element_blank(), #如果想像example2那样不画坐标轴，就删掉前面的#

axis.text.x=element_blank())

dev.off()

######plot.C

rt=read.table("risk.txt",header=T,sep="\t")

rt$futime=rt$futime/30

diff=survdiff(Surv(futime, fustat) ~risk,data = rt)

pValue=1-pchisq(diff$chisq,df=1)

pValue=signif(pValue,4)

pValue=format(pValue, scientific = TRUE)

fit <- survfit(Surv(futime, fustat) ~ risk, data = rt)

pdf("riskKM.pdf", 10, 8)

ggsurvplot(fit,

data=rt,

conf.int=TRUE,

pval=paste0("p=",pValue),

pval.size=4,

risk.table=TRUE,

legend.labs=c("High risk", "Low risk"),

legend.title="Risk",

xlab="Time(months)",

break.time.by = 12,

risk.table.title="",

palette=c("#00A087FF", "#DC0000FF"),

risk.table.height=.25)

dev.off()

#######roc

library(survivalROC)

rt=read.table("risk.txt",header=T,sep="\t",check.names=F,row.names=1)

rt$futime=rt$futime/365

pdf(file="ROC.pdf",width=6,height=6)

par(oma=c(0.5,1,0,1),font.lab=1.5,font.axis=1.5)

roc=survivalROC(Stime=rt$futime, status=rt$fustat, marker = rt$riskScore,

predict.time =1, method="KM")

plot(roc$FP, roc$TP, type="l", xlim=c(0,1), ylim=c(0,1),col='red',

xlab="False positive rate", ylab="True positive rate",

main=paste("ROC curve (", "AUC = ",sprintf("%.3f",roc$AUC),")"),

lwd = 2, cex.main=1.3, cex.lab=1.2, cex.axis=1.2, font=1.2)

abline(0,1)

dev.off()

##############forest_final

library(survival)

pFilter=0.05

rt=read.table("lassoSigExp.txt",header=T,sep="\t",check.names=F,row.names=1)

outTab=data.frame()

sigGenes=c("futime","fustat")

for(i in colnames(rt[,3:ncol(rt)])){

cox <- coxph(Surv(futime, fustat) ~ rt[,i], data = rt)

coxSummary = summary(cox)

coxP=coxSummary$coefficients[,"Pr(>|z|)"]

if(coxP<pFilter){

sigGenes=c(sigGenes,i)

outTab=rbind(outTab,

cbind(id=i,

HR=coxSummary$conf.int[,"exp(coef)"],

HR.95L=coxSummary$conf.int[,"lower .95"],

HR.95H=coxSummary$conf.int[,"upper .95"],

pvalue=coxSummary$coefficients[,"Pr(>|z|)"])

)

}

}

write.table(outTab,file="uniCox_lasso.txt",sep="\t",row.names=F,quote=F)

uniSigExp=rt[,sigGenes]

uniSigExp=cbind(id=row.names(uniSigExp),uniSigExp)

write.table(uniSigExp,file="uniSigExp_lasso.txt",sep="\t",row.names=F,quote=F)

rt <- read.table("uniCox_lasso.txt",header=T,sep="\t",row.names=1,check.names=F)

gene <- rownames(rt)

hr <- sprintf("%.5f",rt$"HR")

hrLow <- sprintf("%.5f",rt$"HR.95L")

hrHigh <- sprintf("%.5f",rt$"HR.95H")

Hazard.ratio <- paste0(hr,"(",hrLow,"-",hrHigh,")")

pVal <- ifelse(rt$pvalue<0.00001, "<0.00001", sprintf("%.5f", rt$pvalue))

pdf(file="forest_final.pdf", width = 10,height =6)

n <- nrow(rt)

nRow <- n+1

ylim <- c(1,nRow)

layout(matrix(c(1,2),nc=2),width=c(3,2.5))

xlim = c(0,3)

par(mar=c(4,2.5,2,1))

plot(1,xlim=xlim,ylim=ylim,type="n",axes=F,xlab="",ylab="")

text.cex=0.8

text(0,n:1,gene,adj=0,cex=text.cex)

text(1.5-0.5*0.2,n:1,pVal,adj=1,cex=text.cex);text(1.5-0.5*0.2,n+1,'pvalue',cex=text.cex,font=2,adj=1)

text(3,n:1,Hazard.ratio,adj=1,cex=text.cex);text(3,n+1,'Hazard ratio',cex=text.cex,font=2,adj=1,)

par(mar=c(4,1,2,1),mgp=c(2,0.5,0))

xlim = c(0,max(as.numeric(hrLow),as.numeric(hrHigh)))

plot(1,xlim=xlim,ylim=ylim,type="n",axes=F,ylab="",xaxs="i",xlab="Hazard ratio")

arrows(as.numeric(hrLow),n:1,as.numeric(hrHigh),n:1,angle=90,code=3,length=0.05,col="darkblue",lwd=2.5)

abline(v=1,col="black",lty=2,lwd=2)

boxcolor = ifelse(as.numeric(hr) > 1, '#E34A33', '#6BAED6')

points(as.numeric(hr), n:1, pch = 15, col = boxcolor, cex=1.3)

axis(1)

dev.off()

########PCA

library(limma)

library(scatterplot3d)

risk=read.table("risk.txt",sep="\t",header=T,row.names=1)

data=risk[,3:(ncol(risk)-2)]

group=as.vector(risk[,"risk"])

data.class <- rownames(data)

data.pca <- prcomp(data, scale. = TRUE)

color=ifelse(group=="low",3,2)

pcaPredict=predict(data.pca)

pdf(file="riskGene.PCA.pdf",width=5.5,height=5)

s3d=scatterplot3d(pcaPredict[,1:3], pch = 16, color=color)

legend("top", legend = c("Low risk","High risk"),pch = 16, inset = -0.2, xpd = TRUE, horiz = TRUE,col=c(3,2))

dev.off()

##########indpendent factor

###########处理clinical.txt

#############uniIndep

library(survival)

library(forestplot)

clrs <- fpColors(box="#E7B800",line="darkblue", summary="royalblue")

rt=read.table("indepInput.txt",header=T,sep="\t",check.names=F,row.names=1)

rt[,"riskScore"] <- (rt$riskScore-min(rt$riskScore))/(max(rt$riskScore)-min(rt$riskScore))

outTab=data.frame()

for(i in colnames(rt[,3:ncol(rt)])){

cox <- coxph(Surv(futime, fustat) ~ rt[,i], data = rt)

coxSummary = summary(cox)

coxP=coxSummary$coefficients[,"Pr(>|z|)"]

outTab=rbind(outTab,

cbind(id=i,

HR=coxSummary$conf.int[,"exp(coef)"],

HR.95L=coxSummary$conf.int[,"lower .95"],

HR.95H=coxSummary$conf.int[,"upper .95"],

pvalue=coxSummary$coefficients[,"Pr(>|z|)"])

)

}

write.table(outTab,file="uniCox.xls",sep="\t",row.names=F,quote=F)

rt=read.table("uniCox.xls",header=T,sep="\t",row.names=1,check.names=F)

data=as.matrix(rt)

HR=data[,1:3]

hr=sprintf("%.3f",HR[,"HR"])

hrLow=sprintf("%.3f",HR[,"HR.95L"])

hrHigh=sprintf("%.3f",HR[,"HR.95H"])

pVal=data[,"pvalue"]

pVal=ifelse(pVal<0.001, "<0.001", sprintf("%.3f", pVal))

tabletext <-

list(c(NA, rownames(HR)),

append("pvalue", pVal),

append("Hazard ratio",paste0(hr,"(",hrLow,"-",hrHigh,")")) )

pdf(file="uniIndep_forest.pdf",onefile = FALSE,

width = 8,

height = 4,

)

forestplot(tabletext,

rbind(rep(NA, 3), HR),

col=clrs,

graphwidth=unit(50, "mm"),

xlog=T,

lwd.ci=2,

boxsize=0.3,

xlab="Hazard ratio"

)

dev.off()

###########multiIndep

clrs <- fpColors(box="#2E9FDF",line="darkblue", summary="royalblue")

rt=read.table("indepInput.txt",header=T,sep="\t",check.names=F,row.names=1)

multiCox=coxph(Surv(futime, fustat) ~ ., data = rt)

multiCoxSum=summary(multiCox)

outTab=data.frame()

outTab=cbind(

HR=multiCoxSum$conf.int[,"exp(coef)"],

HR.95L=multiCoxSum$conf.int[,"lower .95"],

HR.95H=multiCoxSum$conf.int[,"upper .95"],

pvalue=multiCoxSum$coefficients[,"Pr(>|z|)"])

outTab=cbind(id=row.names(outTab),outTab)

write.table(outTab,file="multiCox.xls",sep="\t",row.names=F,quote=F)

rt=read.table("multiCox.xls",header=T,sep="\t",row.names=1,check.names=F)

data=as.matrix(rt)

HR=data[,1:3]

hr=sprintf("%.3f",HR[,"HR"])

hrLow=sprintf("%.3f",HR[,"HR.95L"])

hrHigh=sprintf("%.3f",HR[,"HR.95H"])

pVal=data[,"pvalue"]

pVal=ifelse(pVal<0.001, "<0.001", sprintf("%.3f", pVal))

tabletext <-

list(c(NA, rownames(HR)),

append("pvalue", pVal),

append("Hazard ratio",paste0(hr,"(",hrLow,"-",hrHigh,")")) )

pdf(file="multiIndep_forest.pdf",onefile = FALSE,

width = 8,

height = 4,

)

forestplot(tabletext,

rbind(rep(NA, 3), HR),

col=clrs,

graphwidth=unit(50, "mm"),

xlog=T,

lwd.ci=2,

boxsize=0.3,

xlab="Hazard ratio"

)

dev.off()

##########clinical_cor

library(limma)

library(ggpubr)

setwd("F:\\eRNA数据\\脊髓损伤外周血\\clinic_cor")

scoreFile="score.txt" #score?ļ?

cliFile="clinical_class.txt" #?ٴ??????ļ?

#??ȡscore?ļ????????????ļ?????

rt=read.table(scoreFile,sep="\t",header=T,check.names=F,row.names=1)

data=as.matrix(rt)

#??ȡ?ٴ??????ļ?

cli=read.table(cliFile,sep="\t",header=T,check.names=F,row.names=1)

#?ϲ?????

samSample=intersect(row.names(data),row.names(cli))

data=data[samSample,]

cli=cli[samSample,]

rt=cbind(data,cli)

#?ٴ??????Է?????????ͼ?ν???

for(clinical in colnames(rt[,(ncol(data)+1):ncol(rt)])){

for(score in colnames(rt[,1:ncol(data)])){

data=rt[c(score,clinical)]

colnames(data)=c("score","clinical")

data=data[(data[,"clinical"]!="unknow"),]

#???ñȽ???

group=levels(factor(data$clinical))

data$clinical=factor(data$clinical, levels=group)

comp=combn(group,2)

my_comparisons=list()

for(i in 1:ncol(comp)){my_comparisons[[i]]<-comp[,i]}

#????boxplot

boxplot=ggboxplot(data, x="clinical", y="score", color="clinical",

xlab=clinical,

ylab=score,

legend.title=clinical,

add = "jitter")+

stat_compare_means(comparisons = my_comparisons)+

theme(axis.text.x = element_text(angle = 45,hjust = 1))

#????ͼƬ

pdf(file=paste0(score,".",clinical,".pdf"),width=6,height=6)

print(boxplot)

dev.off()

}

}

################

inputFile="ProGeneExp1.txt"

yMin=0

yMax=1

ySeg=yMax*0.94

rt=read.table(inputFile,sep="\t",header=T,check.names=F)

clinical="Pain"

picDir="clinic_Pain_bee_picture"

dir.create(picDir)

xlabel=vector()

tab1=table(rt[,clinical])

labelNum=length(tab1)

dotCol=c(2,3)

if(labelNum==3){

dotCol=c(2,3,4)

}

if(labelNum==4){

dotCol=c(2,3,4,5)

}

if(labelNum>4){

dotCol=rainbow(labelNum)

}

for(i in 1:labelNum){

xlabel=c(xlabel,names(tab1[i]) )

}

outTab=data.frame()

for(i in colnames(rt[,3:ncol(rt)])){

rt1=rbind(expression=rt[,i],clinical=rt[,clinical])

rt1=as.matrix(t(rt1))

if(labelNum==2){

wilcoxTest<-wilcox.test(expression ~ clinical, data=rt1)

}else{

wilcoxTest<-kruskal.test(expression ~ clinical, data = rt1)}

pValue=wilcoxTest$p.value

outTab=rbind(outTab,cbind(gene=i,pVal=pValue))

pval=0

if(pValue<0.001){

pval=signif(pValue,4)

pval=format(pval, scientific = TRUE)

}else{

pval=round(pValue,3)

}

b = boxplot(expression ~ clinical, data = rt1,outline = FALSE, plot=F)

yMin=min(b$stats)

yMax = max(b$stats/5+b$stats)

ySeg = max(b$stats/10+b$stats)

ySeg2 = max(b$stats/12+b$stats)

n = ncol(b$stats)

tiffFile=paste0(i,".",clinical,".tiff")

outTiff=paste(picDir,tiffFile,sep="\\")

tiff(file=outTiff,width = 15,height = 9,

units ="cm",compression="lzw",bg="white",res=600)

par(mar = c(4,7,3,3))

boxplot(expression ~ clinical, data = rt1,names=xlabel,

ylab = paste(i," expression",sep=""),

cex.main=1.5, cex.lab=1.0, cex.axis=0.6,ylim=c(yMin,yMax),outline = FALSE)

#E69F00

#56B4E9

#009E73

#D55E00

#CC79A7

beeswarm(expression ~ clinical, data = rt1, col = c("#E69F00","#56B4E9","#009E73","#D55E00","#CC79A7"),lwd=0.1,

pch = 16, add = TRUE, corral="wrap")

#segments(1,ySeg, 2,ySeg);segments(1,ySeg, 1,ySeg*0.96);segments(2,ySeg, 2,ySeg*0.96)

text(1.5,ySeg*1.05,labels=paste("p=",pval,sep=""),cex=1.2)

dev.off()

}

write.table(outTab,file=paste0(clinical,".xls"),sep="\t",row.names=F,quote=F)

##########clinical_cor

library(ggpubr)

rt=read.table("risk.txt",sep="\t",header=T,row.names=1,check.names=F) #??ȡ?ļ?

rt=rt[,3:(ncol(rt)-2)]

Type=read.table("clinical_cor.txt",sep="\t",check.names=F,header=T)

row.names(Type)=Type[,1]

sameSample=intersect(row.names(Type),row.names(rt))

rt=rt[sameSample,]

Type=Type[sameSample,]

data=data.frame()

for(i in colnames(rt)){

data=rbind(data,cbind(expression=rt[,i],gene=i,Type=as.vector(Type[,2])))

}

write.table(data,file="data.txt",sep="\t",row.names=F,quote=F)

data=read.table("data.txt",sep="\t",header=T,check.names=F)

p=ggboxplot(data, x="gene", y="expression", color = "Type",

ylab="lncRNA expression",

xlab="",

palette = c("#9ECAE1","#BCBDDC","#FEE0D2","#800026","#FF3434"))

p=p+rotate_x_text(45)

pdf(file=paste(colnames(Type)[2],"boxplot.pdf"),width=15,height=8)

p+stat_compare_means(aes(group=Type),symnum.args=list(cutpoints = c(0, 0.001, 0.01, 0.05, 1), symbols = c("***", "**", "*", "")),label = "p.signif")

dev.off()

#########

setwd("F:\\eRNA数据\\脊髓损伤外周血")

rt <- read.table("CIBERSORT.filter.txt",header=T,sep="\t",check.names=F,row.names=1)

rt= t(rt)

data <- read.table("Key_GeneExp.txt",header=T,sep="\t",check.names=F,row.names=1)

genes = intersect(rownames(rt),colnames(data))

data = rt[genes,]

outpdf="barplot.pdf"

data=t(data)

# 自定义配色方案

library(scales)

mycols <- c(brewer_pal(palette = "Reds")(4),

brewer_pal(palette = "Blues")(4),

brewer_pal(palette = "Purples")(5),

brewer_pal(palette = "YlOrRd")(5),

brewer_pal(palette = "OrRd")(4))

pdf(outpdf,height=10,width=20)

par(las=1,mar=c(8,4,4,15))

a1 = barplot(data,col=mycols,yaxt="n",ylab="Relative Percent",xaxt="n",border = NA)

a2=axis(2,tick=F,labels=F)

axis(2,a2,paste0(a2*100,"%"))

#axis(1,a1,labels=F)

par(srt=60,xpd=T);text(a1,-0.02,colnames(data),adj=1,cex=0.4);par(srt=0)

ytick2 = cumsum(data[,ncol(data)])

ytick1 = c(0,ytick2[-length(ytick2)])

#text(par('usr')[2],(ytick1+ytick2)/2,rownames(data),cex=0.6,adj=0)

legend(par('usr')[2]*0.98,par('usr')[4],legend=rownames(data),col=mycols,pch=15,bty="n",cex=1.3)

dev.off()

####6.corrplot heatmap##################################################################

rt <- read.table("CIBERSORT.filter.txt",header=T,sep="\t",check.names=F,row.names=1)

library(corrplot)

pdf("corHeatmap_CIBERSORT.pdf",height=13,width=13)

corrplot(corr=cor(t(rt)),

method = "color",

order = "hclust",

tl.col="black",

addCoef.col = "black",

number.cex = 0.8,

col=colorRampPalette(c("#377EB8", "white", "#E41A1C"))(50)

)

dev.off()

rt=rbind(id=colnames(rt),rt)

write.table(rt,file="CIBERSORT.filter_trans.txt",sep="\t",row.names=T,quote=F,col.names = F)

##########clinical_cor

library(ggpubr)

rt=read.table("CIBERSORT.filter_trans.txt",sep="\t",header=T,row.names=1,check.names=F) #??ȡ?ļ?

rt= t(rt)

Type=read.table("clinical_cor_CIBERSORT.txt",sep="\t",check.names=F,header=T)

row.names(Type)=Type[,1]

sameSample=intersect(row.names(Type),row.names(rt))

rt=rt[sameSample,]

Type=Type[sameSample,]

data=data.frame()

for(i in colnames(rt)){

data=rbind(data,cbind(expression=rt[,i],gene=i,Type=as.vector(Type[,2])))

}

write.table(data,file="data_CIBESORT.txt",sep="\t",row.names=F,quote=F)

data=read.table("data_CIBESORT.txt",sep="\t",header=T,check.names=F)

p=ggboxplot(data, x="gene", y="expression", color = "Type",

ylab="Immune cell fraction",

xlab="",

palette = c("#9ECAE1","#800026","#BCBDDC","#FEE0D2"))

p=p+rotate_x_text(45)

pdf(file=paste(colnames(Type)[2],"boxplot_CIBESORT.pdf"),,width=10,height=8)

p+stat_compare_means(aes(group=Type),symnum.args=list(cutpoints = c(0, 0.001, 0.01, 0.05, 1), symbols = c("***", "**", "*", "")),label = "p.signif")

dev.off()

##########

#########ssGSEA

inputFile="symbol_TPM.txt" #输入文件

gmtFile="F:\\桌面数据复制2019-10-18\\桌面\\ssGSEA\\矩阵处理\\immune.gmt" #GMT文件

#引用包

library(GSVA)

library(limma)

library(GSEABase)

#读取输入文件，并对输入文件处理

rt=read.table(inputFile,sep="\t",header=T,check.names=F)

rt=as.matrix(rt)

rownames(rt)=rt[,1]

exp=rt[,2:ncol(rt)]

dimnames=list(rownames(exp),colnames(exp))

mat=matrix(as.numeric(as.matrix(exp)),nrow=nrow(exp),dimnames=dimnames)

mat=avereps(mat)

mat=mat[rowMeans(mat)>0,]

geneSet=getGmt(gmtFile,

geneIdType=SymbolIdentifier())

#ssgsea分析

ssgseaScore=gsva(mat, geneSet, method='ssgsea', kcdf='Gaussian', abs.ranking=TRUE)

#定义ssGSEA score矫正函数

normalize=function(x){

return((x-min(x))/(max(x)-min(x)))}

#对ssGSEA score进行矫正

ssgseaOut=normalize(ssgseaScore)

ssgseaOut=rbind(id=colnames(ssgseaOut),ssgseaOut)

write.table(ssgseaOut,file="ssgseaOut.txt",sep="\t",quote=F,col.names=F)

rt=read.table("ssgseaOut.txt",sep="\t",header=T,check.names=F,row.names = 1)

library(pheatmap)

Type=read.table("clinical_initial.txt",sep="\t",header=T,check.names=F,row.names = 1)

#Type=c(rep("normal whole blood",nonmetastasis),rep("Septic shock blood",metastasis))

#Type=c(rep("non-metastasis",nonmetastasis),rep("metastasis",metastasis))

#Type=c(rep("primarytumor",nonmetastasis),rep("new_tumor_event",metastasis))

Type=as.data.frame(Type)

pdf(file="heatmap_ssgsea.pdf", width = 10,height =6)

pheatmap(rt,

annotation=Type,

color = colorRampPalette(c("#377EB8", "white", "#E41A1C"))(50),

cluster_cols =F,

fontsize = 8,

show_colnames = F,

fontsize_row=8,

fontsize_col=4)

dev.off()

#########

######TF

#########差异

rt=diffSig

diffExp=read.table("symbol_TPM.txt",sep="\t",header=T,check.names=F)

diffExp <- as.matrix(diffExp)

rownames(diffExp)=diffExp[,1]

exp=diffExp[,2:ncol(diffExp)]

dimnames=list(rownames(exp),colnames(exp))

diffExp=matrix(as.numeric(as.matrix(exp)),nrow=nrow(exp),dimnames=dimnames)

gene=read.table("TF.txt",sep="\t",header=F)

immuneDiffAll=rt[intersect(gene[,1],rownames(rt)),]

immuneDiffGene=intersect(gene[,1],rownames(diffSig))

hmExp=diffExp[immuneDiffGene,]

immuneDiffResult=immuneDiffAll[immuneDiffGene,]

immuneDiffResult=rbind(ID=colnames(immuneDiffResult),immuneDiffResult)

write.table(immuneDiffResult,file="Key_TF.xls",sep="\t",col.names=F,quote=F)

immuneGeneExp=rbind(ID=colnames(hmExp),hmExp)

write.table(immuneGeneExp,file="Key_TF.txt",sep="\t",quote=F,col.names=F)

#volcano

colnames(immuneDiffAll)[5]= "FDR"

allDiff= immuneDiffAll

allDiff$FDR[allDiff$FDR<=0]=2.22329540628561e-322

#volcano

#plot_mode <- "classic" #经典版

plot_mode <- "advanced" #酷炫版

logFCcut <- 0.3 #log2-foldchange

pvalCut <- 0.05 #P.value

adjPcut <- 0.05 #adj.P.value

#for advanced mode

logFCcut2 <- 1.5

logFCcut3 <- 3

pvalCut2 <- 0.0001

pvalCut3 <- 0.00001

#置x，y軸的最大最小位置

xmin <- (range(allDiff$logFC)[1]- (range(allDiff$logFC)[1]+ 15))

xmax <- (range(allDiff$logFC)[1]+ (15-range(allDiff$logFC)[1]))

ymin <- 0

ymax <- 85

# 基因名的颜色，需大于等于pathway的数量，这里自定义了足够多的颜色

mycol <- c("darkgreen","chocolate4","blueviolet","#223D6C","#D20A13","#088247","#58CDD9","#7A142C","#5D90BA","#431A3D","#91612D","#6E568C","#E0367A","#D8D155","#64495D","#7CC767")

###########plot

if (plot_mode == "classic"){

# 簡單的setting for color

allDiff$color_transparent <- ifelse((allDiff$FDR < pvalCut & allDiff$logFC > logFCcut), "red", ifelse((allDiff$FDR < pvalCut & allDiff$logFC < -logFCcut), "blue","grey"))

# 簡單的setting for size

size <- ifelse((allDiff$FDR < pvalCut & abs(allDiff$logFC) > logFCcut), 4, 2)

} else if (plot_mode == "advanced") {

# 複雜的的setting for color

n1 <- length(allDiff[, 1])

cols <- rep("grey", n1)

names(cols)<- rownames(allDiff)

#不同阈值的点的颜色

cols[allDiff$FDR < pvalCut & allDiff$logFC >logFCcut]<- "#FB9A99"

cols[allDiff$FDR < pvalCut2 & allDiff$logFC > logFCcut2]<- "#ED4F4F"

cols[allDiff$FDR < pvalCut & allDiff$logFC < -logFCcut]<- "#B2DF8A"

cols[allDiff$FDR < pvalCut2 & allDiff$logFC < -logFCcut2]<- "#329E3F"

color_transparent <- adjustcolor(cols, alpha.f = 0.5)

allDiff$color_transparent <- color_transparent

# 複雜的的setting for size

n1 <- length(allDiff[, 1])

size <- rep(1, n1)

#不同阈值的点的大小

size[allDiff$FDR < pvalCut & allDiff$logFC > logFCcut]<- 2

size[allDiff$FDR < pvalCut2 & allDiff$logFC > logFCcut2]<- 4

size[allDiff$FDR < pvalCut3 & allDiff$logFC > logFCcut3]<- 6

size[allDiff$FDR < pvalCut & allDiff$logFC < -logFCcut]<- 2

size[allDiff$FDR < pvalCut2 & allDiff$logFC < -logFCcut2]<- 4

size[allDiff$FDR < pvalCut3 & allDiff$logFC < -logFCcut3]<- 6

} else {

stop("Unsupport mode")

}

# Construct the plot object

p1 <- ggplot(data=allDiff, aes(logFC, -log10(FDR))) +

geom_point(alpha = 0.6, size = size, colour = allDiff$color_transparent) +

labs(allDiff=bquote(~Log[2]~"(fold change)"), y=bquote(~-Log[10]~italic("P-value")), title="") +

ylim(c(ymin,ymax)) +

scale_x_continuous(

breaks = c(-10, -5, -logFCcut, 0, logFCcut, 5, 10), #刻度线的位置

labels = c(-10, -5, -logFCcut, 0, logFCcut, 5, 10),

limits = c(-11, 11) #x轴范围，两侧对称才好看

) +

#或用下面这行：

xlim(c(xmin, xmax)) +

#画阈值分界线

geom_vline(xintercept = c(-logFCcut, logFCcut), color="grey40",

linetype="longdash", lwd = 0.5) + #虚线的形状和粗细

geom_hline(yintercept = -log10(pvalCut), color="grey40",

linetype="longdash", lwd = 0.5) +

theme_bw(base_size = 12#, base_family = "Times" #修改字体

) +

theme(panel.grid=element_blank())

if (plot_mode == "advanced") {

p1 <- p1 +

geom_vline(xintercept = c(-logFCcut2, logFCcut2), color="grey40",

linetype="longdash", lwd = 0.5) +

geom_hline(yintercept = -log10(pvalCut2), color="grey40",

linetype="longdash", lwd = 0.5)

}

pdf(file="vol_TF.pdf")

p1

dev.off()

#####heatmap

#Type=c(rep("Primary tumor",nonmetastasis),rep("Metastatic tumor",metastasis))

#Type=c(rep("Primary tumor",nonmetastasis),rep("Bone metastatic tumor",metastasis))

#Type=c(rep("Normal_bone",nonmetastasis),rep("Chordoma",metastasis))

#Type=c(rep("Skeletal_muscle",nonmetastasis),rep("Rhabdomyosarcoma",metastasis))

#Type=c(rep("Normal_bone",nonmetastasis),rep("Ewing_sarcoma",metastasis))

Type=c(rep("Normal blood",nonmetastasis),rep("SCI blood",metastasis))

names(Type)=colnames(hmExp)

Type=as.data.frame(Type)

pdf(file="heatmap_TF.pdf", width = 10,height =6)

pheatmap(hmExp,

annotation=Type,

color =colorRampPalette(c("#3F007D", "white", "#E34A33"))(50),

cluster_cols =F,

show_colnames = F,

show_rownames = T,

fontsize = 12,

fontsize_row=6,

fontsize_col=10)

dev.off()

##########GSVA

load("F:\\新课程\\小丫画图\\GSVA\\hallmark.gs.RData")

library(GSVA)

library(limma)

library(GSEABase)

rt=read.table("symbol_TPM.txt",sep="\t",header=T,check.names=F)

rt <- as.matrix(rt)

rownames(rt)=rt[,1]

exp=rt[,2:ncol(rt)]

dimnames=list(rownames(exp),colnames(exp))

gsym.expr=matrix(as.numeric(as.matrix(exp)),nrow=nrow(exp),dimnames=dimnames)

head(gsym.expr)

gsva_es <- gsva(as.matrix(gsym.expr), gs)

write.csv(gsva_es, "gsva_out.csv", quote = F)

# differential analysis

logFCcutoff=0.0001

adjPvalueCutoff=0.05

group_list <- data.frame(sample = colnames(gsva_es), group = c(rep("Nonmetastasis", nonmetastasis), rep("Metastasis", metastasis)))

head(group_list)

# 设置对比

design <- model.matrix(~ 0 + factor(group_list$group))

colnames(design) <- levels(factor(group_list$group))

rownames(design) <- colnames(gsva_es)

# 构建差异比较矩阵

contrast.matrix <- makeContrasts(Nonmetastasis, levels = design)

# 差异分析，b vs. a

fit <- lmFit(gsva_es, design)

fit2 <- contrasts.fit(fit, contrast.matrix)

fit2 <- eBayes(fit2)

x <- topTable(fit2, coef = 1, n = Inf, adjust.method = "BH", sort.by = "P")

head(x)

#把通路的limma分析结果保存到文件

write.csv(x, "gsva_limma.csv", quote = F)

#输出t值，用做FigureYa39bar的输入数据

pathway <- str_replace(row.names(x), "HALLMARK_", "")

df <- data.frame(ID = pathway, score = -x$t)

write.csv(df, "easy_input2_for39bar.csv", quote = F, row.names = F)

df <- read.csv("easy_input2_for39bar.csv")

head(df)

#按照score的值分组

cutoff <- 1

df$group <- cut(df$score, breaks = c(-Inf, -cutoff, cutoff, Inf),labels = c(1,2,3))

#按照score排序

sortdf <- df[order(df$score),]

sortdf$ID <- factor(sortdf$ID, levels = sortdf$ID)

head(sortdf)

pdf("gsva.pdf", width = 9, height = 12)

ggplot(sortdf, aes(ID, score, fill = group)) + geom_bar(stat = 'identity') +

coord_flip() +

scale_fill_manual(values = c('#377EB8', 'snow3', '#E41A1C'), guide = FALSE) +

#画2条虚线

geom_hline(yintercept = c(-cutoff,cutoff),

color="white",

linetype = 2, #画虚线

size = 0.3) + #线的粗细

#写label

geom_text(data = subset(df, score > 0),

aes(x=ID, y= -0.05, label= paste0(" ", ID), color = group),#bar跟坐标轴间留出间隙

size = 3, #字的大小

hjust = "inward" ) + #字的对齐方式

geom_text(data = subset(df, score < 0),

aes(x=ID, y= 0.05, label=ID, color = group),

size = 3, hjust = "outward") +

scale_colour_manual(values = c("black","snow3","black"), guide = FALSE) +

xlab("") +ylab("t value of GSVA score")+

theme_bw() + #去除背景色

theme(panel.grid =element_blank()) + #去除网格线

theme(panel.border = element_rect(size = 0.6)) + #边框粗细

theme(axis.line.y = element_blank(), axis.ticks.y = element_blank(), axis.text.y = element_blank()) #去除y轴

dev.off()

######vol

#输出t值，用做FigureYa39bar的输入数据

pathway <- str_replace(row.names(x), "HALLMARK_", "")

rownames(x) <- pathway

allDiff=x

allDiff$logFC=-allDiff$logFC

plot_mode <- "advanced" #酷炫版

logFCcut <- 0.01 #log2-foldchange

pvalCut <- 0.05 #P.value

adjPcut <- 0.05 #adj.P.value

#for advanced mode

logFCcut2 <- 0.05

logFCcut3 <- 0.10

pvalCut2 <- 0.01

pvalCut3 <- 0.001

#置x，y軸的最大最小位置

xmin <- (range(allDiff$logFC)[1]- (range(allDiff$logFC)[1]+ 0.5))

xmax <- (range(allDiff$logFC)[1]+ (0.5-range(allDiff$logFC)[1]))

ymin <- 0

ymax <- -log10(allDiff$P.Value)[1] * 1.2

# 基因名的颜色，需大于等于pathway的数量，这里自定义了足够多的颜色

mycol <- c("darkgreen","chocolate4","blueviolet","#223D6C","#D20A13","#088247","#58CDD9","#7A142C","#5D90BA","#431A3D","#91612D","#6E568C","#E0367A","#D8D155","#64495D","#7CC767")

###########plot

if (plot_mode == "classic"){

# 簡單的setting for color

allDiff$color_transparent <- ifelse((allDiff$P.Value < pvalCut & allDiff$logFC > logFCcut), "red", ifelse((allDiff$P.Value < pvalCut & allDiff$logFC < -logFCcut), "blue","grey"))

# 簡單的setting for size

size <- ifelse((allDiff$P.Value < pvalCut & abs(allDiff$logFC) > logFCcut), 4, 2)

} else if (plot_mode == "advanced") {

# 複雜的的setting for color

n1 <- length(allDiff[, 1])

cols <- rep("grey", n1)

names(cols)<- rownames(allDiff)

#不同阈值的点的颜色

cols[allDiff$P.Value < pvalCut & allDiff$logFC >logFCcut]<- "#FB9A99"

cols[allDiff$P.Value < pvalCut2 & allDiff$logFC > logFCcut2]<- "#ED4F4F"

cols[allDiff$P.Value < pvalCut & allDiff$logFC < -logFCcut]<- "#B2DF8A"

cols[allDiff$P.Value < pvalCut2 & allDiff$logFC < -logFCcut2]<- "#329E3F"

color_transparent <- adjustcolor(cols, alpha.f = 0.5)

allDiff$color_transparent <- color_transparent

# 複雜的的setting for size

n1 <- length(allDiff[, 1])

size <- rep(1, n1)

#不同阈值的点的大小

size[allDiff$P.Value < pvalCut & allDiff$logFC > logFCcut]<- 2

size[allDiff$P.Value < pvalCut2 & allDiff$logFC > logFCcut2]<- 4

size[allDiff$P.Value < pvalCut3 & allDiff$logFC > logFCcut3]<- 6

size[allDiff$P.Value < pvalCut & allDiff$logFC < -logFCcut]<- 2

size[allDiff$P.Value < pvalCut2 & allDiff$logFC < -logFCcut2]<- 4

size[allDiff$P.Value < pvalCut3 & allDiff$logFC < -logFCcut3]<- 6

} else {

stop("Unsupport mode")

}

# Construct the plot object

p1 <- ggplot(data=allDiff, aes(logFC, -log10(P.Value))) +

geom_point(alpha = 0.6, size = size, colour = allDiff$color_transparent) +

labs(allDiff=bquote(~Log[2]~"(fold change)"), y=bquote(~-Log[10]~italic("P-value")), title="") +

ylim(c(ymin,ymax)) +

#scale_x_continuous(

# breaks = c(-10, -5, -logFCcut, 0, logFCcut, 5, 10), #刻度线的位置

#labels = c(-10, -5, -logFCcut, 0, logFCcut, 5, 10),

#limits = c(-11, 11) #x轴范围，两侧对称才好看

#) +

#或用下面这行：

xlim(c(xmin, xmax)) +

#画阈值分界线

geom_vline(xintercept = c(-logFCcut, logFCcut), color="grey40",

linetype="longdash", lwd = 0.5) + #虚线的形状和粗细

geom_hline(yintercept = -log10(pvalCut), color="grey40",

linetype="longdash", lwd = 0.5) +

theme_bw(base_size = 12#, base_family = "Times" #修改字体

) +

theme(panel.grid=element_blank())

if (plot_mode == "advanced") {

p1 <- p1 +

geom_vline(xintercept = c(-logFCcut2, logFCcut2), color="grey40",

linetype="longdash", lwd = 0.5) +

geom_hline(yintercept = -log10(pvalCut2), color="grey40",

linetype="longdash", lwd = 0.5)

}

pdf(file="vol_HM.pdf", width = 7,height =7)

p1

dev.off()

##########heatmap

diff <- topTable(fit2, coef = 1, n = Inf, adjust.method = "BH", sort.by = "P", p.value=adjPvalueCutoff)

diffName=row.names(diff)

write.table(diff,file="diff_HM.xls",sep="\t",quote=F,col.names=F)

hmExp=gsva_es[diffName,]

hmExp=rbind(id=colnames(hmExp),hmExp)

write.table(hmExp,file="heatmap_HM.txt",sep="\t",quote=F,col.names=F)

rt=gsva_es

library(pheatmap)

#Type=c(rep("Primary tumor",nonmetastasis),rep("Metastatic tumor",metastasis))

#Type=c(rep("Normal_bone",nonmetastasis),rep("Chordoma",metastasis))

#Type=c(rep("Primary tumor",nonmetastasis),rep("Bone metastatic tumor",metastasis))

#Type=c(rep("non-metastasis",nonmetastasis),rep("metastasis",metastasis))

#Type=c(rep("primarytumor",nonmetastasis),rep("new_tumor_event",metastasis))

#Type=c(rep("Skeletal_muscle",nonmetastasis),rep("Rhabdomyosarcoma",metastasis))

#Type=c(rep("Normal",nonmetastasis),rep("OA",metastasis))

Type=c(rep("Normal blood",nonmetastasis),rep("SCI blood",metastasis))

names(Type)=colnames(rt)

Type=as.data.frame(Type)

pdf(file="heatmap_HM.pdf", width = 10,height =6)

pheatmap(rt,

annotation=Type,

color = colorRampPalette(c("#377EB8", "white", "#E41A1C"))(50),

cluster_cols =F,

fontsize = 8,

show_colnames = F,

fontsize_row=6,

fontsize_col=4)

dev.off()

######colorRampPalette(c("green", "black", "red"))(50)

######bk <- c(seq(-9,-0.1,by=0.01),seq(0,9,by=0.01))

######c(colorRampPalette(colors = c("blue","white"))(length(bk)/2),colorRampPalette(colors = c("white","red"))(length(bk)/2)),

#############target

setwd("F:\\eRNA数据\\脊髓损伤外周血")

library(limma)

library(ggplot2)

library(ggpubr)

library(ggExtra)

corFilter=0.80

pvalueFilter=0.05

expFile="symbol_TPM.txt"

rt=read.table(expFile,sep="\t",header=T,check.names=F)

rt=as.matrix(rt)

rownames(rt)=rt[,1]

exp=rt[,2:ncol(rt)]

dimnames=list(rownames(exp),colnames(exp))

data=matrix(as.numeric(as.matrix(exp)),nrow=nrow(exp),dimnames=dimnames)

data=avereps(data)

data=data[rowMeans(data)>0,]

eRNA = read.table("Key_GeneExp.txt", row.names=1 ,header=T,sep="\t",check.names=F)

uniSigExp = read.table("uniSigExp.txt", row.names=1 ,header=T,sep="\t",check.names=F)

uniSigExp= uniSigExp[,-1]

uniSigExp= uniSigExp[,-1]

gene1 = colnames(uniSigExp)

eRNA= eRNA[gene1,]

#新建eRNAsymbol.txt

target=read.table("eRNAsymbol.txt",sep="\t",header=T,check.names=F)

targetDup=target[!duplicated(target),]

targetDup=targetDup[which(targetDup[,1] %in% row.names(eRNA)),]

targetDup=targetDup[which(targetDup[,3] %in% row.names(data)),]

picDir="cor_picture"

dir.create(picDir)

setwd(picDir)

#?????Լ???

outTab=data.frame()

for(n in 1:nrow(targetDup)){

i=as.character(targetDup[n,1])

j=as.character(targetDup[n,3])

x=as.numeric(data[i,])

y=as.numeric(data[j,])

corT=cor.test(x,y,method="pearson")

cor=corT$estimate

pvalue=corT$p.value

if((cor>corFilter) & (pvalue<pvalueFilter)){

outTab=rbind(outTab,cbind(eRNA=i,Target=j,cor=cor,corPval=pvalue))

#??????????????

df1=as.data.frame(cbind(x,y))

p1=ggplot(df1, aes(x, y)) +

xlab(i)+ylab(j)+

geom_point()+ geom_smooth(method="lm",formula=y~x) + theme_bw()+

stat_cor(method = 'pearson', aes(x =x, y =y))

pdf(file=paste0("cor.",i,"_",j,".pdf"),width=5,height=5)

print(p1)

dev.off()

}

}

#?????????Խ???

write.table(file="corResult_eRNA.xls",outTab,sep="\t",quote=F,row.names=F)

##########

setwd("F:\\eRNA数据\\脊髓损伤外周血")

write.table(file="corResult_eRNA.txt",outTab,sep="\t",quote=F,row.names=F)

write.table(file="target_list.txt",outTab[,2],sep="\t",quote=F,row.names=F)

###########target

setwd("F:\\eRNA数据\\脊髓损伤外周血")

#########差异分

fdrFilter=0.05

rt=diffSig

diffExp=read.table("symbol_TPM.txt",sep="\t",header=T,check.names=F)

diffExp <- as.matrix(diffExp)

rownames(diffExp)=diffExp[,1]

exp=diffExp[,2:ncol(diffExp)]

dimnames=list(rownames(exp),colnames(exp))

diffExp=matrix(as.numeric(as.matrix(exp)),nrow=nrow(exp),dimnames=dimnames)

gene=read.table("target_list.txt",sep="\t",header=T)

immuneDiffAll=rt[intersect(gene[,1],rownames(rt)),]

immuneDiffGene=intersect(gene[,1],rownames(diffSig))

hmExp=diffExp[immuneDiffGene,]

immuneDiffResult=immuneDiffAll[immuneDiffGene,]

immuneDiffResult=rbind(ID=colnames(immuneDiffResult),immuneDiffResult)

write.table(immuneDiffResult,file="target_diff.xls",sep="\t",col.names=F,quote=F)

immuneGeneExp=rbind(ID=colnames(hmExp),hmExp)

write.table(immuneGeneExp,file="target_geneExp.txt",sep="\t",quote=F,col.names=F)

#volcano

colnames(immuneDiffAll)[5]= "FDR"

allDiff= immuneDiffAll

allDiff$FDR[allDiff$FDR<=0]=2.22329540628561e-322

#volcano

#plot_mode <- "classic" #经典版

plot_mode <- "advanced" #酷炫版

logFCcut <- 0.3 #log2-foldchange

pvalCut <- 0.05 #P.value

adjPcut <- 0.05 #adj.P.value

#for advanced mode

logFCcut2 <- 1.5

logFCcut3 <- 3

pvalCut2 <- 0.0001

pvalCut3 <- 0.00001

#置x，y軸的最大最小位置

xmin <- (range(allDiff$logFC)[1]- (range(allDiff$logFC)[1]+ 15))

xmax <- (range(allDiff$logFC)[1]+ (15-range(allDiff$logFC)[1]))

ymin <- 0

ymax <- 85

# 基因名的颜色，需大于等于pathway的数量，这里自定义了足够多的颜色

mycol <- c("darkgreen","chocolate4","blueviolet","#223D6C","#D20A13","#088247","#58CDD9","#7A142C","#5D90BA","#431A3D","#91612D","#6E568C","#E0367A","#D8D155","#64495D","#7CC767")

###########plot

if (plot_mode == "classic"){

# 簡單的setting for color

allDiff$color_transparent <- ifelse((allDiff$FDR < pvalCut & allDiff$logFC > logFCcut), "red", ifelse((allDiff$FDR < pvalCut & allDiff$logFC < -logFCcut), "blue","grey"))

# 簡單的setting for size

size <- ifelse((allDiff$FDR < pvalCut & abs(allDiff$logFC) > logFCcut), 4, 2)

} else if (plot_mode == "advanced") {

# 複雜的的setting for color

n1 <- length(allDiff[, 1])

cols <- rep("grey", n1)

names(cols)<- rownames(allDiff)

#不同阈值的点的颜色

cols[allDiff$FDR < pvalCut & allDiff$logFC >logFCcut]<- "#FB9A99"

cols[allDiff$FDR < pvalCut2 & allDiff$logFC > logFCcut2]<- "#ED4F4F"

cols[allDiff$FDR < pvalCut & allDiff$logFC < -logFCcut]<- "#B2DF8A"

cols[allDiff$FDR < pvalCut2 & allDiff$logFC < -logFCcut2]<- "#329E3F"

color_transparent <- adjustcolor(cols, alpha.f = 0.5)

allDiff$color_transparent <- color_transparent

# 複雜的的setting for size

n1 <- length(allDiff[, 1])

size <- rep(1, n1)

#不同阈值的点的大小

size[allDiff$FDR < pvalCut & allDiff$logFC > logFCcut]<- 2

size[allDiff$FDR < pvalCut2 & allDiff$logFC > logFCcut2]<- 4

size[allDiff$FDR < pvalCut3 & allDiff$logFC > logFCcut3]<- 6

size[allDiff$FDR < pvalCut & allDiff$logFC < -logFCcut]<- 2

size[allDiff$FDR < pvalCut2 & allDiff$logFC < -logFCcut2]<- 4

size[allDiff$FDR < pvalCut3 & allDiff$logFC < -logFCcut3]<- 6

} else {

stop("Unsupport mode")

}

# Construct the plot object

p1 <- ggplot(data=allDiff, aes(logFC, -log10(FDR))) +

geom_point(alpha = 0.6, size = size, colour = allDiff$color_transparent) +

labs(allDiff=bquote(~Log[2]~"(fold change)"), y=bquote(~-Log[10]~italic("P-value")), title="") +

ylim(c(ymin,ymax)) +

scale_x_continuous(

breaks = c(-10, -5, -logFCcut, 0, logFCcut, 5, 10), #刻度线的位置

labels = c(-10, -5, -logFCcut, 0, logFCcut, 5, 10),

limits = c(-11, 11) #x轴范围，两侧对称才好看

) +

#或用下面这行：

xlim(c(xmin, xmax)) +

#画阈值分界线

geom_vline(xintercept = c(-logFCcut, logFCcut), color="grey40",

linetype="longdash", lwd = 0.5) + #虚线的形状和粗细

geom_hline(yintercept = -log10(pvalCut), color="grey40",

linetype="longdash", lwd = 0.5) +

theme_bw(base_size = 12#, base_family = "Times" #修改字体

) +

theme(panel.grid=element_blank())

if (plot_mode == "advanced") {

p1 <- p1 +

geom_vline(xintercept = c(-logFCcut2, logFCcut2), color="grey40",

linetype="longdash", lwd = 0.5) +

geom_hline(yintercept = -log10(pvalCut2), color="grey40",

linetype="longdash", lwd = 0.5)

}

pdf(file="vol_target.pdf")

p1

dev.off()

#####heatmap

#Type=c(rep("Primary tumor",nonmetastasis),rep("Metastatic tumor",metastasis))

#Type=c(rep("Primary tumor",nonmetastasis),rep("Bone metastatic tumor",metastasis))

#Type=c(rep("Skeletal_muscle",nonmetastasis),rep("Rhabdomyosarcoma",metastasis))

#Type=c(rep("Normal_bone",nonmetastasis),rep("Chordoma",metastasis))

#Type=c(rep("Normal_bone",nonmetastasis),rep("Ewing_sarcoma",metastasis))

Type=c(rep("Normal blood",nonmetastasis),rep("SCI blood",metastasis))

#Type=c(rep("Normal",nonmetastasis),rep("OA",metastasis))

names(Type)=colnames(hmExp)

Type=as.data.frame(Type)

pdf(file="heatmap_target.pdf", width = 10,height =6)

pheatmap(hmExp,

annotation=Type,

color =colorRampPalette(c("#377EB8", "white", "#E41A1C"))(50),

cluster_cols =F,

show_colnames = F,

show_rownames = F,

fontsize = 12,

fontsize_row=6,

fontsize_col=10)

dev.off()

############cor

#########cor GSVA

corFilter=0.80

pvalueFilter=0.05

TF = read.table("Key_GeneExp.txt", row.names=1 ,header=T,sep="\t",check.names=F)

#uniSigExp = read.table("uniSigExp.txt", row.names=1 ,header=T,sep="\t",check.names=F)

#uniSigExp = read.table("uniSigExp_lasso.txt", row.names=1 ,header=T,sep="\t",check.names=F)

#uniSigExp= uniSigExp[,-1]

#uniSigExp= uniSigExp[,-1]

#gene1 = colnames(uniSigExp)

#TF= TF[gene1,]

immuneGene = gsva_es

sameSample=intersect(colnames(TF),colnames(immuneGene))

TF1=TF[,sameSample]

immuneGene1=immuneGene[,sameSample]

outTab=data.frame()

for(i in row.names(TF1)){

if(sd(TF1[i,])>1){

for(j in row.names(immuneGene1)){

x=as.numeric(TF1[i,])

y=as.numeric(immuneGene1[j,])

corT=cor.test(x,y)

cor=corT$estimate

pvalue=corT$p.value

if((cor>corFilter) & (pvalue<pvalueFilter)){

outTab=rbind(outTab,cbind(TF=i,immuneGene=j,cor,pvalue,Regulation="postive"))

}

if((cor< -corFilter) & (pvalue<pvalueFilter)){

outTab=rbind(outTab,cbind(TF=i,immuneGene=j,cor,pvalue,Regulation="negative"))

}

}

}

}

write.table(file="corResult_GSVA.txt",outTab,sep="\t",quote=F,row.names=F)

dim(outTab)

#########cor TF

corFilter=0.98

pvalueFilter=0.05

TF = read.table("Key_GeneExp.txt", row.names=1 ,header=T,sep="\t",check.names=F)

#uniSigExp = read.table("uniSigExp.txt", row.names=1 ,header=T,sep="\t",check.names=F)

#uniSigExp = read.table("uniSigExp_lasso.txt", row.names=1 ,header=T,sep="\t",check.names=F)

#uniSigExp= uniSigExp[,-1]

#uniSigExp= uniSigExp[,-1]

#gene1 = colnames(uniSigExp)

#TF= TF[gene1,]

immuneGene =read.table("Key_TF.txt", row.names=1 ,header=T,sep="\t",check.names=F)

sameSample=intersect(colnames(TF),colnames(immuneGene))

TF1=TF[,sameSample]

immuneGene1=immuneGene[,sameSample]

outTab=data.frame()

for(i in row.names(TF1)){

if(sd(TF1[i,])>1){

for(j in row.names(immuneGene1)){

x=as.numeric(TF1[i,])

y=as.numeric(immuneGene1[j,])

corT=cor.test(x,y)

cor=corT$estimate

pvalue=corT$p.value

if((cor>corFilter) & (pvalue<pvalueFilter)){

outTab=rbind(outTab,cbind(TF=i,immuneGene=j,cor,pvalue,Regulation="postive"))

}

if((cor< -corFilter) & (pvalue<pvalueFilter)){

outTab=rbind(outTab,cbind(TF=i,immuneGene=j,cor,pvalue,Regulation="negative"))

}

}

}

}

write.table(file="corResult_eRNA_TF.txt",outTab,sep="\t",quote=F,row.names=F)

dim(outTab)

########ssgasa

corFilter=0.90

pvalueFilter=0.05

TIMER = read.table("ssgseaOut.txt", row.names=1 ,header=T,sep="\t",check.names=F)

immuneGene = read.table("Key_GeneExp.txt", row.names=1 ,header=T,sep="\t",check.names=F)

#uniSigExp = read.table("uniSigExp.txt", row.names=1 ,header=T,sep="\t",check.names=F)

#uniSigExp = read.table("uniSigExp_lasso.txt", row.names=1 ,header=T,sep="\t",check.names=F)

#uniSigExp= uniSigExp[,-1]

#uniSigExp= uniSigExp[,-1]

#gene1 = colnames(uniSigExp)

#immuneGene= immuneGene[gene1,]

sameSample=intersect(colnames(TIMER),colnames(immuneGene))

TIMER=TIMER[,sameSample]

immuneGene1=immuneGene[,sameSample]

outTab=data.frame()

for(i in row.names(TIMER)){

if(sd(TIMER[i,])>0){

for(j in row.names(immuneGene1)){

x=as.numeric(TIMER[i,])

y=as.numeric(immuneGene1[j,])

corT=cor.test(x,y)

cor=corT$estimate

pvalue=corT$p.value

if((cor>corFilter) & (pvalue<pvalueFilter)){

outTab=rbind(outTab,cbind(TF=i,immuneGene=j,cor,pvalue,Regulation="postive"))

}

if((cor< -corFilter) & (pvalue<pvalueFilter)){

outTab=rbind(outTab,cbind(TF=i,immuneGene=j,cor,pvalue,Regulation="negative"))

}

}

}

}

write.table(file="corResult_ssGSEA.txt",outTab,sep="\t",quote=F,row.names=F)

dim(outTab)

##########immune cor

corFilter=0.80

pvalueFilter=0.05

TIMER = read.table("CIBERSORT.filter_trans.txt", row.names=1 ,header=T,sep="\t",check.names=F)

immuneGene = read.table("Key_GeneExp.txt", row.names=1 ,header=T,sep="\t",check.names=F)

#uniSigExp = read.table("uniSigExp.txt", row.names=1 ,header=T,sep="\t",check.names=F)

#uniSigExp = read.table("uniSigExp_lasso.txt", row.names=1 ,header=T,sep="\t",check.names=F)

#uniSigExp= uniSigExp[,-1]

#uniSigExp= uniSigExp[,-1]

#gene1 = colnames(uniSigExp)

#immuneGene= immuneGene[gene1,]

sameSample=intersect(colnames(TIMER),colnames(immuneGene))

TIMER=TIMER[,sameSample]

immuneGene1=immuneGene[,sameSample]

outTab=data.frame()

for(i in row.names(TIMER)){

if(sd(TIMER[i,])>0){

for(j in row.names(immuneGene1)){

x=as.numeric(TIMER[i,])

y=as.numeric(immuneGene1[j,])

corT=cor.test(x,y)

cor=corT$estimate

pvalue=corT$p.value

if((cor>corFilter) & (pvalue<pvalueFilter)){

outTab=rbind(outTab,cbind(TF=i,immuneGene=j,cor,pvalue,Regulation="postive"))

}

if((cor< -corFilter) & (pvalue<pvalueFilter)){

outTab=rbind(outTab,cbind(TF=i,immuneGene=j,cor,pvalue,Regulation="negative"))

}

}

}

}

write.table(file="corResult_CIBERSORT.txt",outTab,sep="\t",quote=F,row.names=F)

dim(outTab)

#########cor target

corFilter=0.95

pvalueFilter=0.05

TF = read.table("Key_GeneExp.txt", row.names=1 ,header=T,sep="\t",check.names=F)

#uniSigExp = read.table("uniSigExp.txt", row.names=1 ,header=T,sep="\t",check.names=F)

#uniSigExp = read.table("uniSigExp_lasso.txt", row.names=1 ,header=T,sep="\t",check.names=F)

#uniSigExp= uniSigExp[,-1]

#uniSigExp= uniSigExp[,-1]

#gene1 = colnames(uniSigExp)

#TF= TF[gene1,]

immuneGene =read.table("target_geneExp.txt", row.names=1 ,header=T,sep="\t",check.names=F)

sameSample=intersect(colnames(TF),colnames(immuneGene))

TF1=TF[,sameSample]

immuneGene1=immuneGene[,sameSample]

outTab=data.frame()

for(i in row.names(TF1)){

if(sd(TF1[i,])>0){

for(j in row.names(immuneGene1)){

x=as.numeric(TF1[i,])

y=as.numeric(immuneGene1[j,])

corT=cor.test(x,y)

cor=corT$estimate

pvalue=corT$p.value

if((cor>corFilter) & (pvalue<pvalueFilter)){

outTab=rbind(outTab,cbind(TF=i,immuneGene=j,cor,pvalue,Regulation="postive"))

}

if((cor< -corFilter) & (pvalue<pvalueFilter)){

outTab=rbind(outTab,cbind(TF=i,immuneGene=j,cor,pvalue,Regulation="negative"))

}

}

}

}

write.table(file="corResult_eRNA_target.txt",outTab,sep="\t",quote=F,row.names=F)

dim(outTab)

##########

#######cor_all

cor1 = read.table("corResult_eRNA_TF.txt", header=T,sep="\t",check.names=F)

cor2 = read.table("corResult_GSVA.txt", header=T,sep="\t",check.names=F)

cor3 = read.table("corResult_eRNA_target.txt", header=T,sep="\t",check.names=F)

cor5 = read.table("corResult_CIBERSORT.txt", header=T,sep="\t",check.names=F)

cor6 = read.table("corResult_ssGSEA.txt", header=T,sep="\t",check.names=F)

lnc = intersect(as.vector(cor1[,1]),as.vector(cor2[,1]))

lnc = intersect(lnc,as.vector(cor3[,1]))

lnc = intersect(lnc,as.vector(cor5[,2]))

lnc = intersect(lnc,as.vector(cor6[,2]))

prgs = read.table("PRGs.txt", header=F,sep="\t",check.names=F)

prgs = as.vector(prgs[,1])

lnc = intersect(lnc,prgs)

write.table(file="final_cor_sig.txt",lnc,sep="\t",quote=F,row.names = F)

cor7=cor1[which(cor1[,1]%in%lnc),]

cor8=cor2[which(cor2[,1]%in%lnc),]

cor9=cor3[which(cor3[,1]%in%lnc),]

cor11=cor5[which(cor5[,2]%in%lnc),]

cor12=cor6[which(cor6[,2]%in%lnc),]

cor_all=rbind(cor7,cor8,cor9,cor11,cor12)

dim(cor7)

dim(cor8)

dim(cor9)

dim(cor11)

dim(cor12)

dim(cor_all)

write.table(file="cor_all.txt",cor_all,sep="\t",quote=F,row.names=F)

##########

listgene = read.table("finallistgene.txt", header=F)

listpathway = read.table("finallistpathway.txt", header=F)

listimm= read.table("finallistimm.txt",sep="\t", header=F)

listssgsea= read.table("finallistssgsea.txt", header=F)

listgene= as.vector(listgene[,1])

listpathway= as.vector(listpathway[,1])

listimm= as.vector(listimm[,1])

listssgsea= as.vector(listssgsea[,1])

rt=read.table("symbol_TPM.txt",sep="\t",header=T,check.names=F)

rt <- as.matrix(rt)

rownames(rt)=rt[,1]

exp=rt[,2:ncol(rt)]

dimnames=list(rownames(exp),colnames(exp))

immuneGene=matrix(as.numeric(as.matrix(exp)),nrow=nrow(exp),dimnames=dimnames)

finalgene = immuneGene[listgene,]

finalpathway = gsva_es[listpathway,]

TIMER1 = read.table("CIBERSORT.filter_trans.txt", row.names=1 ,header=T,sep="\t",check.names=F)

TIMER2 = read.table("ssgseaOut.txt", row.names=1 ,header=T,sep="\t",check.names=F)

finalcell=TIMER1[listimm,]

finalssgeas=TIMER2[listssgsea,]

colnames(finalgene)= substring(colnames(finalgene),1,15)

colnames(finalpathway)= substring(colnames(finalpathway),1,15)

sameSample=intersect(colnames(finalgene),colnames(finalpathway))

sameSample=intersect(sameSample,colnames(finalcell))

sameSample=intersect(sameSample,colnames(finalssgeas))

finalgene= finalgene[,sameSample]

finalpathway= finalpathway[,sameSample]

finalcell= finalcell[,sameSample]

finalssgeas= finalssgeas[,sameSample]

finalall=rbind(finalgene,finalpathway, finalcell,finalssgeas)

pdf("corHeatmap_all.pdf",height=12,width=12)

ggcorrplot(corr=cor(t(finalall)),

type = "lower",

lab = T,lab_size = 1.5,tl.cex = 12)

dev.off()

########final_key_list

fdrFilter=0.05

logFCfilter=1

rt=read.table("diff.xls",sep="\t",header=T,check.names=F,row.names = 1)

diffExp=read.table("symbol_TPM.txt",sep="\t",header=T,check.names=F)

diffExp <- as.matrix(diffExp)

rownames(diffExp)=diffExp[,1]

exp=diffExp[,2:ncol(diffExp)]

dimnames=list(rownames(exp),colnames(exp))

diffExp=matrix(as.numeric(as.matrix(exp)),nrow=nrow(exp),dimnames=dimnames)

gene=read.table("finallistgene.txt",sep="\t",header=F)

immuneDiffAll=rt[intersect(gene[,1],row.names(rt)),]

immuneDiffGene=intersect(gene[,1],rownames(rt))

hmExp=diffExp[immuneDiffGene,]

immuneDiffResult=immuneDiffAll[immuneDiffGene,]

immuneDiffResult=rbind(ID=colnames(immuneDiffResult),immuneDiffResult)

write.table(immuneDiffResult,file="finallistgene_diff.xls",sep="\t",col.names=F,quote=F)

immuneGeneExp=rbind(ID=colnames(hmExp),hmExp)

write.table(immuneGeneExp,file="finallistgene_geneExp.txt",sep="\t",quote=F,col.names=F)

Type=read.table("clinical_initial.txt",sep="\t",header=T,check.names=F,row.names = 1)

#Type=c(rep("normal whole blood",nonmetastasis),rep("Septic shock blood",metastasis))

#Type=c(rep("non-metastasis",nonmetastasis),rep("metastasis",metastasis))

#Type=c(rep("primarytumor",nonmetastasis),rep("new_tumor_event",metastasis))

Type=as.data.frame(Type)

pdf(file="heatmap_finallistgene.pdf", width = 10,height =6)

pheatmap(hmExp,

annotation=Type,

color =colorRampPalette(c("#377EB8", "white", "#E41A1C"))(50),

cluster_cols =F,

show_colnames = F,

show_rownames = T,

fontsize = 12,

fontsize_row=8,

fontsize_col=10)

dev.off()

#########Cmap

library(xlsx)

library(tidyverse)

library(GEOquery)

library(plyr)

library(circlize)

library(ComplexHeatmap)

options(java.parameters = "-Xmx8000m")

Sys.setenv(LANGUAGE = "en") #显示英文报错信息

options(stringsAsFactors = FALSE) #禁止chr转成factor

#CMap要求输入基因是GPL96 platform ID的形式

setwd("F:\\TCGA_FPKM\\Cmap")

GPL96 <- getGEO("GPL96", destdir = getwd())

GPL96 <- Table(GPL96)[, c("ID", "Gene Symbol")]

pan_n <- 33

pancancertype <- c("ACC", "BLCA", "BRCA", "CESC", "CHOL", "COAD", "DLBC", "ESCA", "GBM", "HNSC",

"KICH", "KIRC", "KIRP", "LAML", "LGG", "LIHC", "LUAD", "LUSC", "MESO", "OV",

"PAAD", "PCPG", "PRAD", "READ", "SARC", "SKCM", "STAD", "TGCT", "THCA", "THYM",

"UCEC", "UCS", "UVM")

diflist <- list()

# 读取数据 拷贝finallistgene_diff.xls 处理pandif_mRNAsi.xlsx

for (i in 1:pan_n){

print(paste0("Load ", pancancertype[i], " differentially expressed gene data"))

difdata <- openxlsx::read.xlsx("pandif_mRNAsi.xlsx", sheet = i, colNames = TRUE) %>%

merge(GPL96, ., by.x = "Gene Symbol", by.y = "GeneSymbol")

difdata$logFC <- as.numeric(difdata$logFC)

difdata <- difdata[order(difdata$logFC, decreasing = T), ]

diflist[[i]] <- difdata

}

names(diflist) <- pancancertype

# 输出grp文件(one gene per line)

if (!file.exists("./grpfile")){

dir.create("./grpfile")

}

for (i in 1:pan_n){

print(paste0(pancancertype[i], " processing"))

tmpdata <- diflist[[i]]

tmp_updata <- tmpdata[tmpdata$logFC > 0, ]

tmp_downdata <- tmpdata[tmpdata$logFC < 0, ]

# 选择差异最大的前500个基因

if (nrow(tmp_updata) > 500){

top500up <- tmp_updata[, "ID"][1:500]

} else {top500up <- tmp_updata[, "ID"]}

if (nrow(tmp_downdata) > 500){

top500down <- rev(tmp_downdata[, "ID"])[1:500]

} else {top500down <- rev(tmp_downdata[, "ID"])}

# 写成grp格式文件

write.table(top500up, paste0("grpfile/", pancancertype[i], "_up500.grp"),

row.names = F, sep = "\t", quote = F, col.names = F)

write.table(top500down, paste0("grpfile/", pancancertype[i], "_down500.grp"),

row.names = F, sep = "\t", quote = F, col.names = F)

}

###########产生差异基因的.grp文件

difdata <- read.table("finallistgene_diff.xls", header=T,sep="\t",check.names=F) %>%

merge(GPL96, ., by.x = "Gene Symbol", by.y = "ID")

difdata$logFC <- as.numeric(difdata$logFC)

difdata <- difdata[order(difdata$logFC, decreasing = T), ]

tmpdata <- difdata

tmp_updata <- tmpdata[tmpdata$logFC > 0, ]

tmp_downdata <- tmpdata[tmpdata$logFC < 0, ]

# 选择差异最大的前500个基因

if (nrow(tmp_updata) > 500){

top500up <- tmp_updata[, "ID"][1:500]

} else {top500up <- tmp_updata[, "ID"]}

if (nrow(tmp_downdata) > 500){

top500down <- rev(tmp_downdata[, "ID"])[1:500]

} else {top500down <- rev(tmp_downdata[, "ID"])}

# 写成grp格式文件

write.table(top500up, "grpfile_up500.grp",

row.names = F, sep = "\t", quote = F, col.names = F)

write.table(top500down, "grpfile_down500.grp",

row.names = F, sep = "\t", quote = F, col.names = F)

#####获得CMap_result.xls。

# 按操作文档得到的结果

ACC_CMap_result <- xlsx::read.xlsx("CMap_result.xls", sheetIndex = 1, header = T)

ACC_CMap_result$p <- as.numeric(ACC_CMap_result$p)

# p < 0.05 筛选具有统计学意义的化合物

ACC_CMap_sig <- na.omit(ACC_CMap_result)

ACC_CMap_sig <- ACC_CMap_sig[ACC_CMap_sig$p < 0.01, ]

dim(ACC_CMap_sig)

ACC_CMap_sig= cbind(rownames(ACC_CMap_sig),ACC_CMap_sig)

write.table(file="Connectivity_Map_Output.txt",ACC_CMap_sig,sep="\t",quote=F,row.names=F)

drug2 = read.table("Connectivity_Map_Output.txt",sep="\t",header=T,check.names=F,row.names = 1)

drug2$cmap.name

p1<-ggplot(drug2,aes(cmap.name,enrichment,size=specificity))+

geom_point(shape=21,aes(fill=p),position =position_dodge(0))+

theme_minimal()+xlab(NULL)+ylab(NULL)+

scale_size_continuous(range=c(1,8))+

scale_fill_gradientn(colours=c("#2381B3","#F0E366"),guide="legend")+

theme(axis.text.x=element_text(angle= 30),legend.position = "bottom",legend.box = "vertical",panel.grid.major =element_blank() )

ggsave(file="Only.pdf",height=6,width = 8)

############ATAC-seq

setwd("F:\\TCGA-ATAC-seq_bigwig\\ATAC-seq")

library(chromVAR)

library(Biostrings)

library(BSgenome.Hsapiens.UCSC.hg38)

library(ChIPseeker)

library(TxDb.Hsapiens.UCSC.hg38.knownGene)

library(clusterProfiler)

library(org.Hs.eg.db)

library(ggplot2)

library(karyoploteR)

library(limma)

library(GOplot)

library(chromVAR)

library(Biostrings)

library(BSgenome.Hsapiens.UCSC.hg38)

########perl tcgaATAC.prepareData.pl BLCA_log2norm.txt

rt=read.table("peak.bed",sep="\t",header=F)

colnames(rt)=c("chr","start","end","peak","value")

data.points=makeGRangesFromDataFrame(rt)

mcols(data.points) <- data.frame( y=rt[,5])

pdf(file="coverage.pdf",width=10,height=7)

kp <- plotKaryotype("hg38", plot.type=1)

kpDataBackground(kp, data.panel=1)

kpArea(kp, data=data.points,border="#329E3F",ymin=0,ymax=100)

kpAddBaseNumbers(kp, tick.dist=10000000, minor.tick.dist=1000000)

dev.off()

#########pie

inputFile="peak.bed"

library(ChIPseeker)

library(TxDb.Hsapiens.UCSC.hg38.knownGene)

library(org.Hs.eg.db)

library(clusterProfiler)

txdb <- TxDb.Hsapiens.UCSC.hg38.knownGene

peakAnno <- annotatePeak(inputFile, tssRegion=c(-3000, 3000),

TxDb=txdb, annoDb="org.Hs.eg.db")

pdf("pie.pdf",height=7,width=7)

plotAnnoPie(peakAnno)

dev.off()

pdf("bar.pdf",height=6,width=14)

plotAnnoBar(peakAnno)

dev.off()

pdf("vennpie.pdf",height=7,width=8)

vennpie(peakAnno)

dev.off()

pdf("upsetplot1.pdf",height=10,width=12)

upsetplot(peakAnno)

dev.off()

pdf("upsetplot2.pdf",height=10,width=12)

upsetplot(peakAnno, vennpie=TRUE)

dev.off()

pdf("DistToTSS.pdf",height=3,width=10)

plotDistToTSS(peakAnno,

title="Distribution of binding loci\relative to TSS")

dev.off()

########TagMatrix

inputFile="peak.bed"

txdb <- TxDb.Hsapiens.UCSC.hg38.knownGene

peak <- readPeakFile(inputFile)

promoter <- getPromoters(TxDb=txdb, upstream=3000, downstream=3000)

tagMatrix <- getTagMatrix(peak, windows=promoter)

pdf("tagHeatmap.pdf",height=12,width=12)

tagHeatmap(tagMatrix, xlim=c(-3000, 3000), color="#FEB24C")

dev.off()

pdf("aveProfile.pdf",height=6,width=10)

plotAvgProf(tagMatrix, xlim=c(-3000, 3000),

xlab="Genomic Region (5'->3')", ylab = "Read Count Frequency")

dev.off()

pdf("aveProfile.conf.pdf",height=6,width=10)

plotAvgProf(tagMatrix, xlim=c(-3000, 3000),conf = 0.95, resample = 1000,

xlab="Genomic Region (5'->3')", ylab = "Read Count Frequency")

dev.off()

#######GO_KEGG

txdb <- TxDb.Hsapiens.UCSC.hg38.knownGene

peak <- readPeakFile(inputFile)

peakAnno <- annotatePeak(inputFile, tssRegion=c(-3000, 3000),

TxDb=txdb, annoDb="org.Hs.eg.db")

gene=as.data.frame(peakAnno)$geneId

#GO

kk <- enrichGO(gene = gene,

OrgDb = org.Hs.eg.db,

pvalueCutoff =0.05,

qvalueCutoff = 0.05,

ont="all",

readable =T)

write.table(kk,file="GO_ATAC.txt",sep="\t",quote=F,row.names = F)

pdf(file="GO_barplot_ATAC.pdf",width = 12,height = 7)

barplot(kk, drop = TRUE, showCategory =10,split="ONTOLOGY") + facet_grid(ONTOLOGY~., scale='free')

dev.off()

pdf(file="GO_dotplot_ATAC.pdf",width = 12,height = 7)

dotplot(kk,showCategory = 10,split="ONTOLOGY") + facet_grid(ONTOLOGY~., scale='free')

dev.off()

#KEGG

kk <- enrichKEGG(gene = gene, organism = "hsa", pvalueCutoff =1, qvalueCutoff =1)

KEGG=as.data.frame(kk)

write.table(KEGG,file="KEGG_ATAC.txt",sep="\t",quote=F,row.names = F)

pdf(file="KEGG_barplot_ATAC.pdf",width = 12,height = 7)

barplot(kk, drop = TRUE, showCategory = 20,)

dev.off()

pdf(file="KEGG_dotplot_ATAC.pdf",width = 12,height = 7)

dotplot(kk, showCategory = 20, orderBy = "GeneRatio")

dev.off()

##########共表达分析

setwd("F:\\TCGA-ATAC-seq_bigwig\\ATAC-seq")

peak = read.table("peakMatrix.txt", row.names=1 ,header=T,sep="\t",check.names=F)

colnames(peak)= substring(colnames(peak),1,15)

rt=read.table("Group_symbol_FPKM.txt",sep="\t",header=T,check.names=F) #??ȡ?ļ?

#rt=read.table("symbol.txt",sep="\t",header=T,check.names=F)

rt=as.matrix(rt)

rownames(rt)=rt[,1]

exp=rt[,2:ncol(rt)]

dimnames=list(rownames(exp),colnames(exp))

RNA=matrix(as.numeric(as.matrix(exp)),nrow=nrow(exp),dimnames=dimnames)

RNA=avereps(RNA)

RNA=RNA[rowMeans(RNA)>0,]

colnames(peak)=gsub("(.*?)\\-(.*?)\\-(.*?)\\-(.*?)\\-.*","\\1\\-\\2\\-\\3\\-\\4",colnames(peak))

rownames(peak)=paste(rownames(peak),"peak",sep="|")

rownames(RNA)=paste(rownames(RNA),"mRNA",sep="|")

sameSample=intersect(colnames(peak),colnames(RNA))

merge=rbind(id=sameSample,peak[,sameSample],RNA[,sameSample])

write.table(merge,file="merge.txt",sep="\t",quote=F,col.names=F)

#######cor

inputFile="merge.txt" #?????ļ?

gene="PDE9A|mRNA" #??????lncRNA????

corFilter=0.50 #????ϵ??????ֵ

pFilter=0.05 #ͳ??ѧpֵ????ֵ

picDir="cor_PDE9A_picture"

dir.create(picDir)

library(limma)

rt=read.table(inputFile,sep="\t",header=T,check.names=F)

rt=as.matrix(rt)

rownames(rt)=rt[,1]

exp=rt[,2:ncol(rt)]

dimnames=list(rownames(exp),colnames(exp))

data=matrix(as.numeric(as.matrix(exp)),nrow=nrow(exp),dimnames=dimnames)

data=avereps(data)

rt=data[rowMeans(data)>0,]

y=log2(as.numeric(rt[gene,])+1)

gene1=unlist(strsplit(gene,"\\|",))[1]

outputFile=paste(gene1,".cor.xls",sep="")

outTab=data.frame()

for(j in rownames(rt)){

x=as.numeric(rt[j,])

gene2=unlist(strsplit(j,"\\|",))[1]

gene2Type=unlist(strsplit(j,"\\|",))[2]

if(gene2Type=="peak"){

corT=cor.test(x,y)

gene1Name=unlist(strsplit(gene1,"\\|",))[1]

gene2Name=gsub(":", "-", gene2)

z=lm(y~x)

cor=corT$estimate

cor=round(cor,3)

pvalue=corT$p.value

if(pvalue<0.001){

pval=signif(pvalue,4)

pval=format(pval, scientific = TRUE)

}else{

pval=round(pvalue,3)}

#??????????ͼƬ

if((abs(cor)>corFilter) & (pvalue<pFilter)){

pdfFile=paste(gene1Name,"_",gene2Name,".cor.pdf",sep="")

outPdf=paste(picDir,pdfFile,sep="\\")

pdf(file=outPdf,width =5,height = 5)

plot(x,y, type="p",pch=16,col="blue",main=paste("Cor=",cor," (p-value=",pval,")",sep=""),

cex=1, cex.lab=1, cex.main=1,cex.axis=1,

xlab=gene2,

ylab=paste(gene1,"expression") )

lines(x,fitted(z),col=2)

dev.off()

outTab=rbind(outTab,cbind(gene1,gene2,cor,pvalue))

}

}

}

write.table(file=outputFile,outTab,sep="\t",quote=F,row.names=F)

#######cor

inputFile="merge.txt" #?????ļ?

gene="TSPO|mRNA" #??????lncRNA????

corFilter=0.50 #????ϵ??????ֵ

pFilter=0.05 #ͳ??ѧpֵ????ֵ

picDir="cor_TSPO_picture"

dir.create(picDir)

library(limma)

rt=read.table(inputFile,sep="\t",header=T,check.names=F)

rt=as.matrix(rt)

rownames(rt)=rt[,1]

exp=rt[,2:ncol(rt)]

dimnames=list(rownames(exp),colnames(exp))

data=matrix(as.numeric(as.matrix(exp)),nrow=nrow(exp),dimnames=dimnames)

data=avereps(data)

rt=data[rowMeans(data)>0,]

y=log2(as.numeric(rt[gene,])+1)

gene1=unlist(strsplit(gene,"\\|",))[1]

outputFile=paste(gene1,".cor.xls",sep="")

outTab=data.frame()

for(j in rownames(rt)){

x=as.numeric(rt[j,])

gene2=unlist(strsplit(j,"\\|",))[1]

gene2Type=unlist(strsplit(j,"\\|",))[2]

if(gene2Type=="peak"){

corT=cor.test(x,y)

gene1Name=unlist(strsplit(gene1,"\\|",))[1]

gene2Name=gsub(":", "-", gene2)

z=lm(y~x)

cor=corT$estimate

cor=round(cor,3)

pvalue=corT$p.value

if(pvalue<0.001){

pval=signif(pvalue,4)

pval=format(pval, scientific = TRUE)

}else{

pval=round(pvalue,3)}

#??????????ͼƬ

if((abs(cor)>corFilter) & (pvalue<pFilter)){

pdfFile=paste(gene1Name,"_",gene2Name,".cor.pdf",sep="")

outPdf=paste(picDir,pdfFile,sep="\\")

pdf(file=outPdf,width =5,height = 5)

plot(x,y, type="p",pch=16,col="blue",main=paste("Cor=",cor," (p-value=",pval,")",sep=""),

cex=1, cex.lab=1, cex.main=1,cex.axis=1,

xlab=gene2,

ylab=paste(gene1,"expression") )

lines(x,fitted(z),col=2)

dev.off()

outTab=rbind(outTab,cbind(gene1,gene2,cor,pvalue))

}

}

}

write.table(file=outputFile,outTab,sep="\t",quote=F,row.names=F)

#######cor

inputFile="merge.txt" #?????ļ?

gene="RNF43|mRNA" #??????lncRNA????

corFilter=0.50 #????ϵ??????ֵ

pFilter=0.05 #ͳ??ѧpֵ????ֵ

picDir="cor_RNF43_picture"

dir.create(picDir)

library(limma)

rt=read.table(inputFile,sep="\t",header=T,check.names=F)

rt=as.matrix(rt)

rownames(rt)=rt[,1]

exp=rt[,2:ncol(rt)]

dimnames=list(rownames(exp),colnames(exp))

data=matrix(as.numeric(as.matrix(exp)),nrow=nrow(exp),dimnames=dimnames)

data=avereps(data)

rt=data[rowMeans(data)>0,]

y=log2(as.numeric(rt[gene,])+1)

gene1=unlist(strsplit(gene,"\\|",))[1]

outputFile=paste(gene1,".cor.xls",sep="")

outTab=data.frame()

for(j in rownames(rt)){

x=as.numeric(rt[j,])

gene2=unlist(strsplit(j,"\\|",))[1]

gene2Type=unlist(strsplit(j,"\\|",))[2]

if(gene2Type=="peak"){

corT=cor.test(x,y)

gene1Name=unlist(strsplit(gene1,"\\|",))[1]

gene2Name=gsub(":", "-", gene2)

z=lm(y~x)

cor=corT$estimate

cor=round(cor,3)

pvalue=corT$p.value

if(pvalue<0.001){

pval=signif(pvalue,4)

pval=format(pval, scientific = TRUE)

}else{

pval=round(pvalue,3)}

#??????????ͼƬ

if((abs(cor)>corFilter) & (pvalue<pFilter)){

pdfFile=paste(gene1Name,"_",gene2Name,".cor.pdf",sep="")

outPdf=paste(picDir,pdfFile,sep="\\")

pdf(file=outPdf,width =5,height = 5)

plot(x,y, type="p",pch=16,col="blue",main=paste("Cor=",cor," (p-value=",pval,")",sep=""),

cex=1, cex.lab=1, cex.main=1,cex.axis=1,

xlab=gene2,

ylab=paste(gene1,"expression") )

lines(x,fitted(z),col=2)

dev.off()

outTab=rbind(outTab,cbind(gene1,gene2,cor,pvalue))

}

}

}

write.table(file=outputFile,outTab,sep="\t",quote=F,row.names=F)

#######cor

inputFile="merge.txt" #?????ļ?

gene="HTRA1|mRNA" #??????lncRNA????

corFilter=0.50 #????ϵ??????ֵ

pFilter=0.05 #ͳ??ѧpֵ????ֵ

picDir="cor_HTRA1_picture"

dir.create(picDir)

library(limma)

rt=read.table(inputFile,sep="\t",header=T,check.names=F)

rt=as.matrix(rt)

rownames(rt)=rt[,1]

exp=rt[,2:ncol(rt)]

dimnames=list(rownames(exp),colnames(exp))

data=matrix(as.numeric(as.matrix(exp)),nrow=nrow(exp),dimnames=dimnames)

data=avereps(data)

rt=data[rowMeans(data)>0,]

y=log2(as.numeric(rt[gene,])+1)

gene1=unlist(strsplit(gene,"\\|",))[1]

outputFile=paste(gene1,".cor.xls",sep="")

outTab=data.frame()

for(j in rownames(rt)){

x=as.numeric(rt[j,])

gene2=unlist(strsplit(j,"\\|",))[1]

gene2Type=unlist(strsplit(j,"\\|",))[2]

if(gene2Type=="peak"){

corT=cor.test(x,y)

gene1Name=unlist(strsplit(gene1,"\\|",))[1]

gene2Name=gsub(":", "-", gene2)

z=lm(y~x)

cor=corT$estimate

cor=round(cor,3)

pvalue=corT$p.value

if(pvalue<0.001){

pval=signif(pvalue,4)

pval=format(pval, scientific = TRUE)

}else{

pval=round(pvalue,3)}

#??????????ͼƬ

if((abs(cor)>corFilter) & (pvalue<pFilter)){

pdfFile=paste(gene1Name,"_",gene2Name,".cor.pdf",sep="")

outPdf=paste(picDir,pdfFile,sep="\\")

pdf(file=outPdf,width =5,height = 5)

plot(x,y, type="p",pch=16,col="blue",main=paste("Cor=",cor," (p-value=",pval,")",sep=""),

cex=1, cex.lab=1, cex.main=1,cex.axis=1,

xlab=gene2,

ylab=paste(gene1,"expression") )

lines(x,fitted(z),col=2)

dev.off()

outTab=rbind(outTab,cbind(gene1,gene2,cor,pvalue))

}

}

}

write.table(file=outputFile,outTab,sep="\t",quote=F,row.names=F)

#######cor

inputFile="merge.txt" #?????ļ?

gene="PRKD1|mRNA" #??????lncRNA????

corFilter=0.50 #????ϵ??????ֵ

pFilter=0.05 #ͳ??ѧpֵ????ֵ

picDir="cor_PRKD1_picture"

dir.create(picDir)

library(limma)

rt=read.table(inputFile,sep="\t",header=T,check.names=F)

rt=as.matrix(rt)

rownames(rt)=rt[,1]

exp=rt[,2:ncol(rt)]

dimnames=list(rownames(exp),colnames(exp))

data=matrix(as.numeric(as.matrix(exp)),nrow=nrow(exp),dimnames=dimnames)

data=avereps(data)

rt=data[rowMeans(data)>0,]

y=log2(as.numeric(rt[gene,])+1)

gene1=unlist(strsplit(gene,"\\|",))[1]

outputFile=paste(gene1,".cor.xls",sep="")

outTab=data.frame()

for(j in rownames(rt)){

x=as.numeric(rt[j,])

gene2=unlist(strsplit(j,"\\|",))[1]

gene2Type=unlist(strsplit(j,"\\|",))[2]

if(gene2Type=="peak"){

corT=cor.test(x,y)

gene1Name=unlist(strsplit(gene1,"\\|",))[1]

gene2Name=gsub(":", "-", gene2)

z=lm(y~x)

cor=corT$estimate

cor=round(cor,3)

pvalue=corT$p.value

if(pvalue<0.001){

pval=signif(pvalue,4)

pval=format(pval, scientific = TRUE)

}else{

pval=round(pvalue,3)}

#??????????ͼƬ

if((abs(cor)>corFilter) & (pvalue<pFilter)){

pdfFile=paste(gene1Name,"_",gene2Name,".cor.pdf",sep="")

outPdf=paste(picDir,pdfFile,sep="\\")

pdf(file=outPdf,width =5,height = 5)

plot(x,y, type="p",pch=16,col="blue",main=paste("Cor=",cor," (p-value=",pval,")",sep=""),

cex=1, cex.lab=1, cex.main=1,cex.axis=1,

xlab=gene2,

ylab=paste(gene1,"expression") )

lines(x,fitted(z),col=2)

dev.off()

outTab=rbind(outTab,cbind(gene1,gene2,cor,pvalue))

}

}

}

write.table(file=outputFile,outTab,sep="\t",quote=F,row.names=F)

#######cor

inputFile="merge.txt" #?????ļ?

gene="SH2D4A|mRNA" #??????lncRNA????

corFilter=0.50 #????ϵ??????ֵ

pFilter=0.05 #ͳ??ѧpֵ????ֵ

picDir="cor_SH2D4A_picture"

dir.create(picDir)

library(limma)

rt=read.table(inputFile,sep="\t",header=T,check.names=F)

rt=as.matrix(rt)

rownames(rt)=rt[,1]

exp=rt[,2:ncol(rt)]

dimnames=list(rownames(exp),colnames(exp))

data=matrix(as.numeric(as.matrix(exp)),nrow=nrow(exp),dimnames=dimnames)

data=avereps(data)

rt=data[rowMeans(data)>0,]

y=log2(as.numeric(rt[gene,])+1)

gene1=unlist(strsplit(gene,"\\|",))[1]

outputFile=paste(gene1,".cor.xls",sep="")

outTab=data.frame()

for(j in rownames(rt)){

x=as.numeric(rt[j,])

gene2=unlist(strsplit(j,"\\|",))[1]

gene2Type=unlist(strsplit(j,"\\|",))[2]

if(gene2Type=="peak"){

corT=cor.test(x,y)

gene1Name=unlist(strsplit(gene1,"\\|",))[1]

gene2Name=gsub(":", "-", gene2)

z=lm(y~x)

cor=corT$estimate

cor=round(cor,3)

pvalue=corT$p.value

if(pvalue<0.001){

pval=signif(pvalue,4)

pval=format(pval, scientific = TRUE)

}else{

pval=round(pvalue,3)}

#??????????ͼƬ

if((abs(cor)>corFilter) & (pvalue<pFilter)){

pdfFile=paste(gene1Name,"_",gene2Name,".cor.pdf",sep="")

outPdf=paste(picDir,pdfFile,sep="\\")

pdf(file=outPdf,width =5,height = 5)

plot(x,y, type="p",pch=16,col="blue",main=paste("Cor=",cor," (p-value=",pval,")",sep=""),

cex=1, cex.lab=1, cex.main=1,cex.axis=1,

xlab=gene2,

ylab=paste(gene1,"expression") )

lines(x,fitted(z),col=2)

dev.off()

outTab=rbind(outTab,cbind(gene1,gene2,cor,pvalue))

}

}

}

write.table(file=outputFile,outTab,sep="\t",quote=F,row.names=F)

#######cor

inputFile="merge.txt" #?????ļ?

gene="AK2|mRNA" #??????lncRNA????

corFilter=0.50 #????ϵ??????ֵ

pFilter=0.05 #ͳ??ѧpֵ????ֵ

picDir="cor_AK2_picture"

dir.create(picDir)

library(limma)

rt=read.table(inputFile,sep="\t",header=T,check.names=F)

rt=as.matrix(rt)

rownames(rt)=rt[,1]

exp=rt[,2:ncol(rt)]

dimnames=list(rownames(exp),colnames(exp))

data=matrix(as.numeric(as.matrix(exp)),nrow=nrow(exp),dimnames=dimnames)

data=avereps(data)

rt=data[rowMeans(data)>0,]

y=log2(as.numeric(rt[gene,])+1)

gene1=unlist(strsplit(gene,"\\|",))[1]

outputFile=paste(gene1,".cor.xls",sep="")

outTab=data.frame()

for(j in rownames(rt)){

x=as.numeric(rt[j,])

gene2=unlist(strsplit(j,"\\|",))[1]

gene2Type=unlist(strsplit(j,"\\|",))[2]

if(gene2Type=="peak"){

corT=cor.test(x,y)

gene1Name=unlist(strsplit(gene1,"\\|",))[1]

gene2Name=gsub(":", "-", gene2)

z=lm(y~x)

cor=corT$estimate

cor=round(cor,3)

pvalue=corT$p.value

if(pvalue<0.001){

pval=signif(pvalue,4)

pval=format(pval, scientific = TRUE)

}else{

pval=round(pvalue,3)}

#??????????ͼƬ

if((abs(cor)>corFilter) & (pvalue<pFilter)){

pdfFile=paste(gene1Name,"_",gene2Name,".cor.pdf",sep="")

outPdf=paste(picDir,pdfFile,sep="\\")

pdf(file=outPdf,width =5,height = 5)

plot(x,y, type="p",pch=16,col="blue",main=paste("Cor=",cor," (p-value=",pval,")",sep=""),

cex=1, cex.lab=1, cex.main=1,cex.axis=1,

xlab=gene2,

ylab=paste(gene1,"expression") )

lines(x,fitted(z),col=2)

dev.off()

outTab=rbind(outTab,cbind(gene1,gene2,cor,pvalue))

}

}

}

write.table(file=outputFile,outTab,sep="\t",quote=F,row.names=F)

#######cor

inputFile="merge.txt" #?????ļ?

gene="AFTPH|mRNA" #??????lncRNA????

corFilter=0.50 #????ϵ??????ֵ

pFilter=0.05 #ͳ??ѧpֵ????ֵ

picDir="cor_AFTPH_picture"

dir.create(picDir)

library(limma)

rt=read.table(inputFile,sep="\t",header=T,check.names=F)

rt=as.matrix(rt)

rownames(rt)=rt[,1]

exp=rt[,2:ncol(rt)]

dimnames=list(rownames(exp),colnames(exp))

data=matrix(as.numeric(as.matrix(exp)),nrow=nrow(exp),dimnames=dimnames)

data=avereps(data)

rt=data[rowMeans(data)>0,]

y=log2(as.numeric(rt[gene,])+1)

gene1=unlist(strsplit(gene,"\\|",))[1]

outputFile=paste(gene1,".cor.xls",sep="")

outTab=data.frame()

for(j in rownames(rt)){

x=as.numeric(rt[j,])

gene2=unlist(strsplit(j,"\\|",))[1]

gene2Type=unlist(strsplit(j,"\\|",))[2]

if(gene2Type=="peak"){

corT=cor.test(x,y)

gene1Name=unlist(strsplit(gene1,"\\|",))[1]

gene2Name=gsub(":", "-", gene2)

z=lm(y~x)

cor=corT$estimate

cor=round(cor,3)

pvalue=corT$p.value

if(pvalue<0.001){

pval=signif(pvalue,4)

pval=format(pval, scientific = TRUE)

}else{

pval=round(pvalue,3)}

#??????????ͼƬ

if((abs(cor)>corFilter) & (pvalue<pFilter)){

pdfFile=paste(gene1Name,"_",gene2Name,".cor.pdf",sep="")

outPdf=paste(picDir,pdfFile,sep="\\")

pdf(file=outPdf,width =5,height = 5)

plot(x,y, type="p",pch=16,col="blue",main=paste("Cor=",cor," (p-value=",pval,")",sep=""),

cex=1, cex.lab=1, cex.main=1,cex.axis=1,

xlab=gene2,

ylab=paste(gene1,"expression") )

lines(x,fitted(z),col=2)

dev.off()

outTab=rbind(outTab,cbind(gene1,gene2,cor,pvalue))

}

}

}

write.table(file=outputFile,outTab,sep="\t",quote=F,row.names=F)

#######cor

inputFile="merge.txt" #?????ļ?

gene="TFDP2|mRNA" #??????lncRNA????

corFilter=0.50 #????ϵ??????ֵ

pFilter=0.05 #ͳ??ѧpֵ????ֵ

picDir="cor_TFDP2_picture"

dir.create(picDir)

library(limma)

rt=read.table(inputFile,sep="\t",header=T,check.names=F)

rt=as.matrix(rt)

rownames(rt)=rt[,1]

exp=rt[,2:ncol(rt)]

dimnames=list(rownames(exp),colnames(exp))

data=matrix(as.numeric(as.matrix(exp)),nrow=nrow(exp),dimnames=dimnames)

data=avereps(data)

rt=data[rowMeans(data)>0,]

y=log2(as.numeric(rt[gene,])+1)

gene1=unlist(strsplit(gene,"\\|",))[1]

outputFile=paste(gene1,".cor.xls",sep="")

outTab=data.frame()

for(j in rownames(rt)){

x=as.numeric(rt[j,])

gene2=unlist(strsplit(j,"\\|",))[1]

gene2Type=unlist(strsplit(j,"\\|",))[2]

if(gene2Type=="peak"){

corT=cor.test(x,y)

gene1Name=unlist(strsplit(gene1,"\\|",))[1]

gene2Name=gsub(":", "-", gene2)

z=lm(y~x)

cor=corT$estimate

cor=round(cor,3)

pvalue=corT$p.value

if(pvalue<0.001){

pval=signif(pvalue,4)

pval=format(pval, scientific = TRUE)

}else{

pval=round(pvalue,3)}

#??????????ͼƬ

if((abs(cor)>corFilter) & (pvalue<pFilter)){

pdfFile=paste(gene1Name,"_",gene2Name,".cor.pdf",sep="")

outPdf=paste(picDir,pdfFile,sep="\\")

pdf(file=outPdf,width =5,height = 5)

plot(x,y, type="p",pch=16,col="blue",main=paste("Cor=",cor," (p-value=",pval,")",sep=""),

cex=1, cex.lab=1, cex.main=1,cex.axis=1,

xlab=gene2,

ylab=paste(gene1,"expression") )

lines(x,fitted(z),col=2)

dev.off()

outTab=rbind(outTab,cbind(gene1,gene2,cor,pvalue))

}

}

}

write.table(file=outputFile,outTab,sep="\t",quote=F,row.names=F)

#########

inputFile="corPeak.txt"

library(ChIPseeker)

library(TxDb.Hsapiens.UCSC.hg38.knownGene)

library(org.Hs.eg.db)

library(clusterProfiler)

txdb <- TxDb.Hsapiens.UCSC.hg38.knownGene

peakAnno <- annotatePeak(inputFile, tssRegion=c(-3000, 3000),

TxDb=txdb, annoDb="org.Hs.eg.db")

write.table(as.data.frame(peakAnno),file="corPeakAnn.xls",sep="\t",quote=F,row.names = F)

############PEAK

setwd("F:\\TCGA-ATAC-seq_bigwig\\GSE139099")

library(data.table)

library(Gviz)

library(RColorBrewer)

library(TxDb.Hsapiens.UCSC.hg38.knownGene)

txdb_hg38 <- TxDb.Hsapiens.UCSC.hg38.knownGene

grt <- GeneRegionTrack(txdb_hg38, genome="hg38",showId=TRUE, geneSymbol=TRUE, name="UCSC")

library(org.Hs.eg.db)

z <- mapIds(org.Hs.eg.db, gene(grt), "SYMBOL", "ENTREZID", multiVals = "first")

zz <- sapply(z, is.null)

z[zz] <- gene(grt)[zz]

gr <- ranges(grt)

mcols(gr)$symbol <- z

grt@range <- gr

# 输入数据

bwInfo<-read.table("easy_input.txt",header=F,row.names=1,as.is=T)

head(bwInfo)

gloci<-read.table("loci_CPT1A.bed",header=F,as.is=T)

head(gloci)

#可调整的参数

genefold<-as.numeric("1.5")#放大、缩小展示的范围

# 展示的基因组范围

colnames(gloci)<-c("chr","start","end","strand")

chr<-gloci[rownames(gloci),]$chr

gloci$width<-with(gloci,end-start)

startpoint<-gloci[rownames(gloci),]$start-genefold*gloci[rownames(gloci),]$width

endpoint<-gloci[rownames(gloci),]$end+genefold*gloci[rownames(gloci),]$width

#下面将scale等track写入tracklist

tracklist<-list()

#写入chromosome

itrack <- IdeogramTrack(genome = "hg38", chromosome = chr,outline=T)

tracklist[["itrack"]]<-itrack

#写入比例尺

scalebar <- GenomeAxisTrack(scale=0.25,col="black",fontcolor="black",name="Scale",labelPos="above",showTitle=TRUE)

tracklist[["scalebar"]]<-scalebar

#写入基因组位置

axisTrack <- GenomeAxisTrack(labelPos="above",col="black",fontcolor="black",name=paste(chr,":",sep=""),exponent=0,showTitle=TRUE)

tracklist[["axisTrack"]]<-axisTrack

#写入bigwig

#配色

colpal<-rep(brewer.pal(12,"Paired"),20)

coldf<-data.frame(col=colpal[1:nrow(bwInfo)],row.names = rownames(bwInfo),stringsAsFactors = F)

for(index in rownames(bwInfo)){

bgFile<-file.path("F:\\TCGA-ATAC-seq_bigwig\\GSE139099",paste(index,".bw",sep=""))

tracklist[[index]]<-DataTrack(range = bgFile,genome="hg38",type="histogram",

name=chartr("_","\n",bwInfo[index,]),

col.histogram=coldf[index,])#每个track颜色不同才好看

}

#写入基因结构

tracklist[["grt"]]<-grt

#输出pdf文件

pdf("loci_CPT1A.pdf",height=20,width=10)

plotTracks(tracklist, from = startpoint, to = endpoint,

chromosome=chr,background.panel = "white", background.title = "white",

col.title="black",col.axis="black",ylim=c(0,50),

rot.title=0,cex.title=0.9,margin=38,title.width=1.5,collapseTranscripts = "longest")

#collapseTranscripts = "longest")#同一个基因的多个transcript压缩成最长的一个

dev.off()

###############

gloci<-read.table("loci_MAPK6.bed",header=F,as.is=T)

head(gloci)

#可调整的参数

genefold<-as.numeric("1.5")#放大、缩小展示的范围

# 展示的基因组范围

colnames(gloci)<-c("chr","start","end","strand")

chr<-gloci[rownames(gloci),]$chr

gloci$width<-with(gloci,end-start)

startpoint<-gloci[rownames(gloci),]$start-genefold*gloci[rownames(gloci),]$width

endpoint<-gloci[rownames(gloci),]$end+genefold*gloci[rownames(gloci),]$width

#下面将scale等track写入tracklist

tracklist<-list()

#写入chromosome

itrack <- IdeogramTrack(genome = "hg38", chromosome = chr,outline=T)

tracklist[["itrack"]]<-itrack

#写入比例尺

scalebar <- GenomeAxisTrack(scale=0.25,col="black",fontcolor="black",name="Scale",labelPos="above",showTitle=TRUE)

tracklist[["scalebar"]]<-scalebar

#写入基因组位置

axisTrack <- GenomeAxisTrack(labelPos="above",col="black",fontcolor="black",name=paste(chr,":",sep=""),exponent=0,showTitle=TRUE)

tracklist[["axisTrack"]]<-axisTrack

#写入bigwig

#配色

colpal<-rep(brewer.pal(12,"Paired"),20)

coldf<-data.frame(col=colpal[1:nrow(bwInfo)],row.names = rownames(bwInfo),stringsAsFactors = F)

for(index in rownames(bwInfo)){

bgFile<-file.path("F:\\TCGA-ATAC-seq_bigwig\\GSE139099",paste(index,".bw",sep=""))

tracklist[[index]]<-DataTrack(range = bgFile,genome="hg38",type="histogram",

name=chartr("_","\n",bwInfo[index,]),

col.histogram=coldf[index,])#每个track颜色不同才好看

}

#写入基因结构

tracklist[["grt"]]<-grt

#输出pdf文件

pdf("loci_MAPK6.pdf",height=20,width=10)

plotTracks(tracklist, from = startpoint, to = endpoint,

chromosome=chr,background.panel = "white", background.title = "white",

col.title="black",col.axis="black",ylim=c(0,50),

rot.title=0,cex.title=0.9,margin=38,title.width=1.5,collapseTranscripts = "longest")

#collapseTranscripts = "longest")#同一个基因的多个transcript压缩成最长的一个

dev.off()

###############

gloci<-read.table("loci_PCCA.bed",header=F,as.is=T)

head(gloci)

#可调整的参数

genefold<-as.numeric("1.5")#放大、缩小展示的范围

# 展示的基因组范围

colnames(gloci)<-c("chr","start","end","strand")

chr<-gloci[rownames(gloci),]$chr

gloci$width<-with(gloci,end-start)

startpoint<-gloci[rownames(gloci),]$start-genefold*gloci[rownames(gloci),]$width

endpoint<-gloci[rownames(gloci),]$end+genefold*gloci[rownames(gloci),]$width

#下面将scale等track写入tracklist

tracklist<-list()

#写入chromosome

itrack <- IdeogramTrack(genome = "hg38", chromosome = chr,outline=T)

tracklist[["itrack"]]<-itrack

#写入比例尺

scalebar <- GenomeAxisTrack(scale=0.25,col="black",fontcolor="black",name="Scale",labelPos="above",showTitle=TRUE)

tracklist[["scalebar"]]<-scalebar

#写入基因组位置

axisTrack <- GenomeAxisTrack(labelPos="above",col="black",fontcolor="black",name=paste(chr,":",sep=""),exponent=0,showTitle=TRUE)

tracklist[["axisTrack"]]<-axisTrack

#写入bigwig

#配色

colpal<-rep(brewer.pal(12,"Paired"),20)

coldf<-data.frame(col=colpal[1:nrow(bwInfo)],row.names = rownames(bwInfo),stringsAsFactors = F)

for(index in rownames(bwInfo)){

bgFile<-file.path("F:\\TCGA-ATAC-seq_bigwig\\GSE139099",paste(index,".bw",sep=""))

tracklist[[index]]<-DataTrack(range = bgFile,genome="hg38",type="histogram",

name=chartr("_","\n",bwInfo[index,]),

col.histogram=coldf[index,])#每个track颜色不同才好看

}

#写入基因结构

tracklist[["grt"]]<-grt

#输出pdf文件

pdf("loci_PCCA.pdf",height=20,width=10)

plotTracks(tracklist, from = startpoint, to = endpoint,

chromosome=chr,background.panel = "white", background.title = "white",

col.title="black",col.axis="black",ylim=c(0,50),

rot.title=0,cex.title=0.9,margin=38,title.width=1.5,collapseTranscripts = "longest")

#collapseTranscripts = "longest")#同一个基因的多个transcript压缩成最长的一个

dev.off()

###############

gloci<-read.table("loci_PRRC2C.bed",header=F,as.is=T)

head(gloci)

#可调整的参数

genefold<-as.numeric("1.5")#放大、缩小展示的范围

# 展示的基因组范围

colnames(gloci)<-c("chr","start","end","strand")

chr<-gloci[rownames(gloci),]$chr

gloci$width<-with(gloci,end-start)

startpoint<-gloci[rownames(gloci),]$start-genefold*gloci[rownames(gloci),]$width

endpoint<-gloci[rownames(gloci),]$end+genefold*gloci[rownames(gloci),]$width

#下面将scale等track写入tracklist

tracklist<-list()

#写入chromosome

itrack <- IdeogramTrack(genome = "hg38", chromosome = chr,outline=T)

tracklist[["itrack"]]<-itrack

#写入比例尺

scalebar <- GenomeAxisTrack(scale=0.25,col="black",fontcolor="black",name="Scale",labelPos="above",showTitle=TRUE)

tracklist[["scalebar"]]<-scalebar

#写入基因组位置

axisTrack <- GenomeAxisTrack(labelPos="above",col="black",fontcolor="black",name=paste(chr,":",sep=""),exponent=0,showTitle=TRUE)

tracklist[["axisTrack"]]<-axisTrack

#写入bigwig

#配色

colpal<-rep(brewer.pal(12,"Paired"),20)

coldf<-data.frame(col=colpal[1:nrow(bwInfo)],row.names = rownames(bwInfo),stringsAsFactors = F)

for(index in rownames(bwInfo)){

bgFile<-file.path("F:\\TCGA-ATAC-seq_bigwig\\GSE139099",paste(index,".bw",sep=""))

tracklist[[index]]<-DataTrack(range = bgFile,genome="hg38",type="histogram",

name=chartr("_","\n",bwInfo[index,]),

col.histogram=coldf[index,])#每个track颜色不同才好看

}

#写入基因结构

tracklist[["grt"]]<-grt

#输出pdf文件

pdf("loci_PRRC2C.pdf",height=20,width=10)

plotTracks(tracklist, from = startpoint, to = endpoint,

chromosome=chr,background.panel = "white", background.title = "white",

col.title="black",col.axis="black",ylim=c(0,50),

rot.title=0,cex.title=0.9,margin=38,title.width=1.5,collapseTranscripts = "longest")

#collapseTranscripts = "longest")#同一个基因的多个transcript压缩成最长的一个

dev.off()

###############

gloci<-read.table("loci_USP3.bed",header=F,as.is=T)

head(gloci)

#可调整的参数

genefold<-as.numeric("1.5")#放大、缩小展示的范围

# 展示的基因组范围

colnames(gloci)<-c("chr","start","end","strand")

chr<-gloci[rownames(gloci),]$chr

gloci$width<-with(gloci,end-start)

startpoint<-gloci[rownames(gloci),]$start-genefold*gloci[rownames(gloci),]$width

endpoint<-gloci[rownames(gloci),]$end+genefold*gloci[rownames(gloci),]$width

#下面将scale等track写入tracklist

tracklist<-list()

#写入chromosome

itrack <- IdeogramTrack(genome = "hg38", chromosome = chr,outline=T)

tracklist[["itrack"]]<-itrack

#写入比例尺

scalebar <- GenomeAxisTrack(scale=0.25,col="black",fontcolor="black",name="Scale",labelPos="above",showTitle=TRUE)

tracklist[["scalebar"]]<-scalebar

#写入基因组位置

axisTrack <- GenomeAxisTrack(labelPos="above",col="black",fontcolor="black",name=paste(chr,":",sep=""),exponent=0,showTitle=TRUE)

tracklist[["axisTrack"]]<-axisTrack

#写入bigwig

#配色

colpal<-rep(brewer.pal(12,"Paired"),20)

coldf<-data.frame(col=colpal[1:nrow(bwInfo)],row.names = rownames(bwInfo),stringsAsFactors = F)

for(index in rownames(bwInfo)){

bgFile<-file.path("F:\\TCGA-ATAC-seq_bigwig\\GSE139099",paste(index,".bw",sep=""))

tracklist[[index]]<-DataTrack(range = bgFile,genome="hg38",type="histogram",

name=chartr("_","\n",bwInfo[index,]),

col.histogram=coldf[index,])#每个track颜色不同才好看

}

#写入基因结构

tracklist[["grt"]]<-grt

#输出pdf文件

pdf("loci_USP3.pdf",height=20,width=10)

plotTracks(tracklist, from = startpoint, to = endpoint,

chromosome=chr,background.panel = "white", background.title = "white",

col.title="black",col.axis="black",ylim=c(0,50),

rot.title=0,cex.title=0.9,margin=38,title.width=1.5,collapseTranscripts = "longest")

#collapseTranscripts = "longest")#同一个基因的多个transcript压缩成最长的一个

dev.off()

###############

gloci<-read.table("loci_VOPP1.bed",header=F,as.is=T)

head(gloci)

#可调整的参数

genefold<-as.numeric("1.5")#放大、缩小展示的范围

# 展示的基因组范围

colnames(gloci)<-c("chr","start","end","strand")

chr<-gloci[rownames(gloci),]$chr

gloci$width<-with(gloci,end-start)

startpoint<-gloci[rownames(gloci),]$start-genefold*gloci[rownames(gloci),]$width

endpoint<-gloci[rownames(gloci),]$end+genefold*gloci[rownames(gloci),]$width

#下面将scale等track写入tracklist

tracklist<-list()

#写入chromosome

itrack <- IdeogramTrack(genome = "hg38", chromosome = chr,outline=T)

tracklist[["itrack"]]<-itrack

#写入比例尺

scalebar <- GenomeAxisTrack(scale=0.25,col="black",fontcolor="black",name="Scale",labelPos="above",showTitle=TRUE)

tracklist[["scalebar"]]<-scalebar

#写入基因组位置

axisTrack <- GenomeAxisTrack(labelPos="above",col="black",fontcolor="black",name=paste(chr,":",sep=""),exponent=0,showTitle=TRUE)

tracklist[["axisTrack"]]<-axisTrack

#写入bigwig

#配色

colpal<-rep(brewer.pal(12,"Paired"),20)

coldf<-data.frame(col=colpal[1:nrow(bwInfo)],row.names = rownames(bwInfo),stringsAsFactors = F)

for(index in rownames(bwInfo)){

bgFile<-file.path("F:\\TCGA-ATAC-seq_bigwig\\GSE139099",paste(index,".bw",sep=""))

tracklist[[index]]<-DataTrack(range = bgFile,genome="hg38",type="histogram",

name=chartr("_","\n",bwInfo[index,]),

col.histogram=coldf[index,])#每个track颜色不同才好看

}

#写入基因结构

tracklist[["grt"]]<-grt

#输出pdf文件

pdf("loci_VOPP1.pdf",height=20,width=10)

plotTracks(tracklist, from = startpoint, to = endpoint,

chromosome=chr,background.panel = "white", background.title = "white",

col.title="black",col.axis="black",ylim=c(0,50),

rot.title=0,cex.title=0.9,margin=38,title.width=1.5,collapseTranscripts = "longest")

#collapseTranscripts = "longest")#同一个基因的多个transcript压缩成最长的一个

dev.off()
